# Supplementary material for: Discovery and Mechanism of Novel 7-Aliphatic Amine Tryptanthrin Derivatives against Phytopathogenic Bacteria
Source: Int J Mol Sci. 2023 Jun 30;24(13):10900. doi: 10.3390/ijms241310900 (PMC10341529; doi:10.3390/ijms241310900)
Supplement: Supplementary file 1 [file ijms-24-10900-s001.zip › ijms-2423804-supplementary.pdf]

Discovery and Mechanism of Novel 7-aliphatic Amines Tryptanthrin Derivatives Against Phytopathogenic Bacteria

Xuesha Long [1†], Guanglong Zhang [2†], Haitao Long [1], Qin Wang [1], Congyu Wang [1], Mei Zhu[1], Wenhang Wang [1], Chengpeng Li [1], Zhenchao Wang [1,2,3,*], Guiping Ouyang [1,2,3,*]

[1] School of Pharmaceutical Sciences, Guizhou University, Guiyang 550025, China; gs.xslong20@gzu.edu.cn (X.L.); gs.hylong21@gzu.edu.cn (H.L.); wq18385365070@163.com (Q.W.); gs.congyuwang20@gzu.edu.cn (C.W.); gs.mzhu20@gzu.edu.cn (M.Z.); whwang2302@163.com (W.W.); lichp11@163.com (C.L.)

[2] National Key Laboratory of Green Pesticide, Key Laboratory of Green Pesticide and Agricultural Bioengineering, Ministry of Education, Center for R&D of Fine Chemicals of Guizhou University, Guiyang 550025, China; glz2593195@126.com

[3] Guizhou Engineering Laboratory for Synthetic Drugs, Guizhou University, Guiyang 550025, China

* Correspondence: zcwang@gzu.edu.cn (Z.W.); gpouyang@gzu.edu.cn (G.O.); Tel./Fax: +86-851-8830-8717 (G.O.)

† These authors contributed equally to this work

Citation: Long, X.; Zhang, G.; Long, H.; Wang, Q.; Wang, C.; Zhu, M.; Wang, W.; Li, C.; Wang, Z.; Ouyang, G. Discovery and Mechanism of Novel 7-Aliphatic Amine Tryptanthrin Derivatives against Phytopathogenic Bacteria. *Int. J. Mol. Sci.* **2023**, *24*, 10900. https://doi.org/10.3390/ijms241310900

Received: 14 May 2023

Revised: 23 June 2023

Accepted: 27 June 2023

Published: 30 June 2023

Copyright: © 2023 by the authors. Submitted for possible open access publication under the terms and conditions of the Creative Commons Attribution (CC BY) license (https://creativecommons.org/licenses/by/4.0/).

Table of Contents

1.Characterization data of the title compounds...S2

2.^{1}H NMR, ^{13}C NMR, and HRMS spectrum of the title compounds S10

Characterization data of the title compounds:

7-((2-(dimethylamino)ethyl)amino)indolo[2,1-b]quinazoline-6,12-dione(7a).

Orange red solid, yield 63.5%, m. p. 195-196°C. ^{1}H NMR (400 MHz, Chloroform-*d*) δ 8.43 (dd, *J* = 7.9, 1.6 Hz, 1H), 8.01 (d, *J* = 8.1 Hz, 1H), 7.83 (td, *J* = 7.7, 1.6 Hz, 1H), 7.74 – 7.58 (m, 3H), 7.49 (t, *J* = 8.1 Hz, 1H), 6.60 (d, *J* = 8.7 Hz, 1H), 3.46 (q, *J* = 6.2 Hz, 2H), 2.65 (t, *J* = 6.5 Hz, 2H), 2.35 (s, 6H). ^{13}C NMR (101 MHz, Chloroform-*d*) δ 180.73, 158.38, 149.34, 146.91, 146.05, 144.79, 140.00, 134.73, 130.21, 129.33, 127.36, 123.26, 110.01, 105.81, 104.62, 57.67, 45.45, 40.52. ESI-HRMS, m/z[M+H]$^+$ calculated for $C_{19}H_{19}N_4O_2$, 335.1503; found, 335.1504.

7-((3-(dimethylamino)propyl)amino)indolo[2,1-b]quinazoline-6,12-dione(7b).

Orange red solid, yield 47.6%, m. p. 162-163°C. ^{1}H NMR (400 MHz, Chloroform-*d*) δ 8.42 (dd, *J* = 8.0, 1.6 Hz, 1H), 8.10 (t, *J* = 5.7 Hz, 1H), 8.02 (dd, *J* = 8.2, 1.2 Hz, 1H), 7.82 (td, *J* = 8.2, 7.7, 1.6 Hz, 1H), 7.70 – 7.56 (m, 2H), 7.47 (t, *J* = 8.2 Hz, 1H), 6.62 (d, *J* = 8.8 Hz, 1H), 3.48 (q, *J* = 6.4 Hz, 2H), 2.50 (t, *J* = 6.5 Hz, 2H), 2.34 (s, 6H), 1.90 (p, *J* = 6.6 Hz, 2H). ^{13}C NMR (101 MHz, Chloroform-*d*) δ 180.51, 158.36, 149.42, 146.90, 144.77, 139.89, 134.71, 130.13, 129.27, 127.35, 123.25, 109.99, 105.67, 104.41, 57.41, 45.32, 41.69, 26.43. ESI-HRMS, m/z [M+H]$^+$ calculated for $C_{20}H_{21}N_4O_2$, 349.1659; found, 349.1661.

7-((3-(diethylamino)propyl)amino)indolo[2,1-b]quinazoline-6,12-dione(7c).

Fuchsia solid, yield 39.5%, m. p. 112-113°C. ^{1}H NMR (400 MHz, Chloroform-*d*) δ 8.43 (dd, *J* = 7.9, 1.6 Hz, 1H), 8.07 – 7.97 (m, 1H), 7.84 (td, *J* = 8.2, 7.8, 1.6 Hz, 1H), 7.72 (d, *J* = 7.6 Hz, 1H), 7.69 – 7.61 (m, 1H), 7.60 – 7.47 (m, 2H), 6.68 (d, *J* = 8.6 Hz, 1H), 3.57 (d, *J* = 6.3 Hz, 2H), 2.99 (d, *J* = 8.1 Hz, 6H), 2.22 – 2.14 (m, 2H), 1.31 (t, *J* = 7.2 Hz, 6H). ^{13}C NMR (101 MHz, Chloroform-*d*) δ 180.67, 149.24, 146.80, 146.01, 144.72, 140.06, 134.75, 130.11, 129.36, 127.36, 123.22, 109.96, 105.66, 104.71, 50.11, 46.83, 41.24, 25.64, 10.48. ESI-HRMS, m/z [M+H]$^+$ calculated for $C_{22}H_{25}N_4O_2$, 377.1972; found, 377.1973.

7-((2-(dimethylamino)ethyl)amino)-2-fluoroindolo[2,1-b]quinazoline-6,12-dione(7d). Orange solid, yield 36.4%, m. p. 237-238°C. ^{1}H NMR (400 MHz,

Chloroform-*d*) δ 8.05 (ddd, *J* = 19.7, 8.9, 3.9 Hz, 2H), 7.69 (d, *J* = 7.9 Hz, 2H), 7.52 (dt, *J* = 16.1, 8.2 Hz, 2H), 6.62 (d, *J* = 8.8 Hz, 1H), 3.54 – 3.36 (m, 2H), 2.65 (t, *J* = 6.4 Hz, 2H), 2.35 (s, 6H).^{13}C NMR (101 MHz, Chloroform-*d*) δ 180.39, 163.82, 161.31, 149.37, 144.49, 143.51, 139.98, 132.58, 123.13, 113.01, 110.27, 105.76, 104.66, 57.63, 45.44, 40.50. ESI-HRMS, m/z [M+H]$^+$ calculated for $C_{19}H_{18}FN_4O_2$, 335.1503; found, 335.1504.

7-((3-(dimethylamino)propyl)amino)-2-fluoroindolo[2,1-b]quinazoline-6,12-dione(7e). Orange solid, yield 35.3%, m. p. 196-197℃. ^{1}H NMR (400 MHz, Chloroform-*d*) δ 8.29 – 7.81 (m, 3H), 7.67 (d, *J* = 7.7 Hz, 1H), 7.59 – 7.40 (m, 2H), 6.64 (d, *J* = 8.8 Hz, 1H), 3.48 (q, *J* = 6.2 Hz, 2H), 2.49 (t, *J* = 6.3 Hz, 2H), 2.33 (s, 6H), 1.95 – 1.87 (m, 2H).^{13}C NMR (101 MHz, Chloroform-*d*) δ 180.17, 159.39 (d, *J* = 378.7 Hz), 149.48, 144.51, 139.86, 132.50, 122.87, 112.76, 110.26, 104.43, 57.48, 45.38, 41.82, 26.44. ESI-HRMS, m/z [M+H]$^+$ calculated for $C_{20}H_{20}FN_4O_2$, 367.1565; found, 367.1566.

7-((3-(diethylamino)propyl)amino)-2-fluoroindolo[2,1-b]quinazoline-6,12-dione(7f). Dark red solid, yield 40.9%, m. p. 161-162℃. ^{1}H NMR (400 MHz, Chloroform-*d*) δ 8.12 – 7.99 (m, 2H), 7.87 (s, 1H), 7.70 (d, *J* = 7.6 Hz, 1H), 7.59 – 7.44 (m, 2H), 6.68 (d, *J* = 8.7 Hz, 1H), 3.52 (d, *J* = 6.2 Hz, 2H), 2.76 (d, *J* = 8.6 Hz, 6H), 2.05 – 1.96 (m, 2H), 1.29 (d, *J* = 12.6 Hz, 2H), 1.17 (t, *J* = 7.2 Hz, 4H). ^{13}C NMR (101 MHz, Chloroform-*d*) δ 180.23, 149.30, 144.42, 143.42, 139.92, 132.49, 123.10, 112.98, 110.22, 105.59, 104.60, 50.26, 46.90, 41.52, 25.92, 10.85.ESI-HRMS, m/z [M+H]$^+$ calculated for $C_{22}H_{24}FN_4O_2$, 395.1878; found, 395.1881.

2-bromo-7-((3-(dimethylamino)propyl)amino)indolo[2,1-b]quinazoline-6,12-dione(7g). Orange solid, yield 32.6%, m. p. 217-218℃. ^{1}H NMR (400 MHz, Chloroform-*d*) δ 8.54 (d, *J* = 2.1 Hz, 1H), 8.10 (t, *J* = 5.5 Hz, 1H), 7.97 – 7.82 (m, 2H), 7.65 (d, *J* = 7.5 Hz, 1H), 7.47 (t, *J* = 8.2 Hz, 1H), 6.64 (d, *J* = 8.8 Hz, 1H), 3.50 (q, *J* = 6.3 Hz, 2H), 2.58 (t, *J* = 6.6 Hz, 2H), 2.40 (s, 6H), 1.94 (p, *J* = 6.6 Hz, 2H).^{13}C NMR (101 MHz, Chloroform-*d*) δ 180.10, 157.06, 149.42, 146.31, 145.73, 144.41, 139.99, 137.90, 131.62, 130.07, 124.59, 123.43, 110.30, 105.66, 104.60, 57.26, 45.10, 41.63,

26.20. ESI-HRMS, m/z $[M+H]^+$ calculated for $C_{20}H_{20}BrN_4O_2$, 427.0764; found, 427.0768.

7-((2-(dimethylamino)ethyl)amino)-2-methoxyindolo[2,1-b]quinazoline-6,12-dione(7h). Orange Solid, yield 21.6%, m. p. 207-209°C. ^{1}H NMR (400 MHz, Chloroform-*d*) δ 7.93 (d, J = 8.9 Hz, 1H), 7.82 (d, J = 2.9 Hz, 1H), 7.75 (d, J = 7.6 Hz, 1H), 7.58 – 7.46 (m, 2H), 7.39 (dd, J = 8.9, 3.0 Hz, 1H), 6.74 (d, J = 8.7 Hz, 1H), 3.99 (s, 3H), 3.73 (s, 2H), 2.99 (d, J = 12.3 Hz, 2H), 2.67 (s, 6H).^{13}C NMR (101 MHz, Chloroform-*d*) δ 181.27, 160.75, 158.05, 144.82, 141.01, 140.30, 131.84, 124.59, 124.18, 109.81, 107.94, 106.29, 105.44, 57.00, 56.04, 44.60, 29.71. ESI-HRMS, m/z $[M+H]^+$ calculated for $C_{20}H_{21}N_4O_3$, 365.1608; found, 365.1609.

7-((3-(dimethylamino)propyl)amino)-2-methoxyindolo[2,1-b]quinazoline-6,12-dione(7i). Orange red solid, yield 29.7%, m. p. 173-175°C. ^{1}H NMR (400 MHz, Chloroform-*d*) δ 7.94 (d, J = 8.9 Hz, 1H), 7.89 (s, 1H), 7.82 (d, J = 2.9 Hz, 1H), 7.70 (d, J = 7.5 Hz, 1H), 7.49 (dd, J = 8.7, 7.6 Hz, 1H), 7.39 (dd, J = 8.9, 3.0 Hz, 1H), 6.64 (d, J = 8.7 Hz, 1H), 3.98 (s, 3H), 3.50 (q, J = 6.4 Hz, 2H), 2.65 (t, J = 6.8 Hz, 2H), 2.46 (s, 6H), 1.98 (p, J = 6.8 Hz, 2H).^{13}C NMR (101 MHz, Chloroform-*d*) δ 180.82, 160.59, 158.13, 149.23, 144.74, 141.11, 139.83, 131.75, 124.54, 124.13, 109.86, 107.85, 105.94, 104.65, 57.02, 56.02, 44.87, 41.23, 26.16. ESI-HRMS, m/z $[M+H]^+$ calculated for $C_{21}H_{23}N_4O_3$, 379.1765; found, 379.1766.

7-((3-(dimethylamino)propyl)amino)-2-methylindolo[2,1-b]quinazoline-6,12-dione(7j). Orange red solid, yield 30.4%, m. p. 104-106°C. ^{1}H NMR (400 MHz, Chloroform-*d*) δ 8.21 (s, 1H), 8.05 – 7.80 (m, 2H), 7.66 (d, J = 23.7 Hz, 2H), 7.47 (s, 1H), 6.61 (d, J = 8.7 Hz, 1H), 3.49 (s, 2H), 2.56 (s, 3H), 2.40 (s, 6H), 1.93 (s, 2H), 1.27 (s, 2H).^{13}C NMR (101 MHz, Chloroform-*d*) δ 180.75, 158.38, 149.31, 144.82, 140.10, 139.88, 136.03, 129.98, 127.09, 123.00, 109.82, 105.80, 104.52, 57.21, 45.10, 41.45, 26.31, 21.59. ESI-HRMS, m/z $[M+H]^+$ calculated for $C_{21}H_{23}N_4O_2$, 363.1816; found, 363.1817.

7-((3-(dimethylamino)propyl)amino)-2-nitroindolo[2,1-b]quinazoline-6,12-dione(7k). Purple red solid, yield 22.8%, m. p. >300°C. ^{1}H NMR (400 MHz,

Chloroform-*d*) δ 7.81 (d, *J* = 8.2 Hz, 2H), 7.69 (d, *J* = 7.4 Hz, 1H), 7.58 (d, *J* = 3.0 Hz, 1H), 7.46 (t, *J* = 8.0 Hz, 1H), 7.10 (dd, *J* = 8.7, 2.7 Hz, 1H), 6.61 (d, *J* = 9.1 Hz, 1H), 3.51 – 3.45 (m, 2H), 2.62 (s, 2H), 2.43 (s, 6H), 2.00 – 1.92 (m, 2H). ^{13}C NMR (101 MHz, Chloroform-*d*) δ 180.18, 157.07, 149.40, 145.73, 144.43, 140.06, 137.92, 131.63, 130.09, 124.59, 123.47, 110.29, 105.69, 104.69, 57.15, 44.97, 41.51, 26.11. ESI-HRMS, m/z $[M+H]^+$ calculated for $C_{20}H_{20}N_5O_4$, 394.1510; found, 394.1517.

7-(piperazin-1-yl)indolo[2,1-b]quinazoline-6,12-dione(7l). Red solid, yield 52.7%, m. p. 242-243°C.^{1}H NMR (400 MHz, Chloroform-*d*) δ 8.44 – 8.37 (m, 1H), 8.07 (dd, *J* = 7.7, 0.7 Hz, 1H), 8.01 – 7.97 (m, 1H), 7.82 (ddd, *J* = 8.1, 7.2, 1.6 Hz, 1H), 7.63 (ddd, *J* = 8.3, 7.2, 1.2 Hz, 1H), 7.55 (dd, *J* = 8.7, 7.7 Hz, 1H), 6.81 (d, *J* = 8.5 Hz, 1H), 3.46 – 3.39 (m, 4H), 3.18 – 3.11 (m, 4H). ^{13}C NMR (101 MHz, Chloroform-*d*) δ 178.16, 158.25, 152.17, 147.12, 146.99, 145.33, 138.90, 134.83, 130.23, 129.37, 127.40, 123.40, 115.52, 110.30, 108.03, 52.18, 46.05. ESI-HRMS, m/z $[M+H]^+$ calculated for $C_{19}H_{17}N_4O_2$, 333.1346; found, 333.1335.

7-(4-methylpiperazin-1-yl)indolo[2,1-b]quinazoline-6,12-dione(7m). Red solid, yield 76.4%, m. p. 237-238°C. ^{1}H NMR (400 MHz, Chloroform-*d*) δ 8.38 (d, *J* = 7.9 Hz, 1H), 8.00 (dd, *J* = 24.1, 7.9 Hz, 2H), 7.80 (t, *J* = 7.6 Hz, 1H), 7.60 (t, *J* = 7.5 Hz, 1H), 7.50 (t, *J* = 8.2 Hz, 1H), 6.77 (d, *J* = 8.6 Hz, 1H), 3.61 – 3.30 (m, 4H), 2.80 – 2.59 (m, 4H), 2.40 (s, 3H). ^{13}C NMR (101 MHz, Chloroform-*d*) δ 178.13, 158.11, 151.72, 147.04, 146.90, 145.22, 138.83, 134.78, 130.17, 129.33, 127.36, 123.35, 115.57, 110.25, 108.06, 54.82, 50.66, 46.04. ESI-HRMS, m/z $[M+H]^+$ calculated for $C_{20}H_{19}N_4O_2$, 347.1503; found,347.1491.

7-morpholinoindolo[2,1-b]quinazoline-6,12-dione(7n). Deep red solid, yield 81.6%, m. p. 267-268°C. ^{1}H NMR (400 MHz, Chloroform-*d*) δ 8.46 – 8.38 (m, 1H), 8.13 (dd, *J* = 7.7, 0.7 Hz, 1H), 8.05 – 7.98 (m, 1H), 7.84 (ddd, *J* = 8.1, 7.2, 1.6 Hz, 1H), 7.68 – 7.55 (m, 2H), 6.81 (dd, *J* = 8.7, 0.7 Hz, 1H), 4.04 – 3.95 (m, 4H), 3.50 – 3.41 (m, 4H). ^{13}C NMR (101 MHz, Chloroform-*d*) δ 178.41, 158.19, 151.83, 147.23, 146.91, 145.15, 139.13, 134.90, 130.28, 129.49, 127.43, 123.41, 115.19, 110.52, 108.59, 66.80, 51.09. ESI-HRMS, m/z $[M+H]^+$ calculated for $C_{19}H_{16}N_3O_3$, 334.1186; found, 334.1172.

2-fluoro-7-(piperazin-1-yl)indolo[2,1-b]quinazoline-6,12-dione(7o). Deep red solid, yield 40.5%, m. p. 254-255°C. ^{1}H NMR (400 MHz, Chloroform-*d*) δ 8.12 – 8.03 (m, 2H), 8.01 (dd, J = 8.9, 4.9 Hz, 1H), 7.61 – 7.50 (m, 2H), 6.83 (d, J = 8.6 Hz, 1H), 3.49 – 3.39 (m, 4H), 3.22 – 3.12 (m, 4H).^{13}C NMR (101 MHz, Chloroform-*d*) δ 177.81, 161.32, 157.36, 152.18, 146.81, 143.59, 138.88, 132.60, 132.51, 125.14, 123.25, 115.76, 113.05, 110.25, 108.02, 52.18, 46.04. ESI-HRMS, m/z $[M+H]^+$ calculated for $C_{19}H_{16}FN_4O_2$, 351.1252; found, 351.1252.

2-fluoro-7-morpholinoindolo[2,1-b]quinazoline-6,12-dione(7p). Orange red solid, yield 33.5%, m. p. 278-279°C. ^{1}H NMR (400 MHz, Chloroform-*d*) δ 8.12 – 8.03 (m, 2H), 8.01 (dd, J = 8.9, 4.9 Hz, 1H), 7.63 – 7.51 (m, 2H), 6.83 (d, J = 8.6 Hz, 1H), 3.99 (dd, J = 5.8, 3.6 Hz, 4H), 3.49 – 3.43 (m, 4H).^{13}C NMR (101 MHz, Chloroform-*d*) δ 178.08, 161.39, 157.31, 151.86, 146.94, 139.12, 132.58, 123.31, 115.44, 113.11, 110.48, 108.59, 66.78, 51.09.ESI-HRMS, m/z $[M+H]^+$ calculated for $C_{19}H_{15}FN_3O_3$, 352.1092; found, 352.1079.

2-chloro-7-(piperazin-1-yl)indolo[2,1-b]quinazoline-6,12-dione(7q). Dark red solid, yield 36.1%, m. p. >300°C. ^{1}H NMR (400 MHz, Chloroform-*d*) δ 8.10 (d, J = 7.7 Hz, 1H), 7.90 (d, J = 8.9 Hz, 1H), 7.78 (d, J = 3.0 Hz, 1H), 7.56 (t, J = 8.1 Hz, 1H), 7.38 (dd, J = 8.9, 3.0 Hz, 1H), 6.79 (d, J = 8.6 Hz, 1H), 3.98 (s, 4H), 3.43 (t, J = 4.6 Hz, 4H).^{13}C NMR (101 MHz, Chloroform-*d*) δ 178.41, 160.67, 157.91, 151.71, 147.10, 143.34, 141.12, 138.85, 131.86, 124.67, 124.21, 115.14, 110.84, 108.66, 107.89, 66.80, 56.03, 51.09.ESI-HRMS, m/z $[M+H]^+$ calculated for $C_{19}H_{16}ClN_4O_2$, 367.0956; found, 367.0962.

2-chloro-7-(4-methylpiperazin-1-yl)indolo[2,1-b]quinazoline-6,12-dione(7r). Orange red solid, yield 42.9%, m. p. 274-276°C. ^{1}H NMR (400 MHz, Chloroform-*d*) δ 8.40 (d, J = 2.4 Hz, 1H), 8.14 (d, J = 7.7 Hz, 1H), 7.95 (d, J = 8.6 Hz, 1H), 7.78 (dd, J = 8.6, 2.4 Hz, 1H), 7.66 – 7.58 (m, 1H), 6.85 (d, J = 8.6 Hz, 1H), 3.63 (t, J = 4.9 Hz, 4H), 3.08 (s, 4H), 2.67 (s, 3H).^{13}C NMR (101 MHz, Chloroform-*d*) δ 178.19, 157.01, 146.90, 145.33, 139.46, 135.90, 135.32, 131.68, 127.08, 124.56, 115.92, 54.32, 49.41, 44.98.ESI-HRMS, m/z $[M+H]^+$ calculated for $C_{20}H_{18}ClN_4O_2$, 381.1113; found,

381.1114.

2-bromo-7-(piperazin-1-yl)indolo[2,1-b]quinazoline-6,12-dione(7s). Orange red solid, yield 41.3%, m. p. >300°C. ^{1}H NMR (400 MHz, Chloroform-*d*) δ 8.57 (d, J = 2.3 Hz, 1H), 8.04 (s, 1H), 7.94 – 7.85 (m, 2H), 7.61 – 7.54 (m, 1H), 6.84 (d, J = 8.6 Hz, 1H), 3.51 – 3.42 (m, 4H), 3.19 (t, J = 5.0 Hz, 4H).^{13}C NMR (101 MHz, Chloroform-*d*) δ 178.16, 152.17, 147.12, 146.99, 138.90, 134.83, 130.23, 129.37, 127.40, 115.52, 108.03, 52.18, 46.05. ESI-HRMS, m/z [M+H]$^{+}$ calculated for $C_{19}H_{16}BrN_{4}O_{2}$, 411.0451; found, 411.0453.

2-bromo-7-(4-methylpiperazin-1-yl)indolo[2,1-b]quinazoline-6,12-dione(7t). Dark red solid, yield 47.0%, m. p. 267-269°C. ^{1}H NMR (400 MHz, Chloroform-*d*) δ 8.56 (d, J = 2.2 Hz, 1H), 8.10 (d, J = 7.7 Hz, 1H), 7.96 – 7.80 (m, 2H), 7.60 (dd, J = 8.6, 7.7 Hz, 1H), 6.84 (d, J = 8.6 Hz, 1H), 3.58 (t, J = 4.9 Hz, 4H), 2.96 (d, J = 14.2 Hz, 4H), 2.58 (s, 3H).^{13}C NMR (101 MHz, Chloroform-*d*) δ 178.04, 166.77, 156.91, 146.81, 145.34, 139.21, 138.09, 131.75, 130.17, 124.75, 115.92, 54.58, 50.08, 45.52. ESI-HRMS, m/z [M+H]$^{+}$ calculated for $C_{20}H_{18}BrN_{4}O_{2}$, 425.0608; found, 425.0612.

2-methoxy-7-(piperazin-1-yl)indolo[2,1-b]quinazoline-6,12-dione(7u). Orange solid, yield 61.8%, m. p. 246-248°C. ^{1}H NMR (400 MHz, Chloroform-*d*) δ 8.10 (d, J = 7.7 Hz, 1H), 7.93 (d, J = 8.9 Hz, 1H), 7.82 (d, J = 3.0 Hz, 1H), 7.57 (t, J = 8.2 Hz, 1H), 7.40 (dd, J = 9.0, 3.0 Hz, 1H), 6.82 (d, J = 8.6 Hz, 1H), 3.99 (s, 3H), 3.44 (t, J = 4.9 Hz, 4H), 3.17 (t, J = 4.8 Hz, 4H).^{13}C NMR (101 MHz, Chloroform-*d*) δ 178.23, 160.61, 158.03, 152.10, 147.04, 143.54, 141.25, 138.67, 131.83, 124.68, 124.23, 115.48, 110.66, 108.12, 107.84, 56.03, 52.20, 46.05. ESI-HRMS, m/z [M+H]$^{+}$ calculated for $C_{20}H_{19}N_{4}O_{3}$, 363.1452; found, 363.1452.

2-methoxy-7-(4-methylpiperazin-1-yl)indolo[2,1-b]quinazoline-6,12-dione(7v). Orange solid, yield 69.3%, m. p. 255-257°C. ^{1}H NMR (400 MHz, Chloroform-*d*) δ 8.14 (d, J = 7.7 Hz, 1H), 7.93 (d, J = 8.9 Hz, 1H), 7.82 (d, J = 3.0 Hz, 1H), 7.63 – 7.55 (m, 1H), 7.40 (dd, J = 8.9, 3.0 Hz, 1H), 6.83 (d, J = 8.5 Hz, 1H), 3.99 (s, 3H), 3.56 (t, J = 4.9 Hz, 4H), 2.94 (s, 4H), 2.57 (s, 3H).^{13}C NMR (101 MHz, Chloroform-*d*) δ 178.59, 160.75, 157.95, 151.30, 147.10, 143.34, 141.14, 138.96, 131.90, 124.72, 124.27,

115.59, 110.98, 108.87, 107.94, 56.04, 54.56, 45.43.ESI-HRMS, m/z $[M+H]^+$ calculated for $C_{21}H_{21}N_4O_3$, 377.1608; found, 377.1610.

2,3-dimethoxy-7-(piperazin-1-yl)indolo[2,1-b]quinazoline-6,12-dione(7w). Orange red solid, yield 59.6%, m. p. 217-219°C. ^1H NMR (400 MHz, Chloroform-d) δ 8.08 (d, J = 7.6 Hz, 1H), 8.03 (s, 1H), 7.75 (s, 1H), 7.55 (t, J = 8.1 Hz, 1H), 6.81 (d, J = 8.5 Hz, 1H), 4.04 (d, J = 11.2 Hz, 6H), 3.43 (t, J = 4.9 Hz, 4H), 3.16 (t, J = 4.7 Hz, 4H).^{13}C NMR (101 MHz, Chloroform-*d*) δ 178.26, 162.57, 152.09, 150.95, 147.23, 142.93, 138.73, 117.12, 115.44, 110.53, 108.13, 106.63, 56.51, 56.44, 52.12, 46.00, 36.50, 31.45.ESI-HRMS, m/z $[M+H]^+$ calculated for $C_{21}H_{21}N_4O_4$, 393.1557; found, 393.1561.

2,3-dimethoxy-7-(4-methylpiperazin-1-yl)indolo[2,1-b]quinazoline-6,12-dione(7x). Orange red solid, yield 67.4%, m. p. 256-258°C. ^1H NMR (400 MHz, Chloroform-*d*) δ 8.09 (d, J = 7.7 Hz, 1H), 7.73 (s, 1H), 7.55 (t, J = 8.1 Hz, 1H), 7.38 (s, 1H), 6.80 (d, J = 8.6 Hz, 1H), 4.04 (d, J = 10.3 Hz, 6H), 3.53 (t, J = 4.9 Hz, 4H), 2.84 (t, J = 4.8 Hz, 4H), 2.50 (s, 3H).^{13}C NMR (101 MHz, Chloroform-*d*) δ 178.49, 157.62, 154.93, 151.46, 151.00, 147.27, 144.23, 142.85, 138.87, 117.13, 115.52, 110.56, 108.59, 106.64, 56.51, 56.44, 54.64, 50.20, 45.63.ESI-HRMS, m/z $[M+H]^+$ calculated for $C_{22}H_{23}N_4O_4$, 407.1714; found, 407.1717.

2-methyl-7-(piperazin-1-yl)indolo[2,1-b]quinazoline-6,12-dione(7y). Orange red solid, yield 48.6%, m. p. 222-224°C. ^1H NMR (400 MHz, Chloroform-*d*) δ 8.22 (d, J = 2.0 Hz, 1H), 8.10 (d, J = 7.7 Hz, 1H), 7.89 (d, J = 8.3 Hz, 1H), 7.64 (dd, J = 8.3, 2.1 Hz, 1H), 7.56 (dd, J = 8.6, 7.7 Hz, 1H), 6.81 (d, J = 8.5 Hz, 1H), 3.48 – 3.38 (m, 4H), 3.25 – 3.14 (m, 4H), 2.56 (s, 3H).^{13}C NMR (101 MHz, Chloroform-*d*) δ 178.31, 158.25, 152.04, 147.15, 144.89, 144.68, 140.20, 138.84, 136.15, 130.06, 127.12, 123.14, 115.40, 110.50, 108.17, 51.99, 45.95, 21.61. ESI-HRMS, m/z $[M+H]^+$ calculated for $C_{20}H_{19}N_4O_2$, 347.1503; found, 347.1504.

2-nitro-7-(piperazin-1-yl)indolo[2,1-b]quinazoline-6,12-dione(7z). Purple red solid, yield 51.6%, m. p.>300°C. ^1H NMR (400 MHz, Chloroform-*d*) δ 9.29 (d, J = 2.6 Hz, 1H), 8.62 (dd, J = 9.0, 2.6 Hz, 1H), 8.15 (d, J = 8.8 Hz, 1H), 8.06 (d, J = 7.7 Hz, 1H),

7.60 (t, J = 8.1 Hz, 1H), 6.88 (d, J = 8.7 Hz, 1H), 3.50 (d, *J* = 6.2 Hz, 4H), 3.18 (t, *J* = 4.9 Hz, 4H). ^{13}C NMR (101 MHz, Chloroform-*d*) *δ* 152.14, 146.85, 138.95, 132.62, 123.27, 123.03, 115.77, 113.08, 112.84, 110.32, 108.14, 52.00, 50.90, 45.94. ESI-HRMS, m/z $[M+H]^+$ calculated for $C_{19}H_{16}N_5O_4$, 378.1197; found, 378.1198.

2. ^{1}H NMR, ^{13}C NMR and HRMS spectra for target compounds:

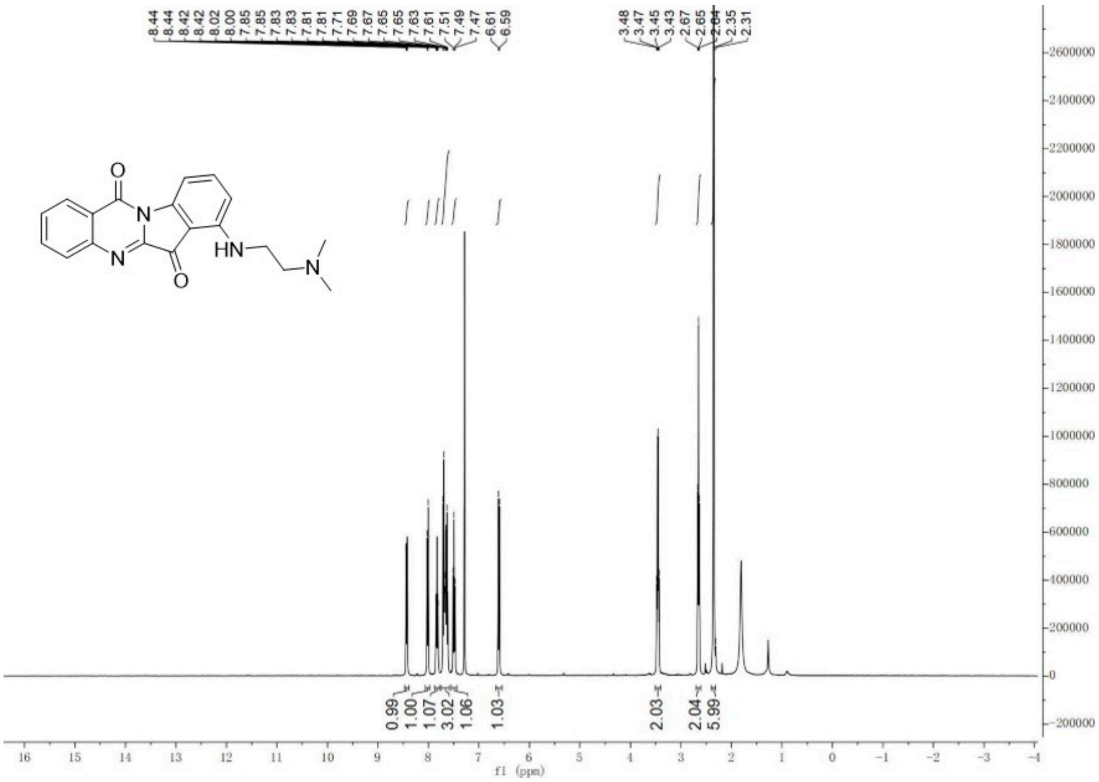

Figure S1. ^{1}H NMR Spectrum ($CDCl_3$, 400 MHz) of 7a.

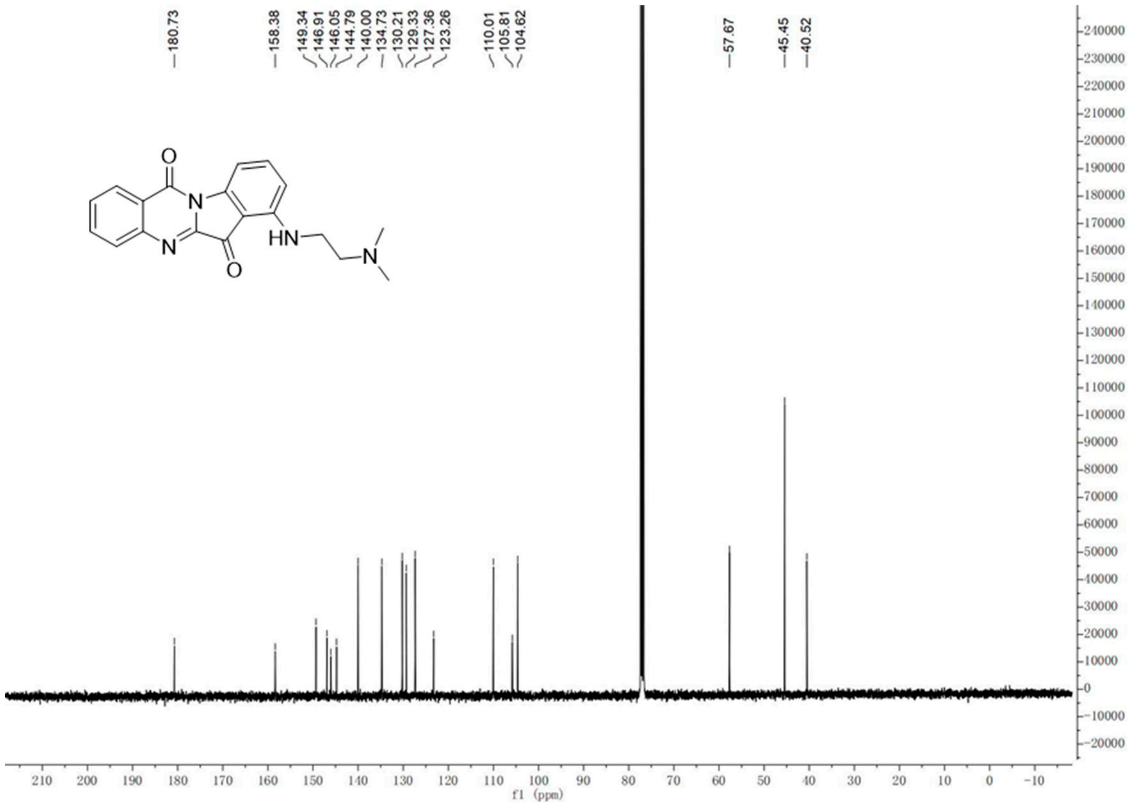

Figure S2. ^{13}C NMR Spectrum ($CDCl_3$, 101 MHz) of 7a.

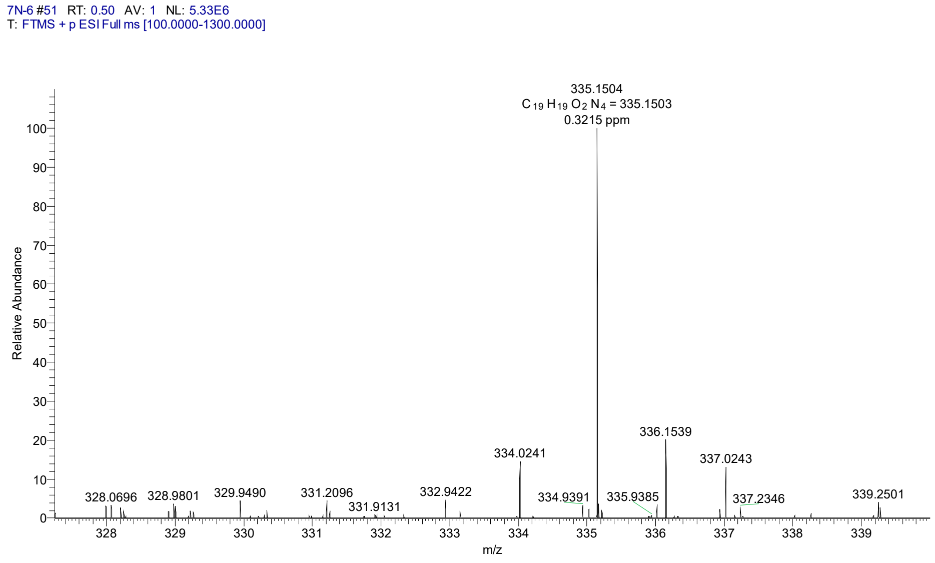

Figure S3. HR-MS Spectrum of 7a.

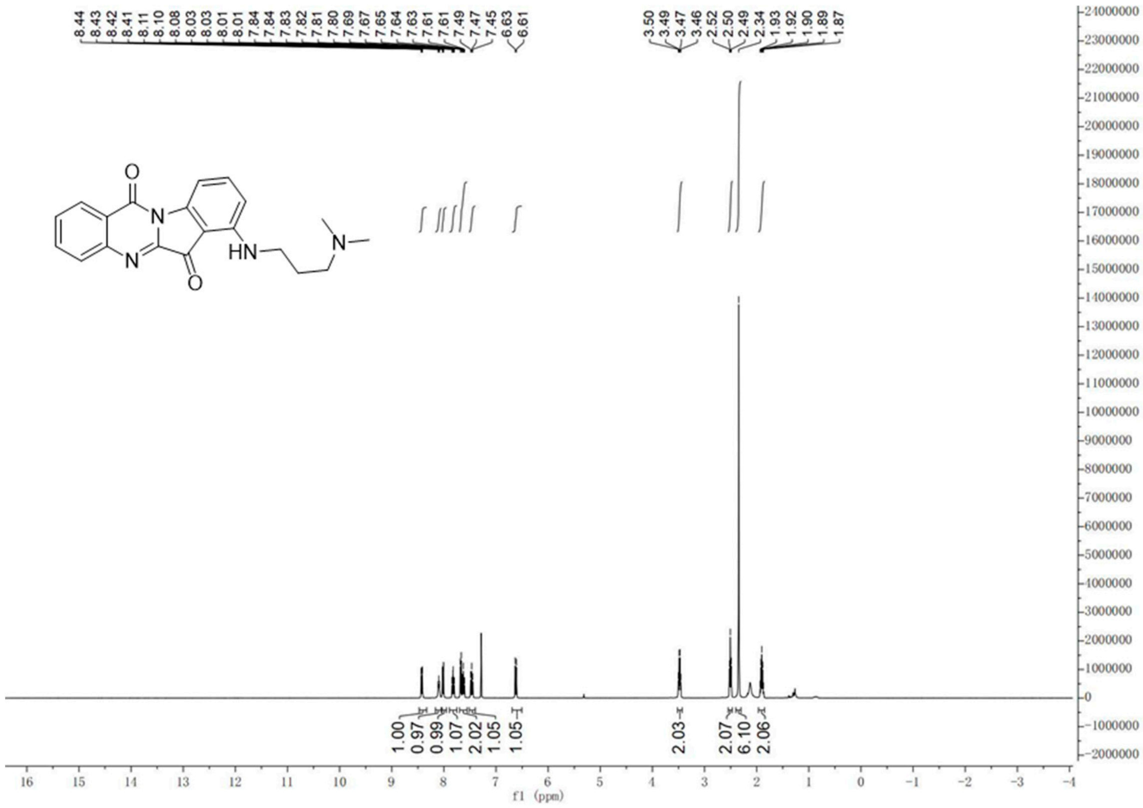

Figure S4. ^{1}H NMR Spectrum ($CDCl_3$, 400 MHz) of 7b.

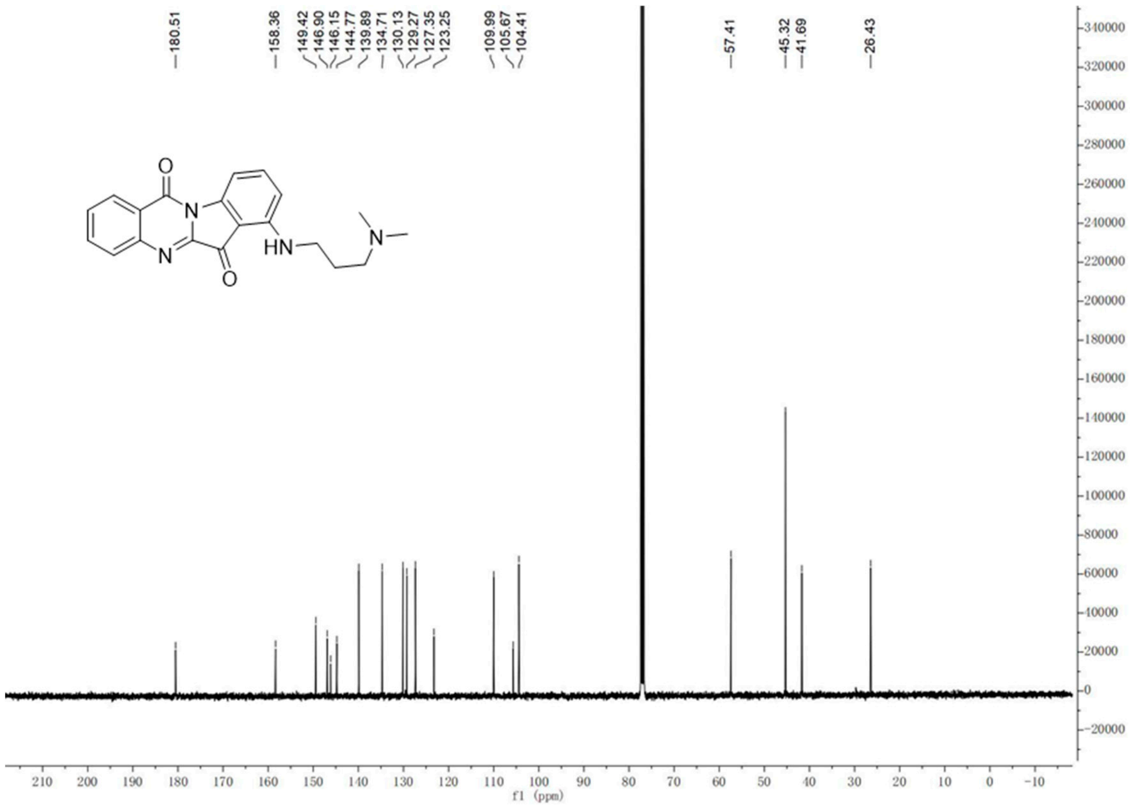

Figure S5. ^{13}**C NMR Spectrum (CDCl$_3$, 101 MHz) of 7b.**

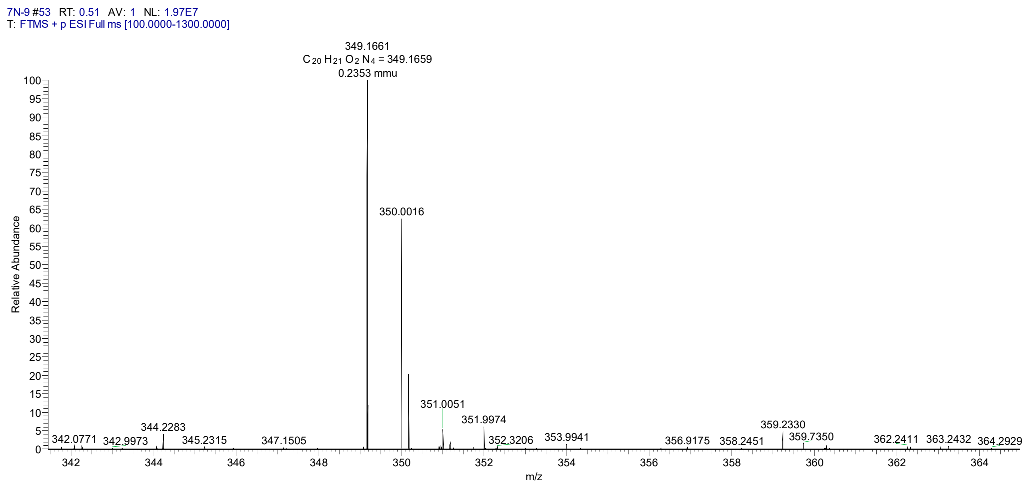

Figure S6. HR-MS Spectrum of 7b.

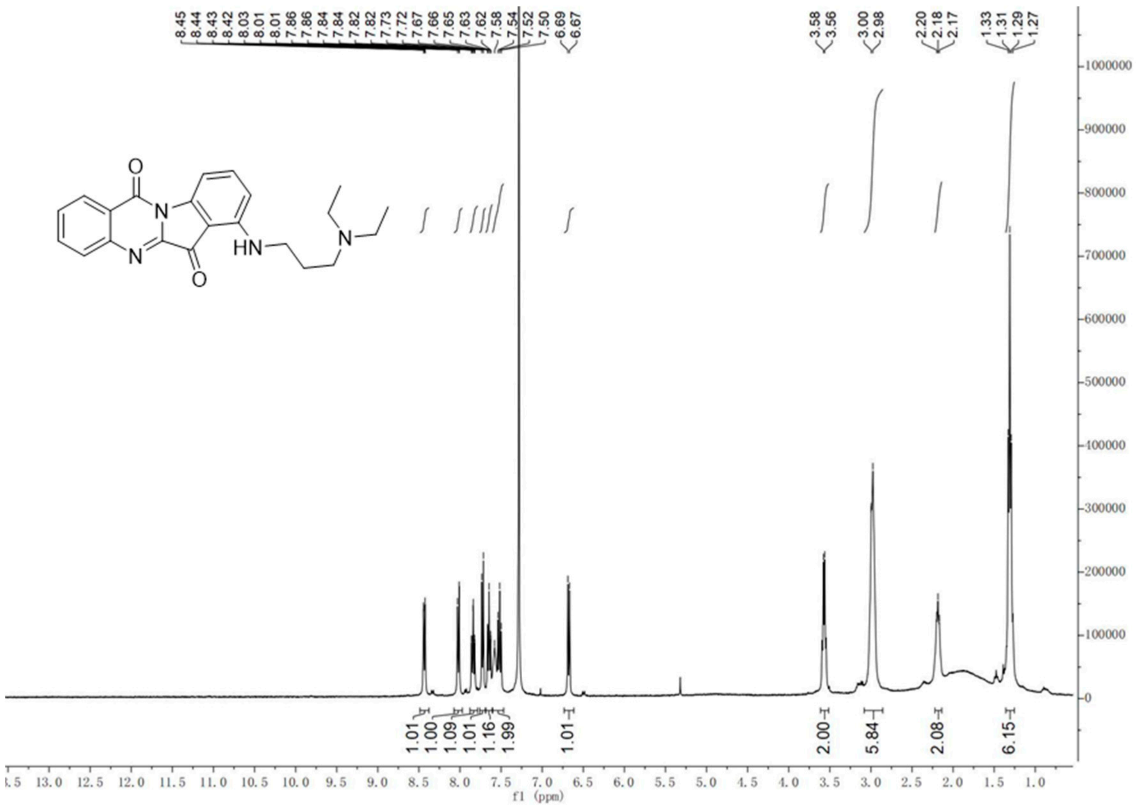

Figure S7. ^{1}H NMR Spectrum ($CDCl_3$, 400 MHz) of 7c.

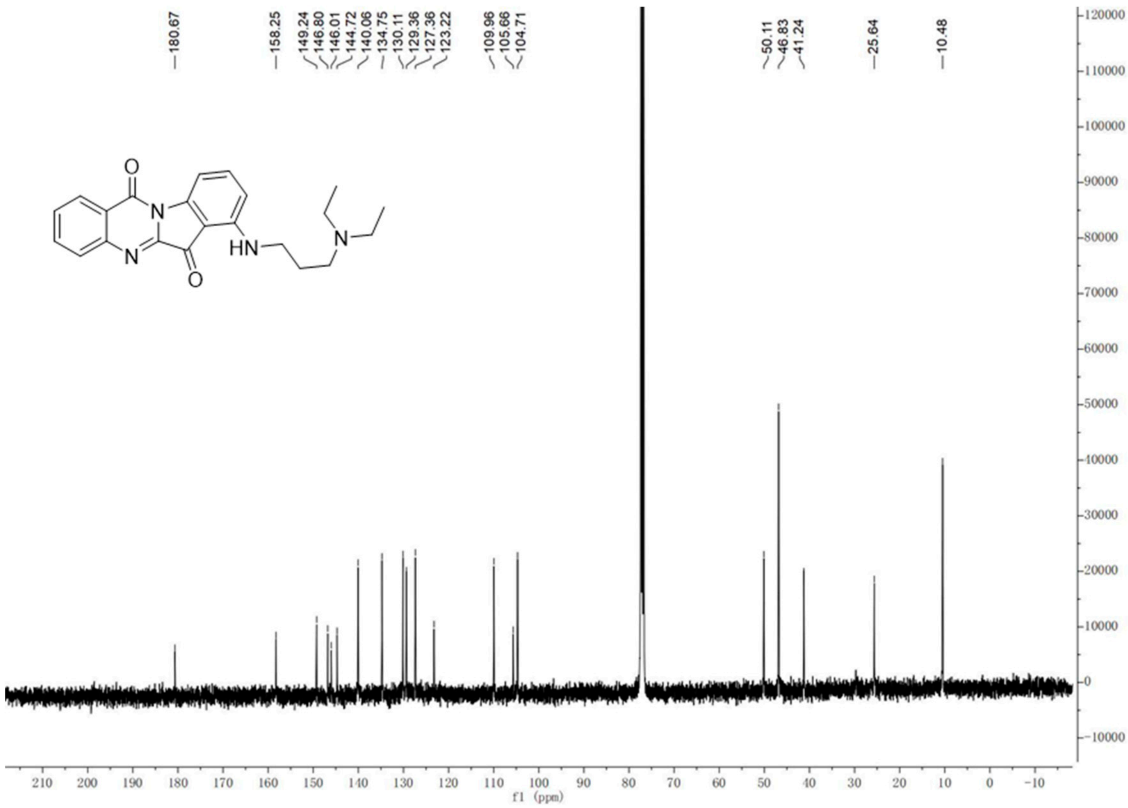

Figure S8. ^{13}C NMR Spectrum ($CDCl_3$, 101 MHz) of 7c.

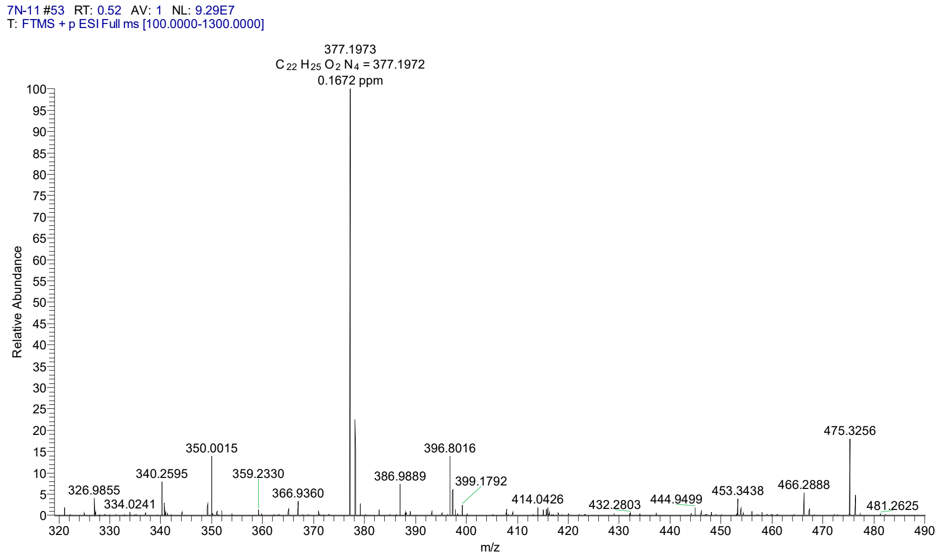

Figure S9. HR-MS Spectrum of 7c.

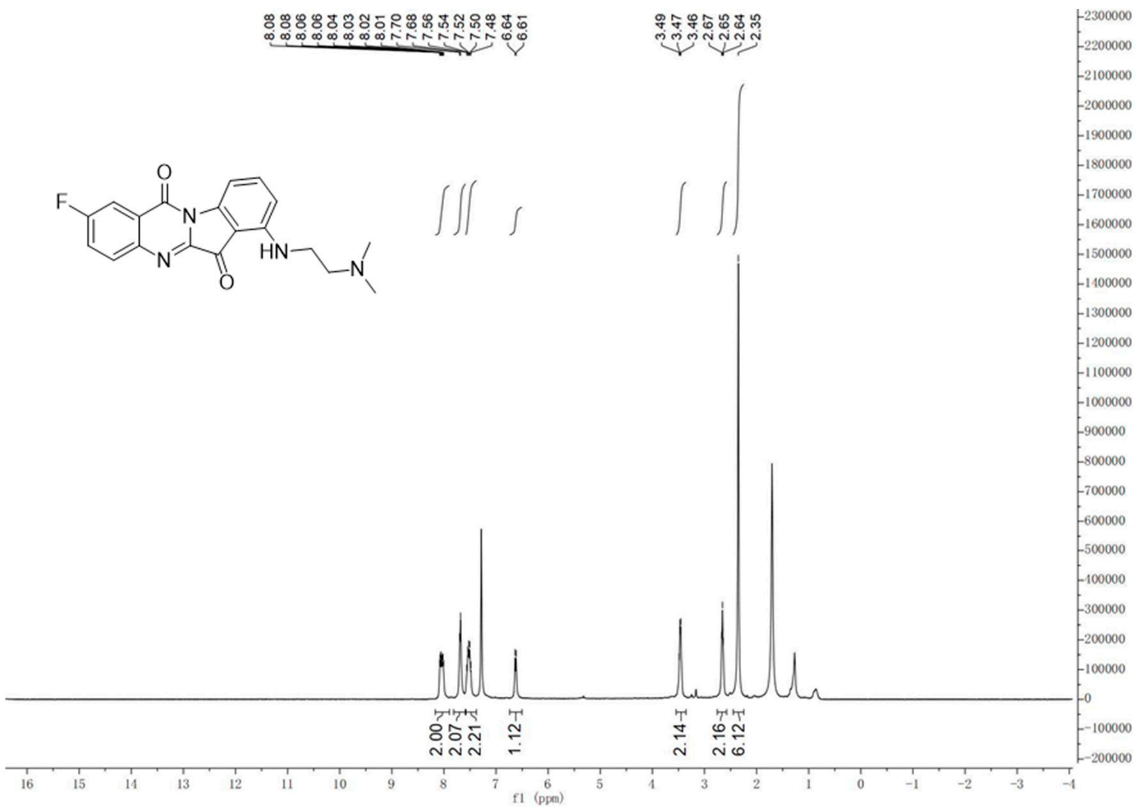

Figure S10. ^{1}H NMR Spectrum ($CDCl_3$, 400 MHz) of 7d.

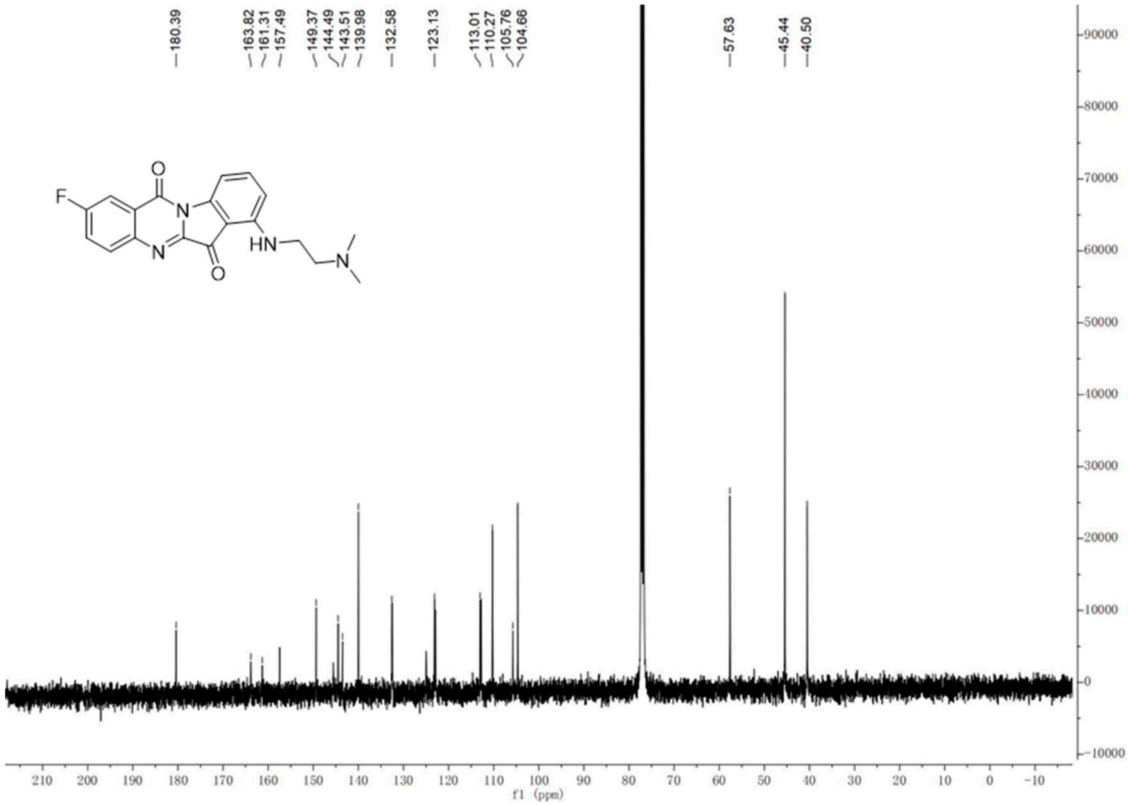

Figure S11. ^{13}C NMR Spectrum ($CDCl_3$, 101 MHz) of 7d.

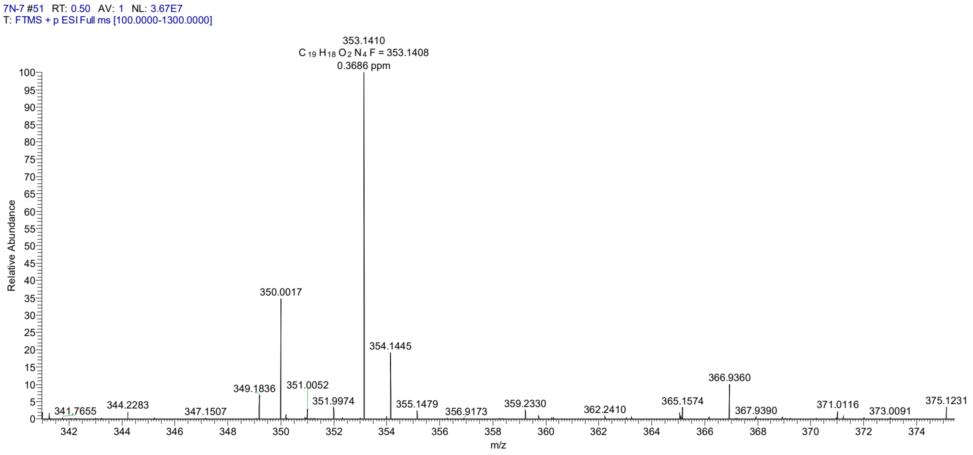

Figure S12. HR-MS Spectrum of 7d.

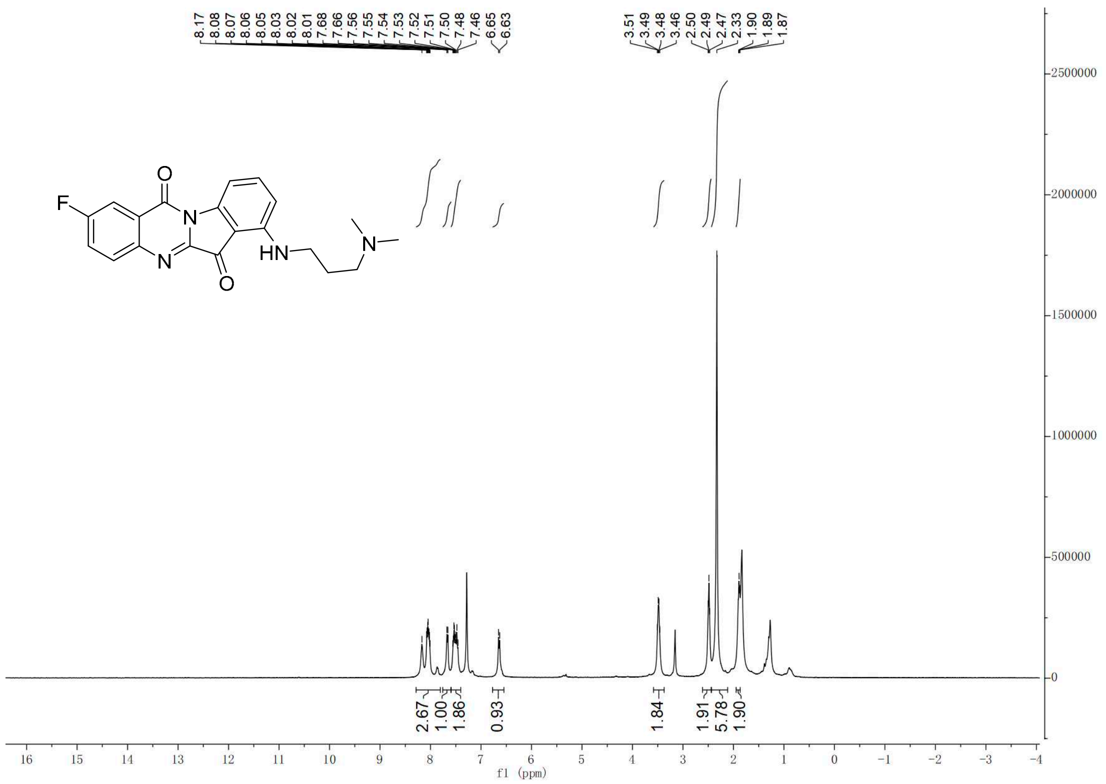

Figure S13. ^{1}H NMR Spectrum ($CDCl_3$, 400 MHz) of 7e.

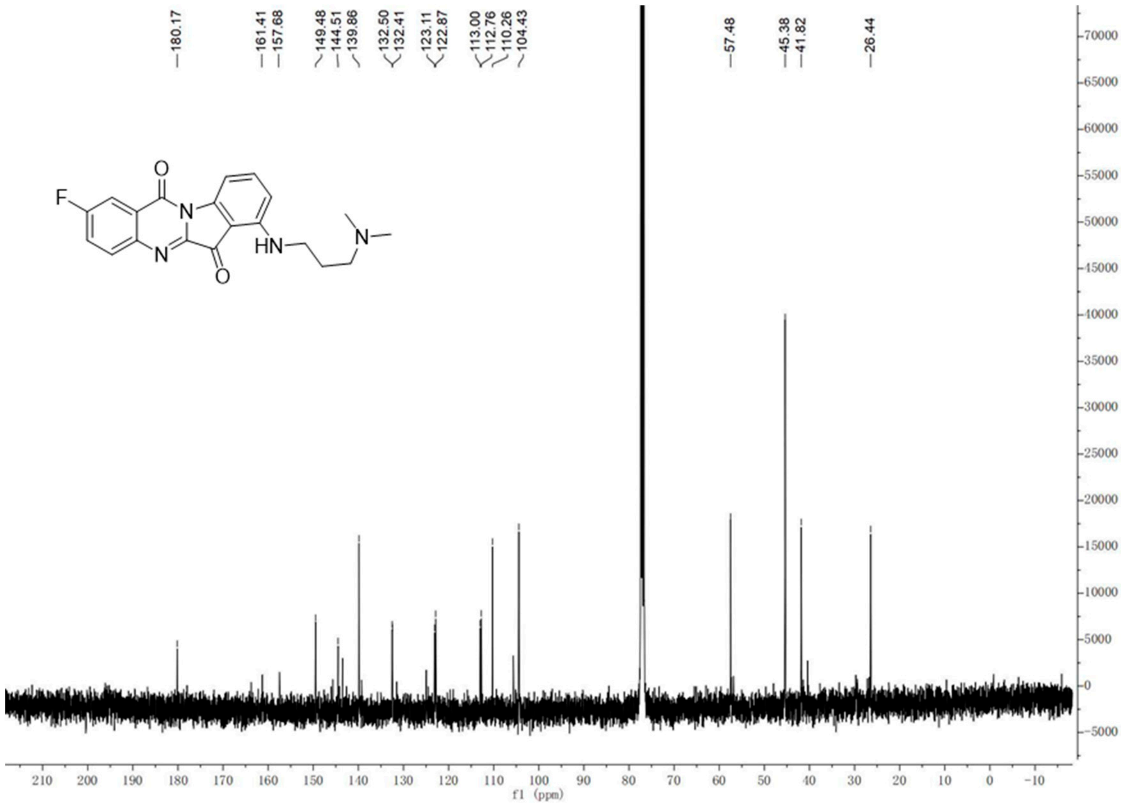

Figure S14. ^{13}C NMR Spectrum ($CDCl_3$, 101 MHz) of 7e.

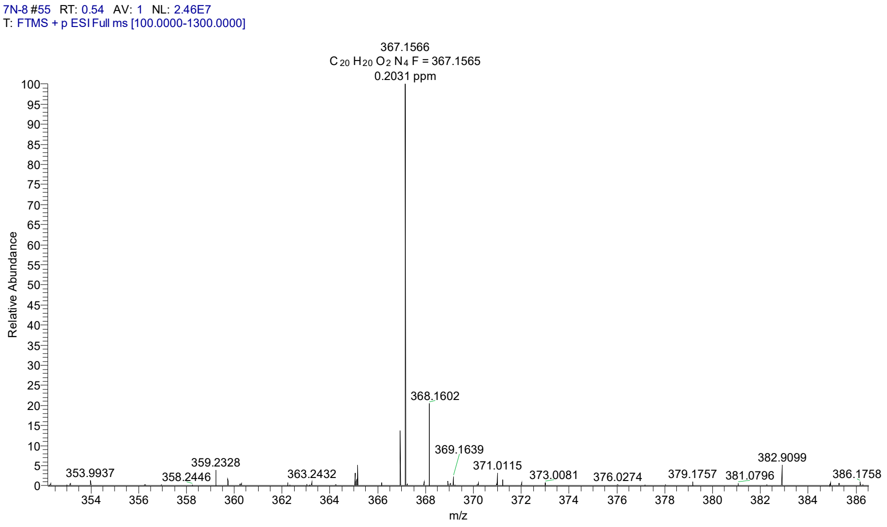

Figure S15. HR-MS Spectrum of 7e.

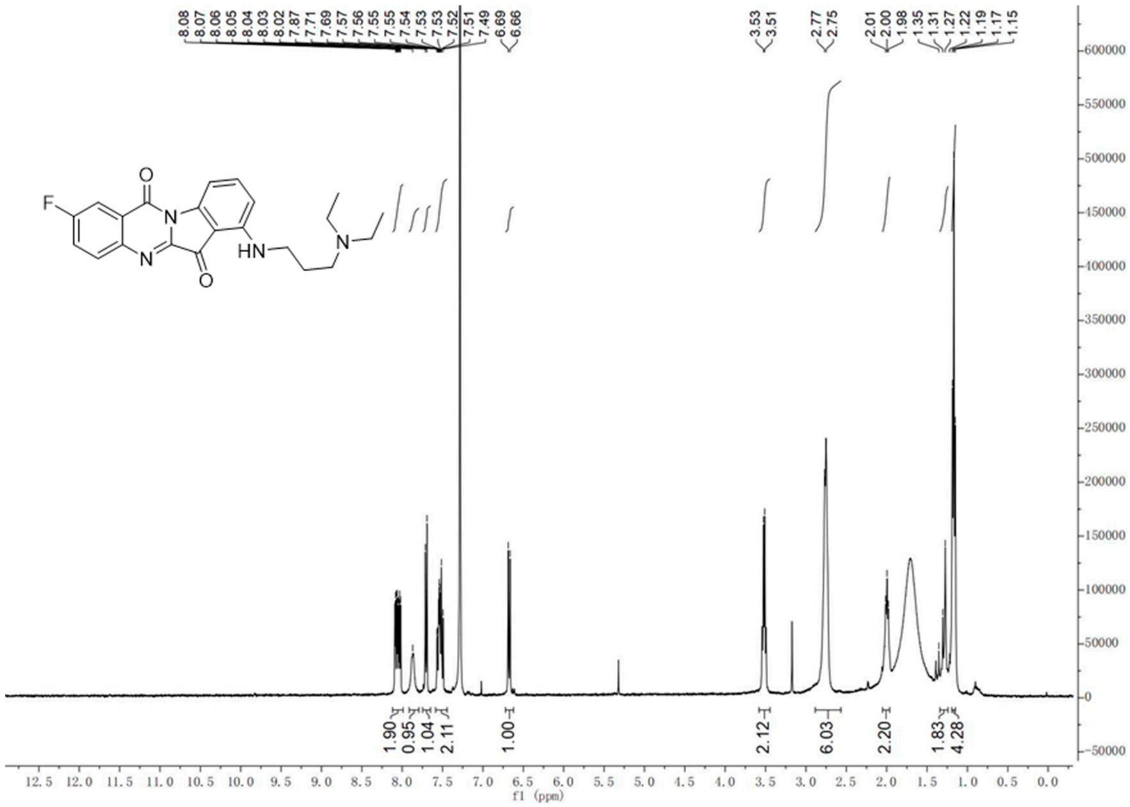

Figure S16. ^{1}H NMR Spectrum ($CDCl_3$, 400 MHz) of 7f.

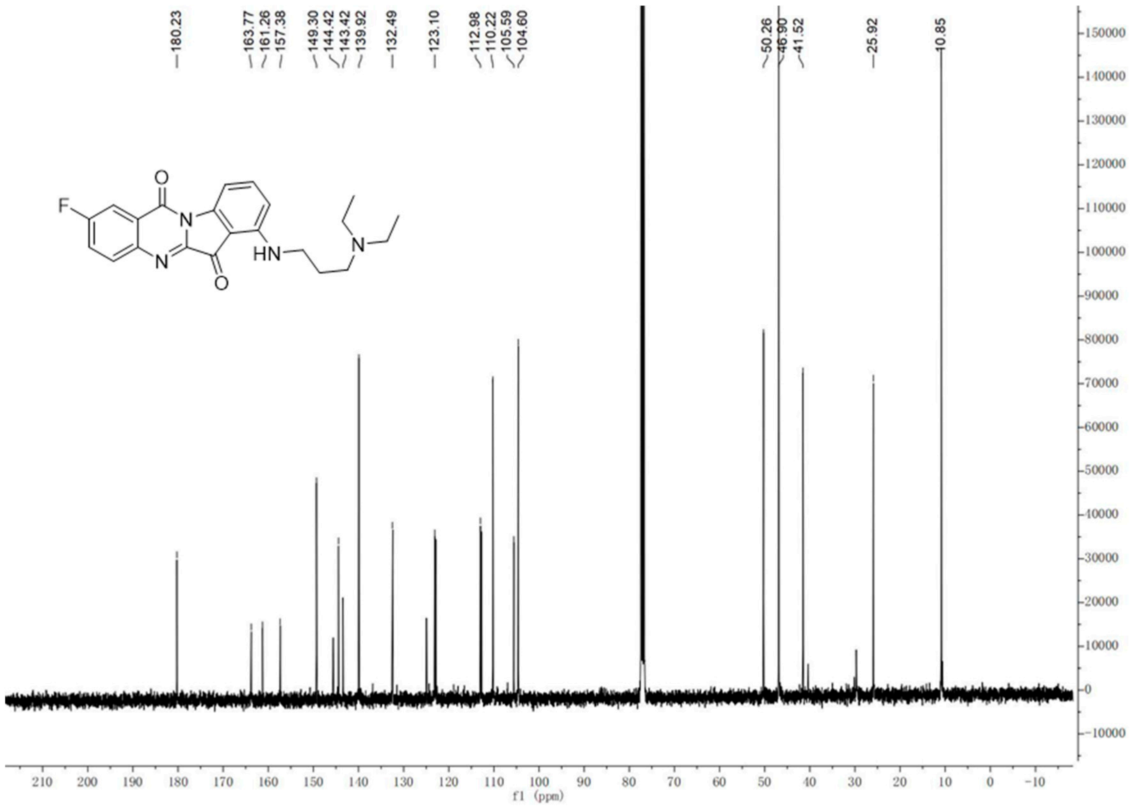

Figure S17. ^{13}**C NMR Spectrum (CDCl$_3$, 101 MHz) of 7f.**

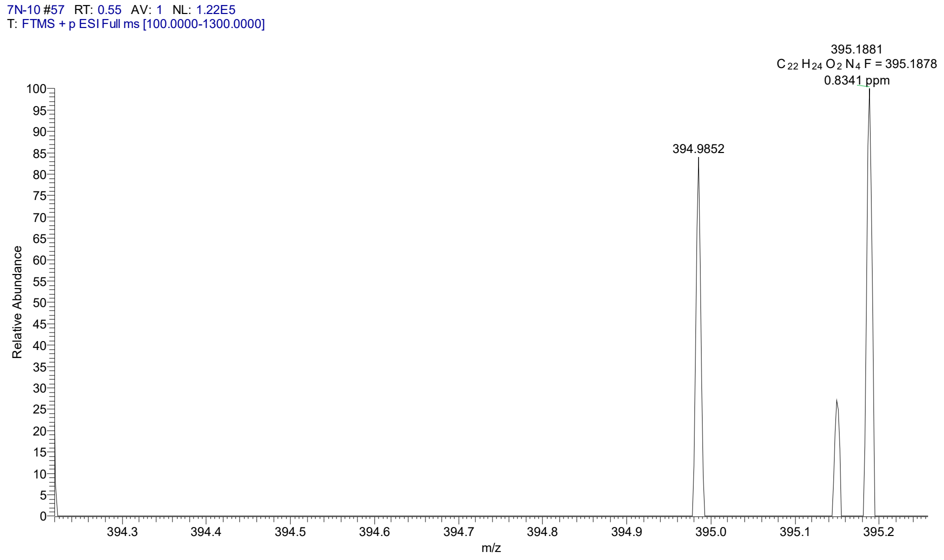

Figure S18. HR-MS Spectrum of 7f.

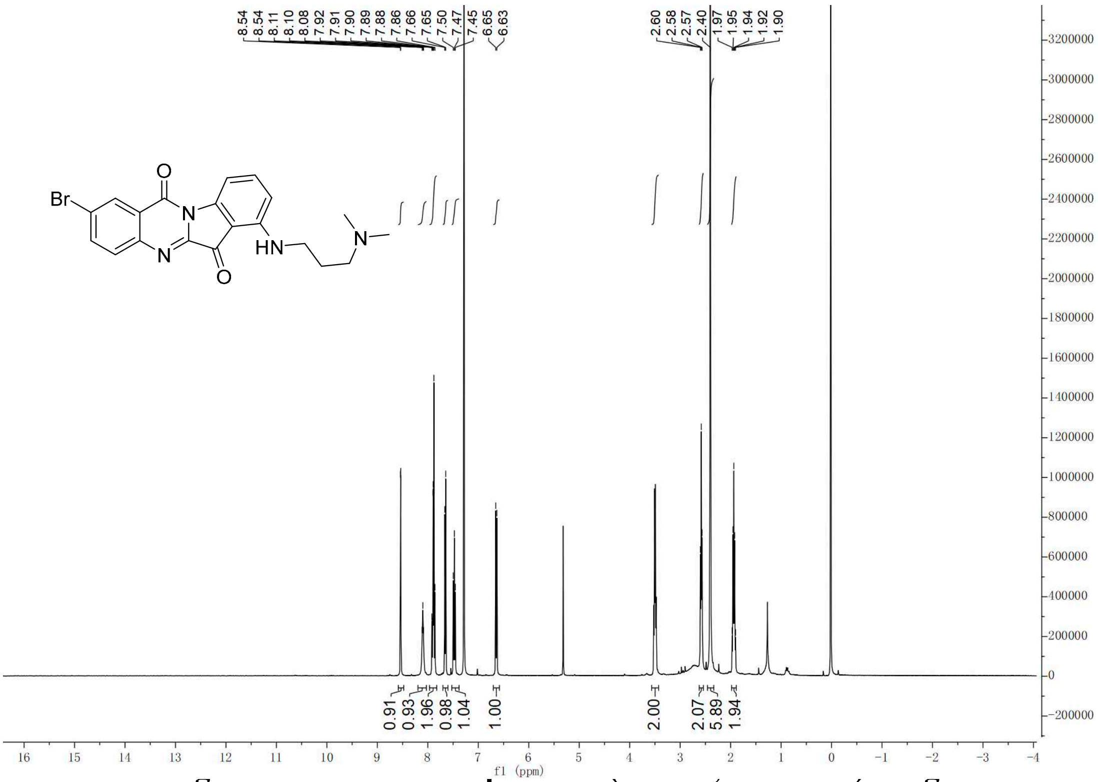

Figure S19. ^{1}H NMR Spectrum ($CDCl_3$, 400 MHz) of 7g.

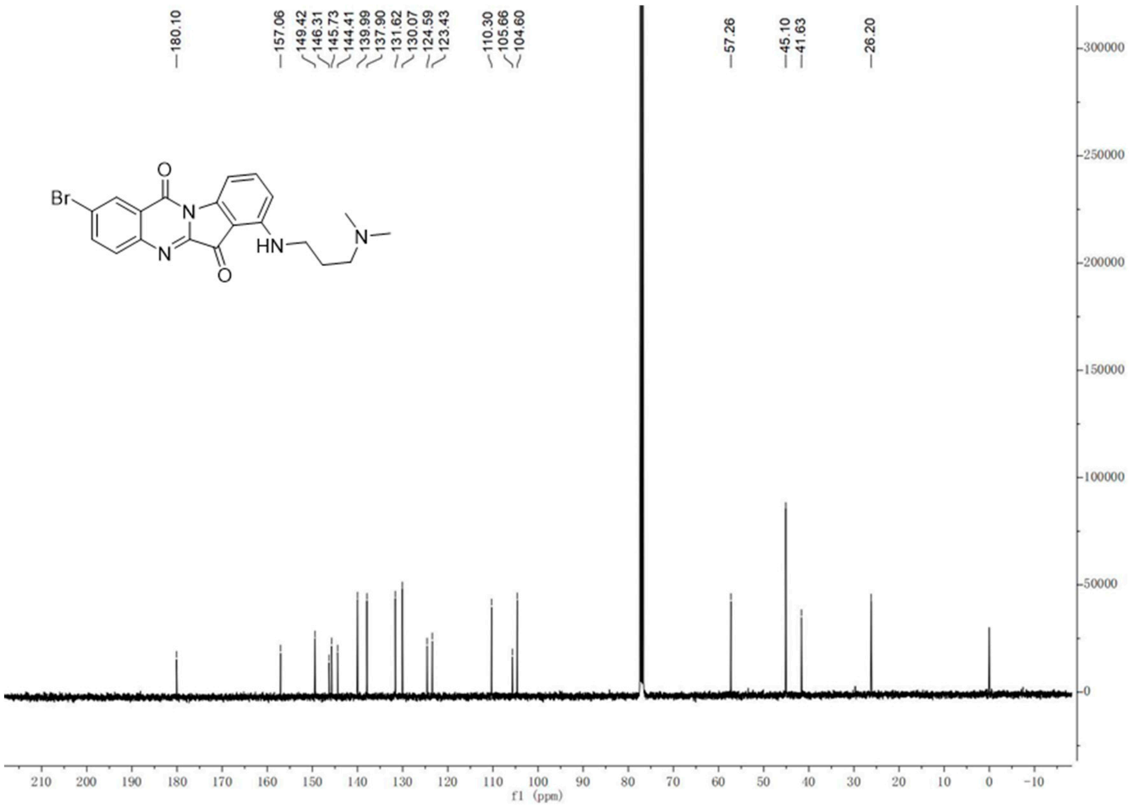

Figure S20. ^{13}C NMR Spectrum ($CDCl_3$, 101 MHz) of 7g.

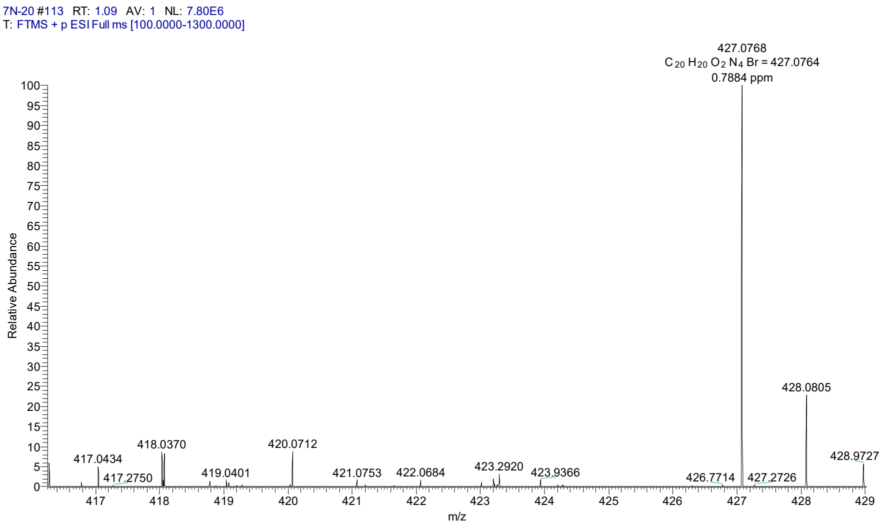

Figure S21. HR-MS Spectrum of 7g.

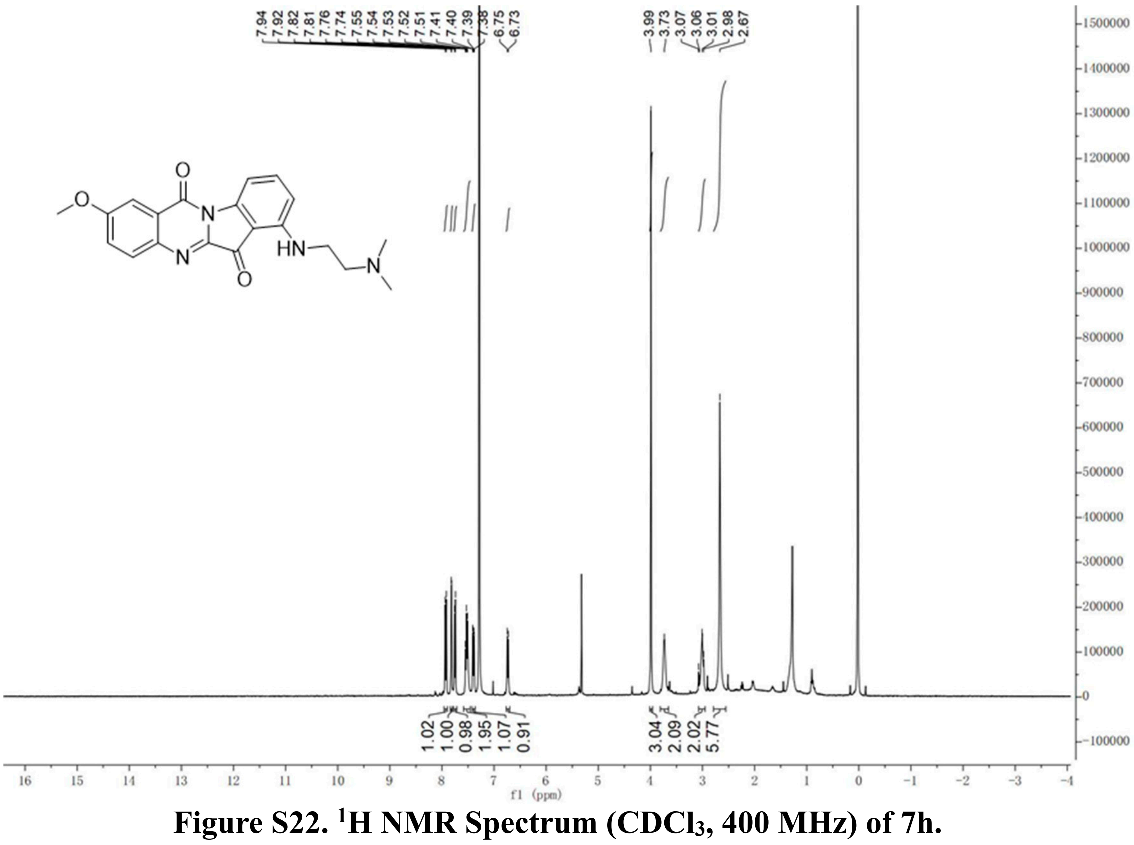

Figure S22. ^{1}H NMR Spectrum ($CDCl_3$, 400 MHz) of 7h.

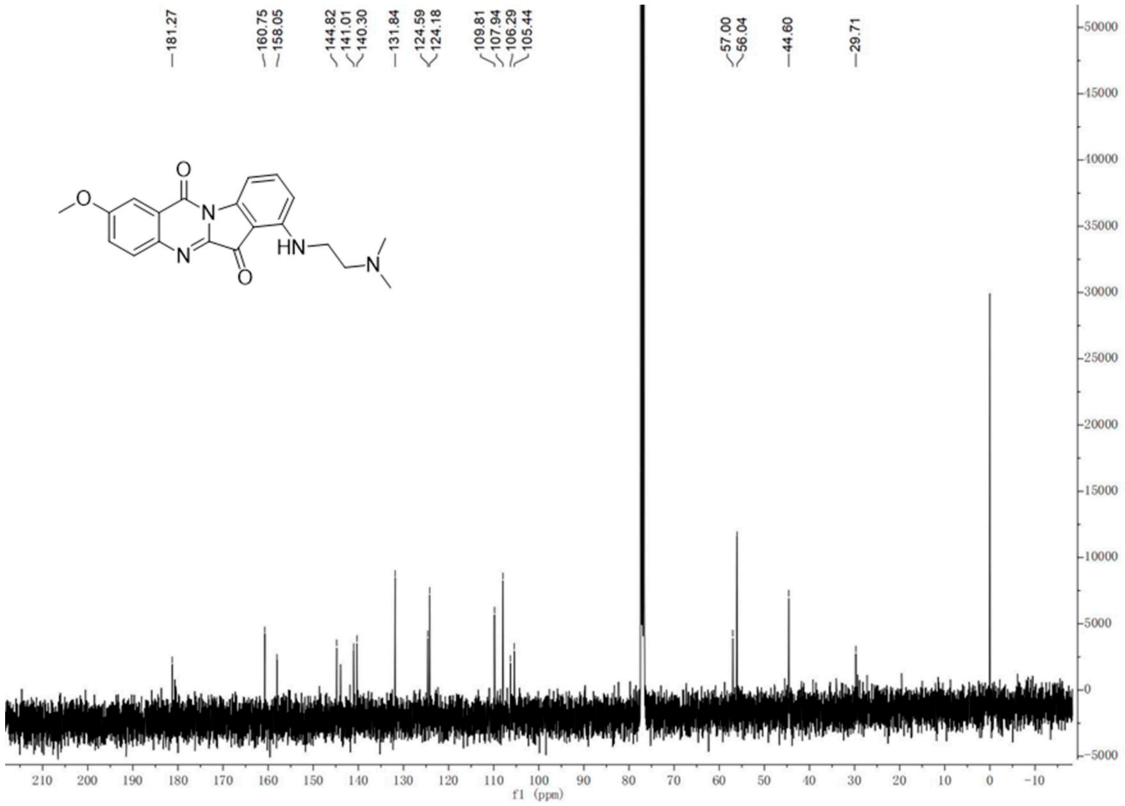

Figure S23. ^{13}**C NMR Spectrum (CDCl$_3$, 101 MHz) of 7h.**

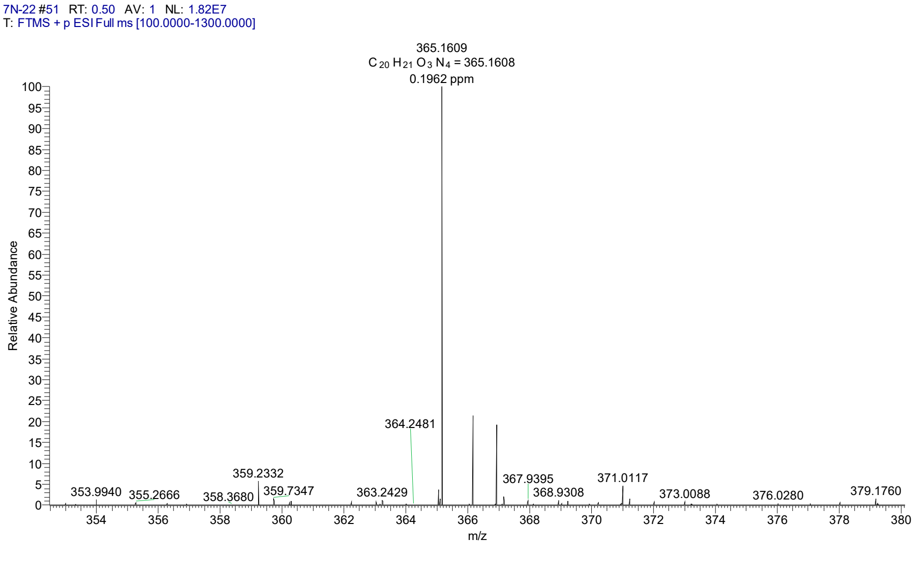

Figure S24. HR-MS Spectrum of 7h.

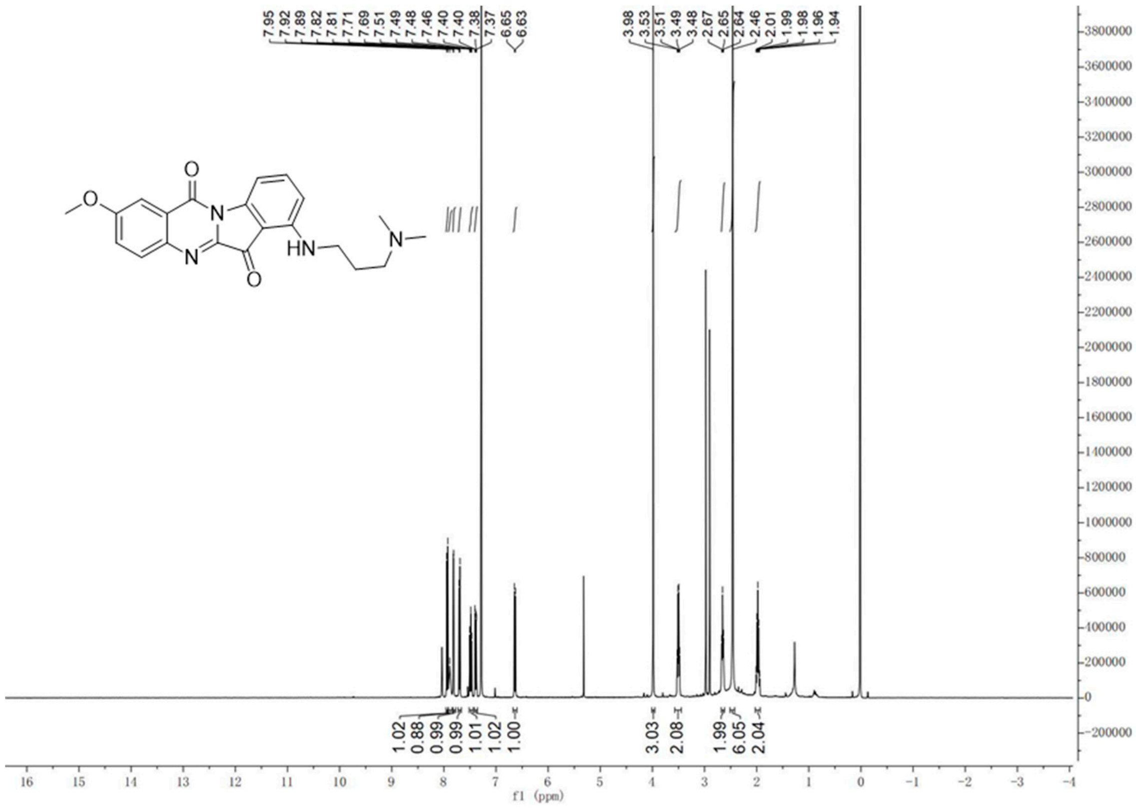

Figure S25. ^{1}H NMR Spectrum ($CDCl_3$, 400 MHz) of 7i.

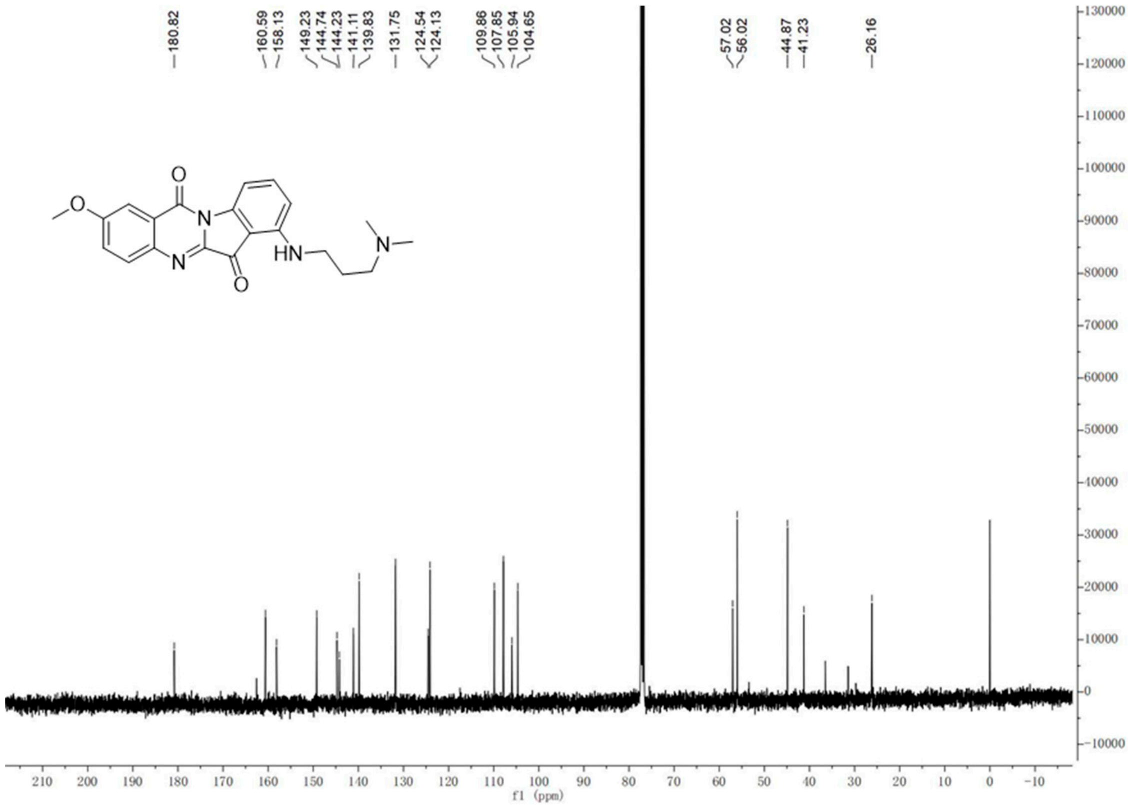

Figure S26. ^{13}C NMR Spectrum ($CDCl_3$, 101 MHz) of 7i.

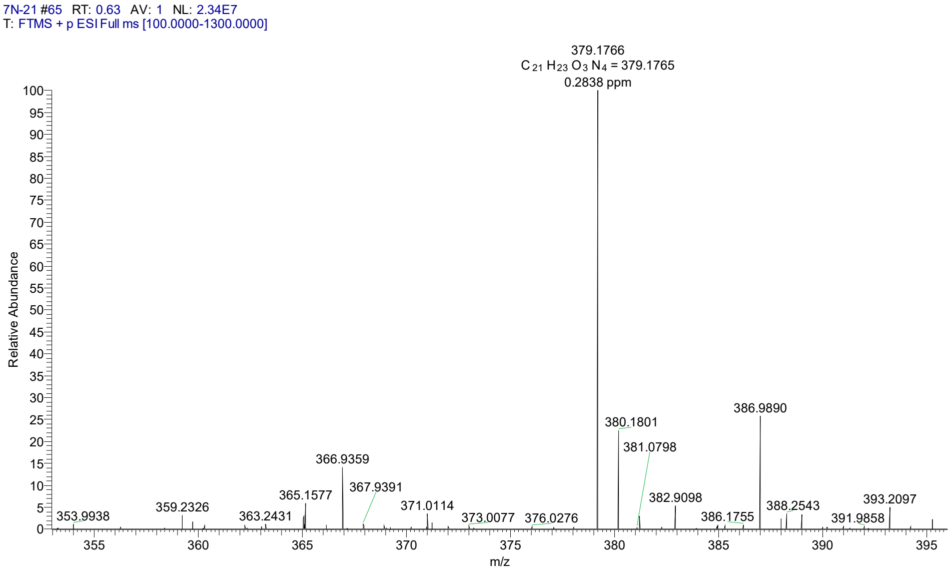

Figure S27. HR-MS Spectrum of 7i.

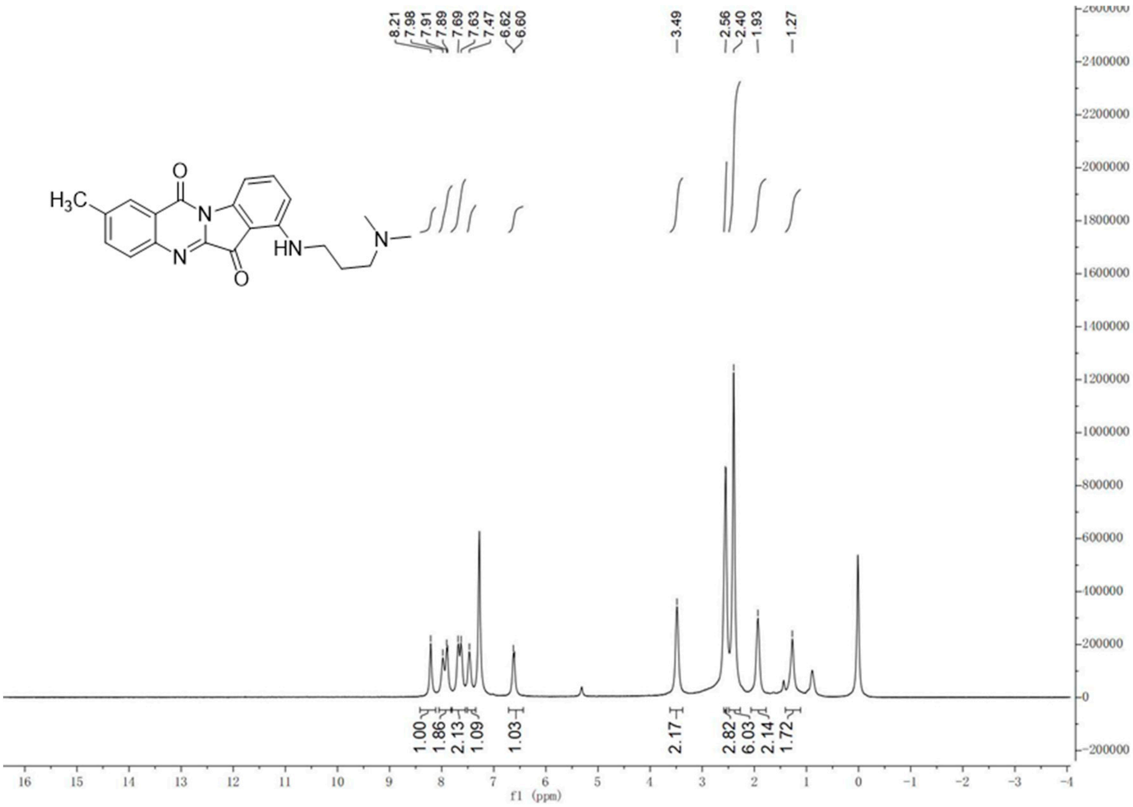

Figure S28. ^{1}H NMR Spectrum ($CDCl_3$, 400 MHz) of 7j.

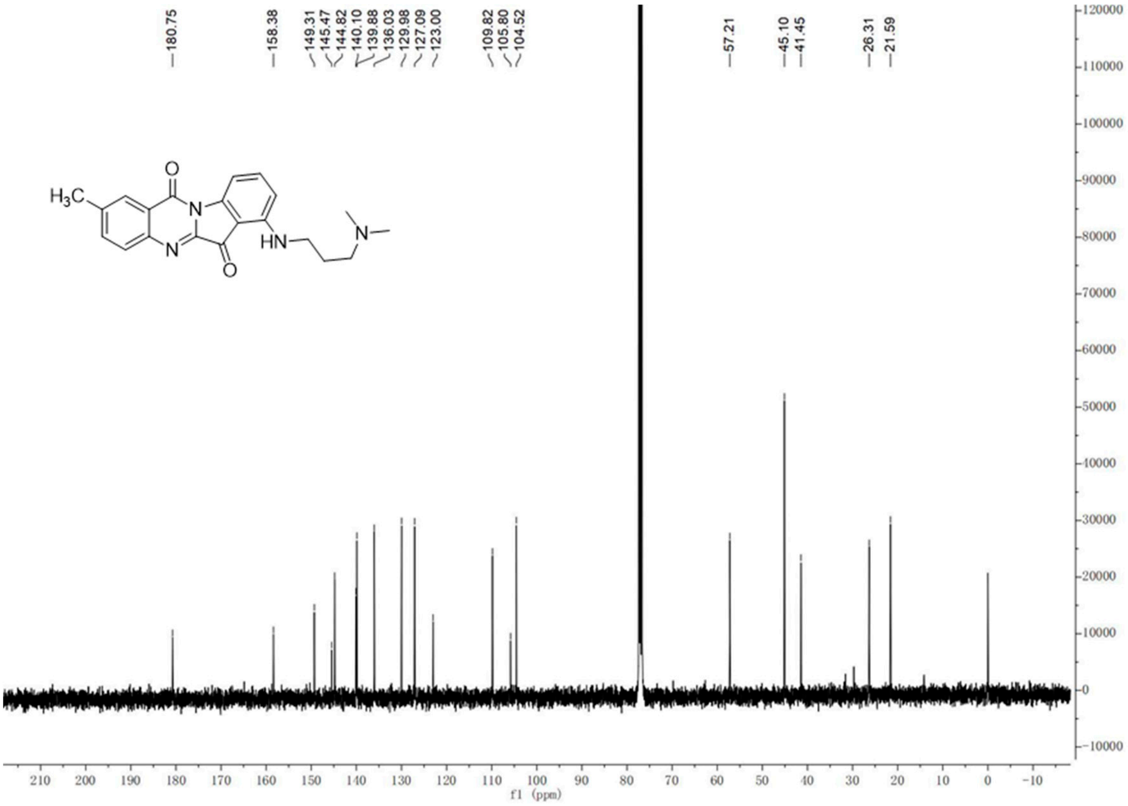

Figure S29. ^{13}C **NMR Spectrum** ($CDCl_3$, **101 MHz) of 7j.**

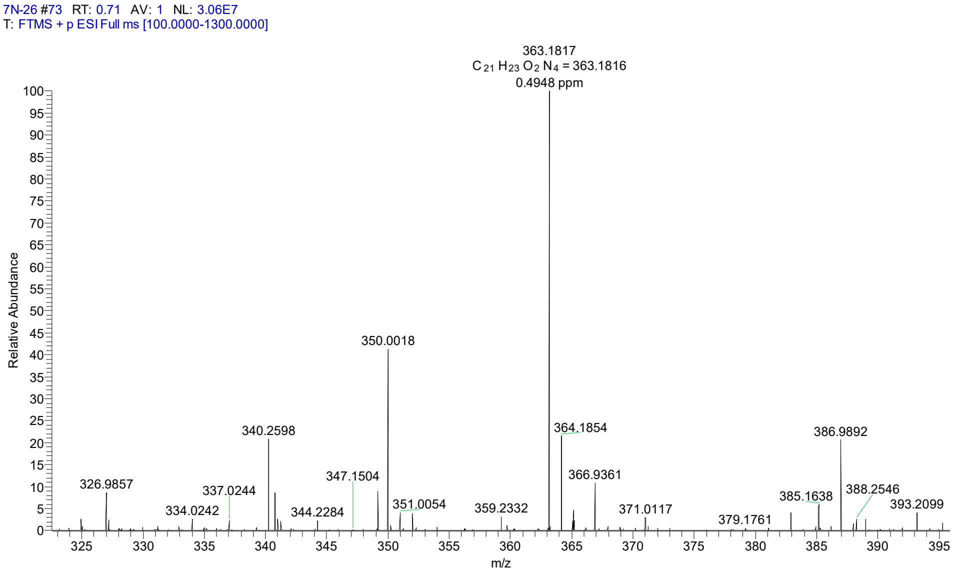

Figure S30. HR-MS Spectrum of 7j.

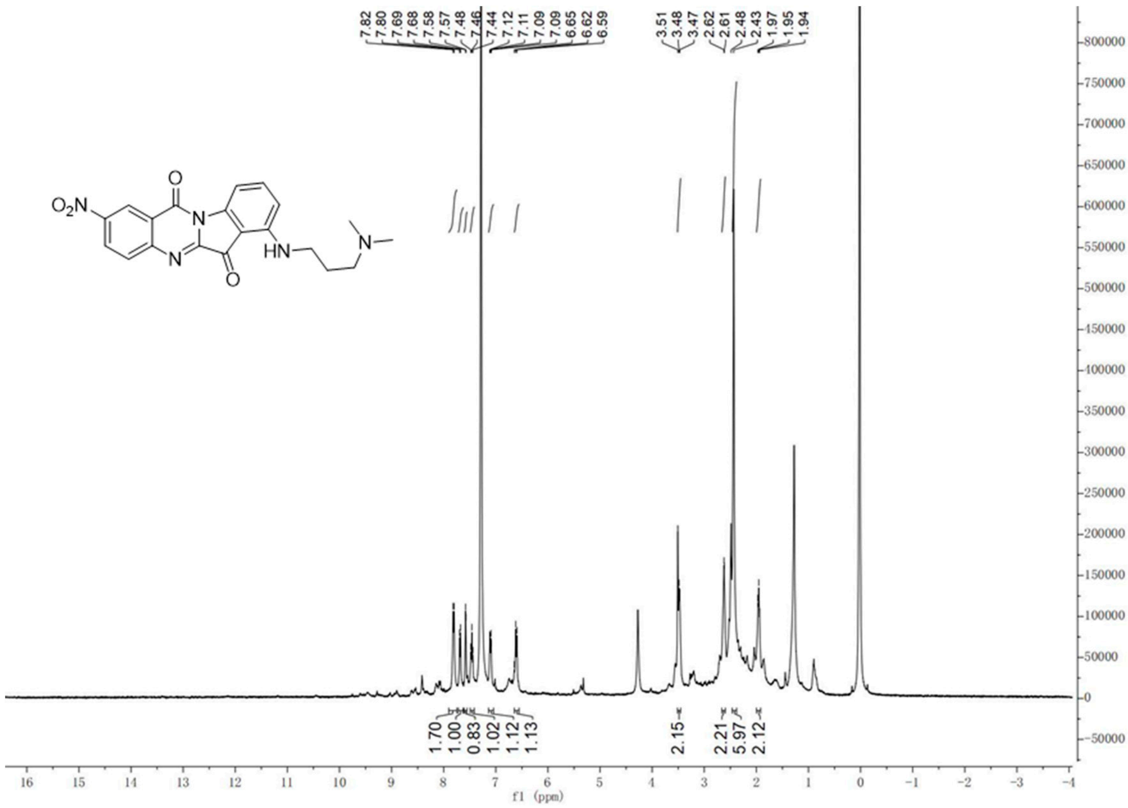

Figure S31. ^{1}H NMR Spectrum ($CDCl_3$, 400 MHz) of 7k.

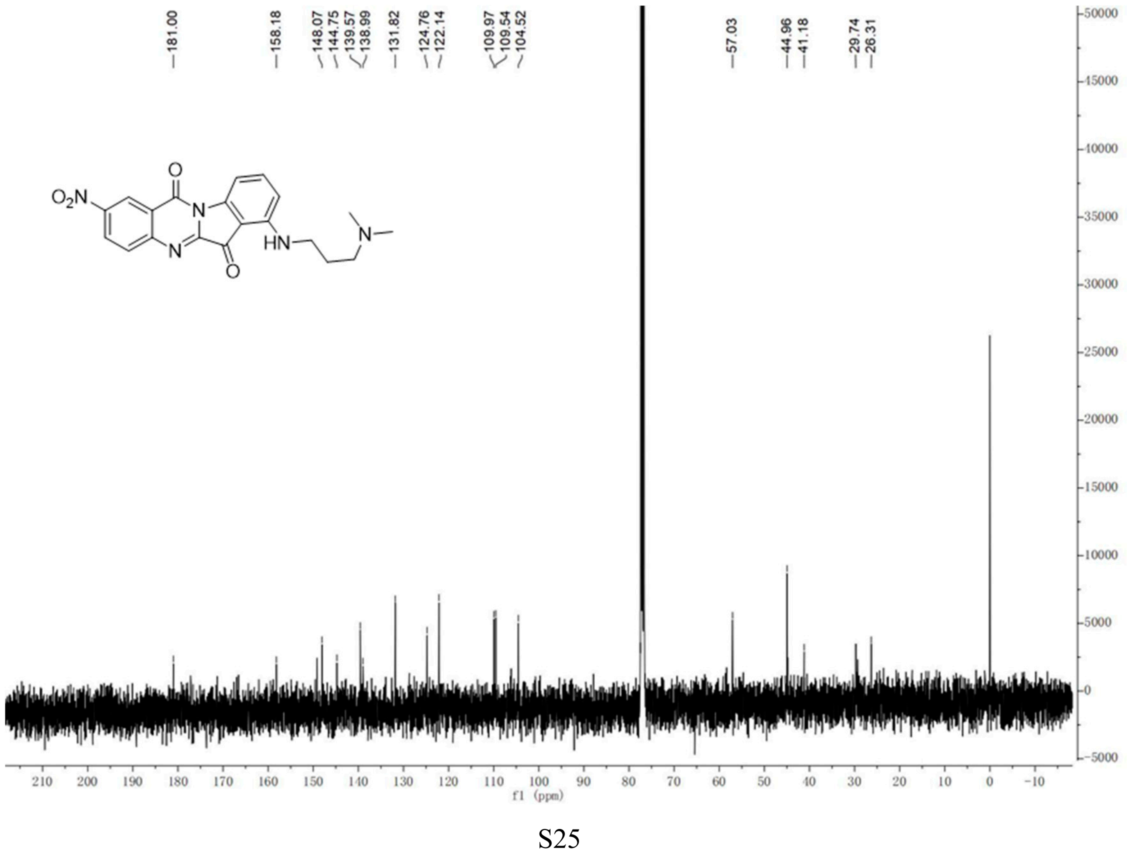

Figure S32. ^{13}C NMR Spectrum ($CDCl_3$, 101 MHz) of 7k.

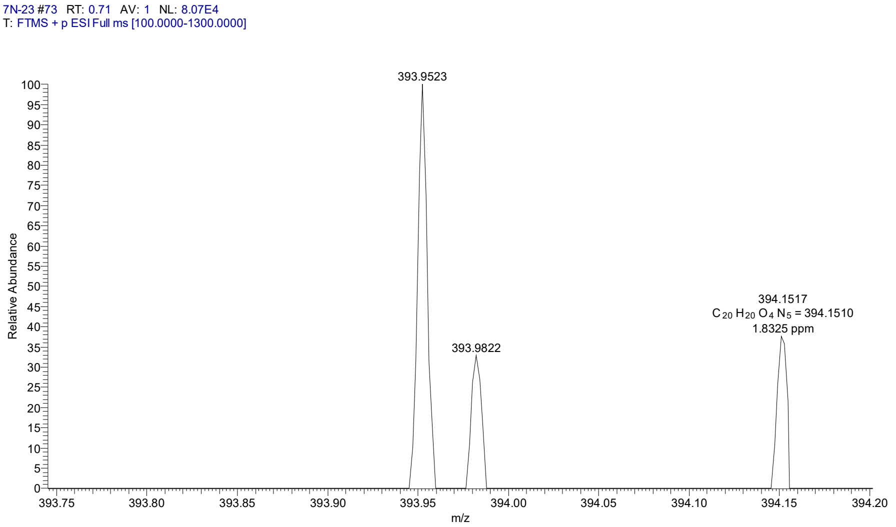

Figure S33. HR-MS Spectrum of 7k.

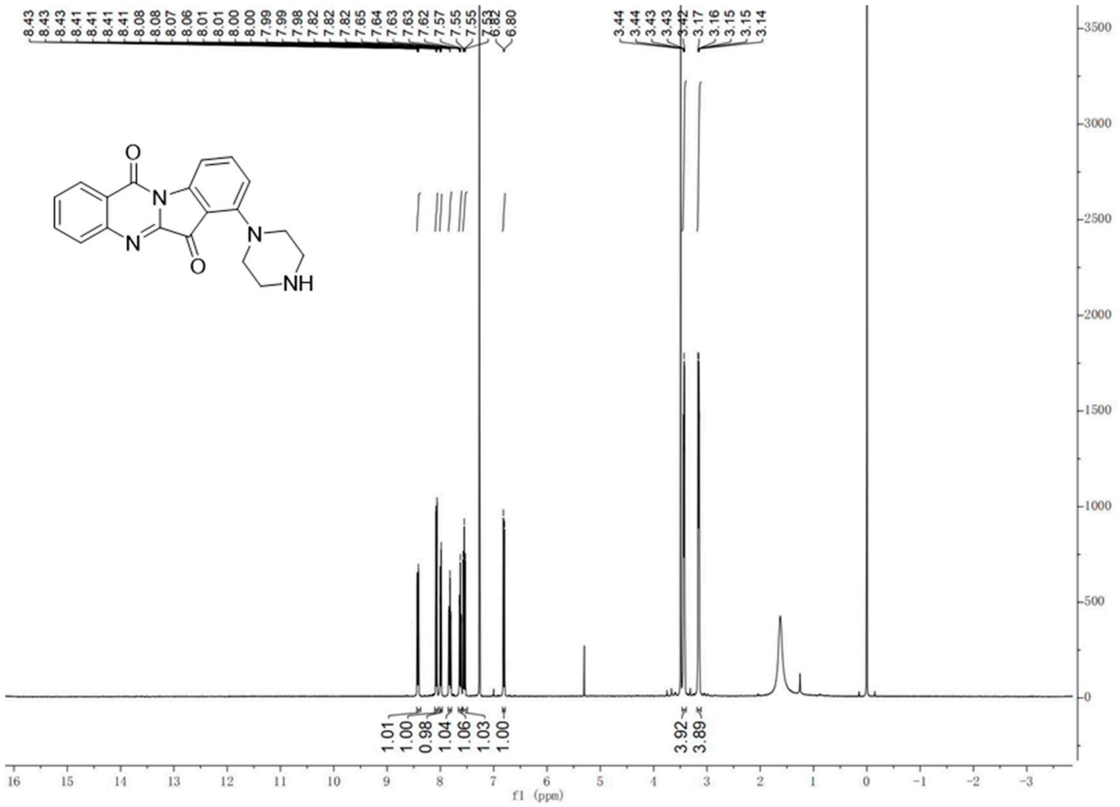

Figure S34. ^1H NMR Spectrum ($CDCl_3$, 400 MHz) of 7l.

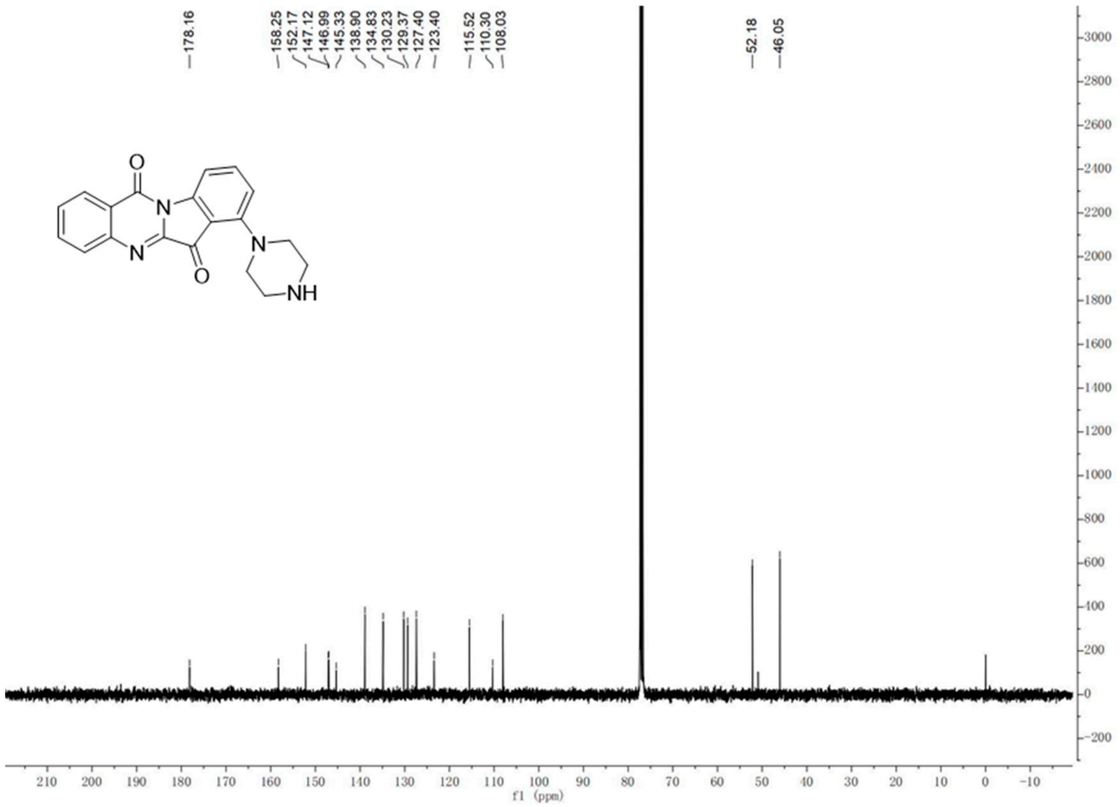

Figure S35. ^{13}C NMR Spectrum ($CDCl_3$, 101 MHz) of 7l.

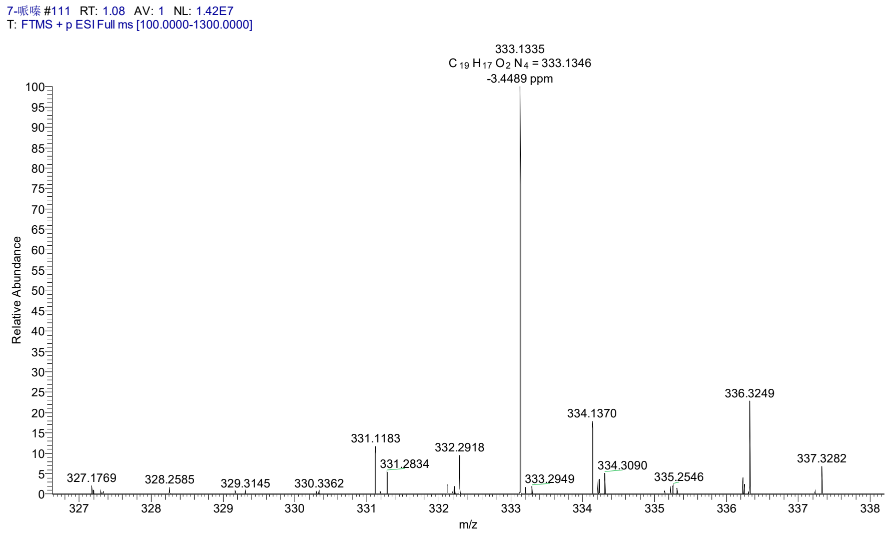

Figure S36. HR-MS Spectrum of 7l.

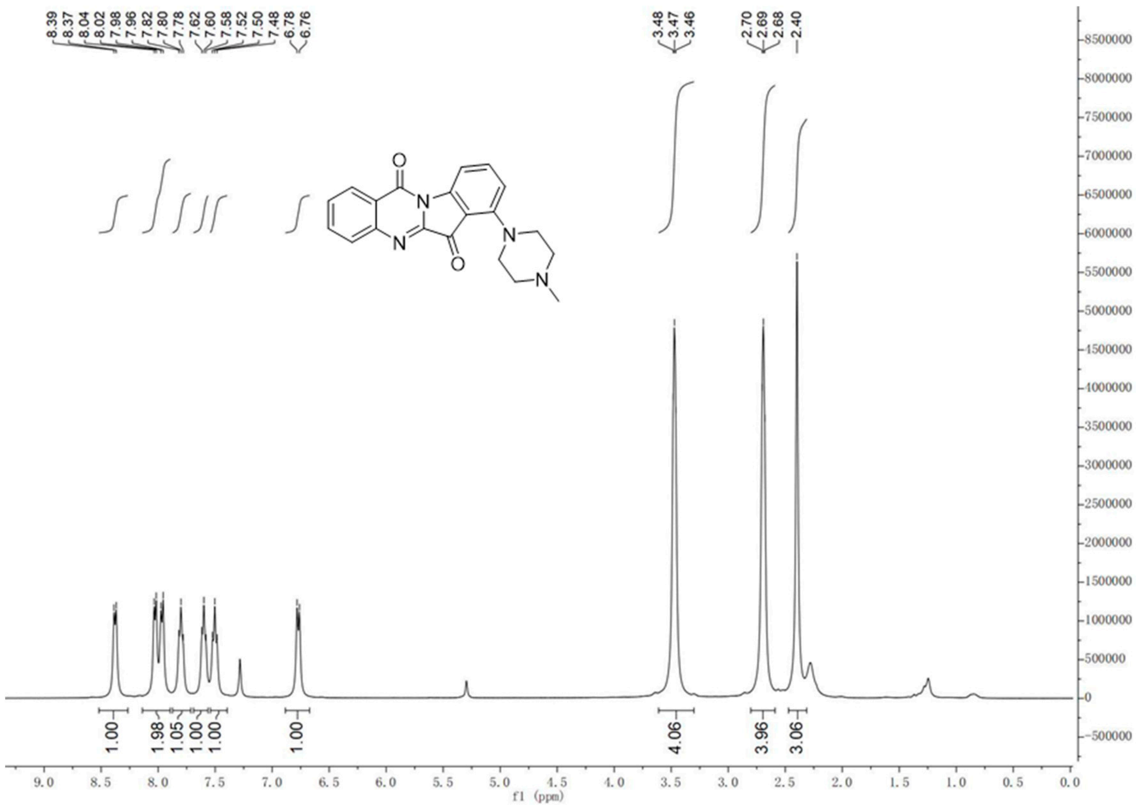

Figure S37. ^{1}H NMR Spectrum ($CDCl_3$, 400 MHz) of 7m.

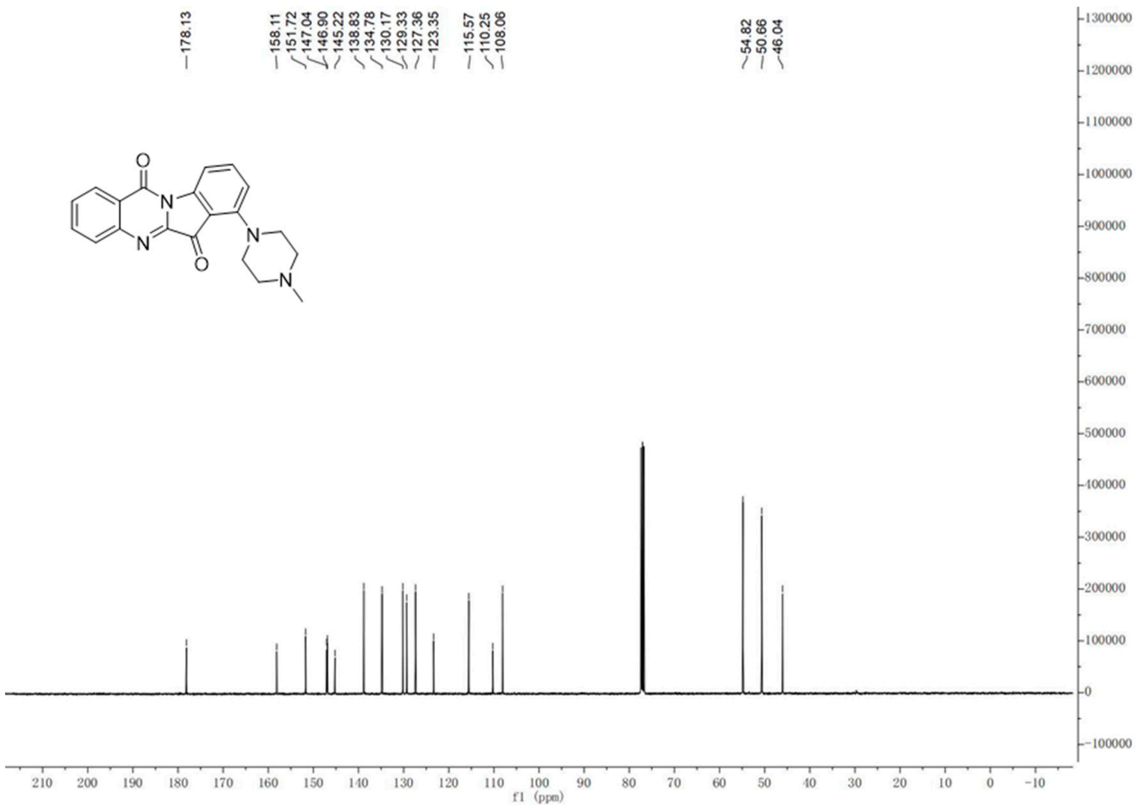

Figure S38. ^{13}C NMR Spectrum ($CDCl_3$, 101 MHz) of 7m.

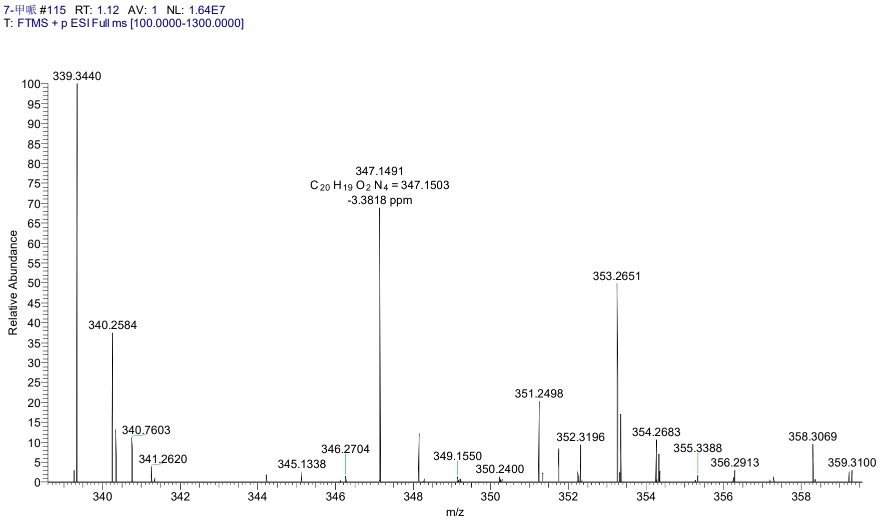

Figure S39. HR-MS Spectrum of 7m.

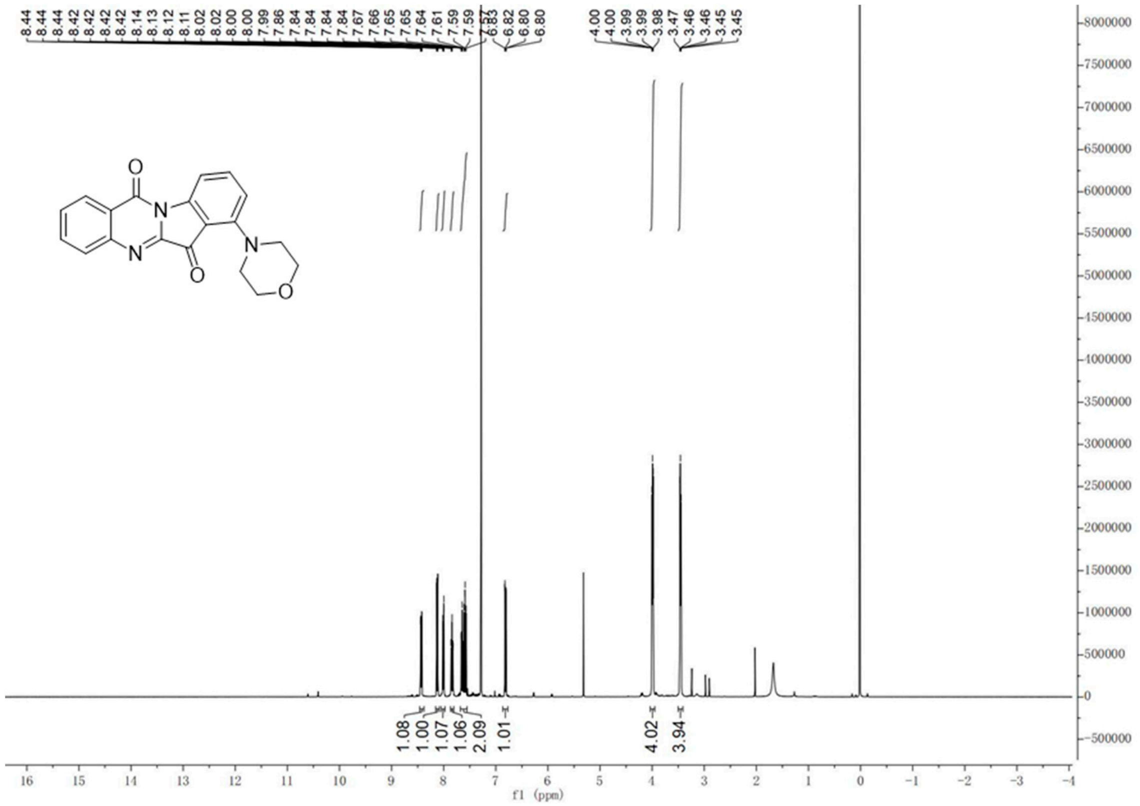

Figure S40. ^{1}H NMR Spectrum ($CDCl_3$, 400 MHz) of 7n.

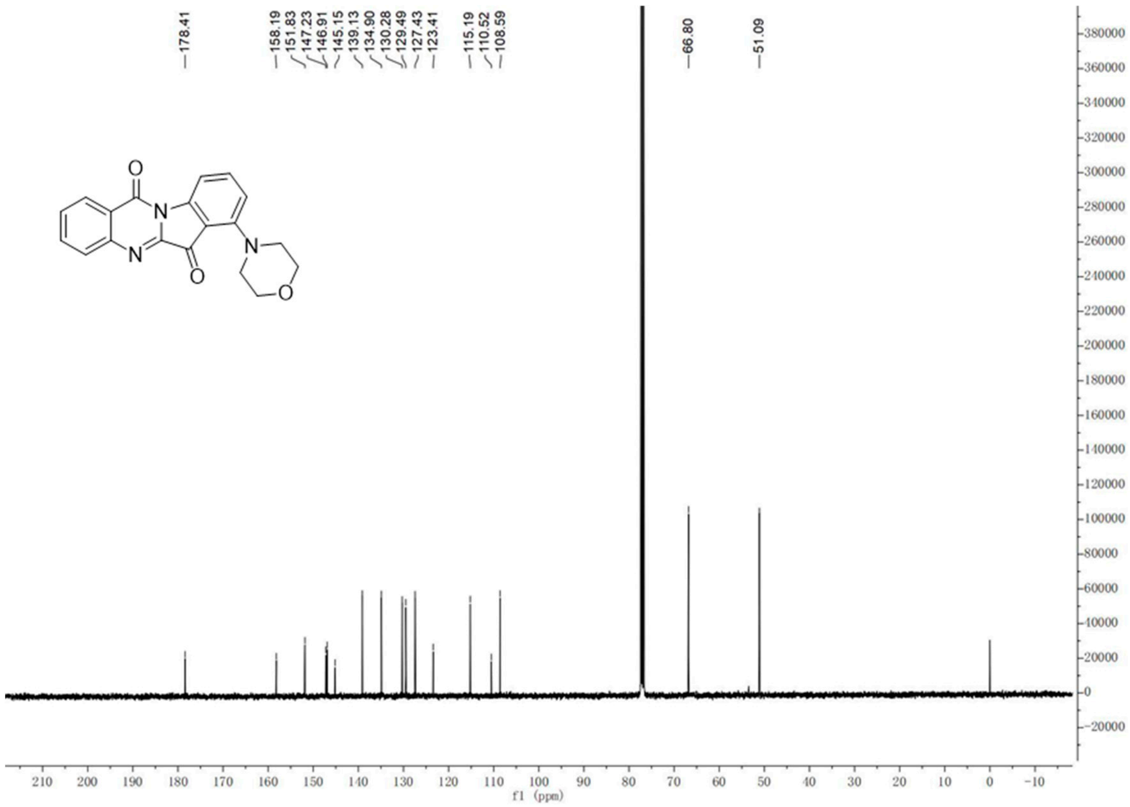

Figure S41. ^{13}C NMR Spectrum ($CDCl_3$, 101 MHz) of 7n.

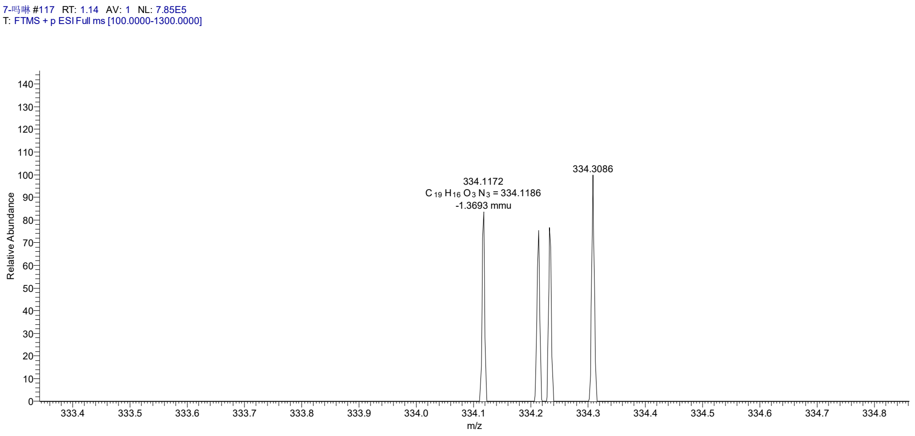

Figure S42. HR-MS Spectrum of 7n.

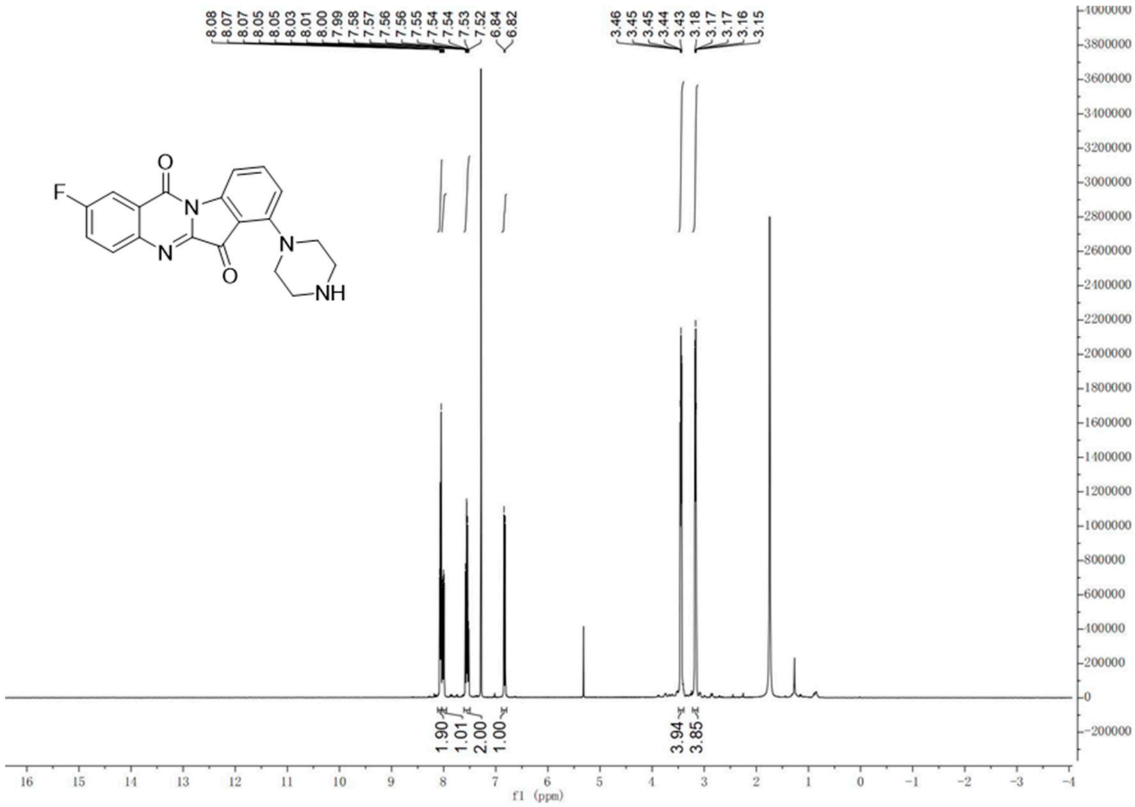

Figure S43. ^{1}H NMR Spectrum (CDCl$_3$, 400 MHz) of 7o.

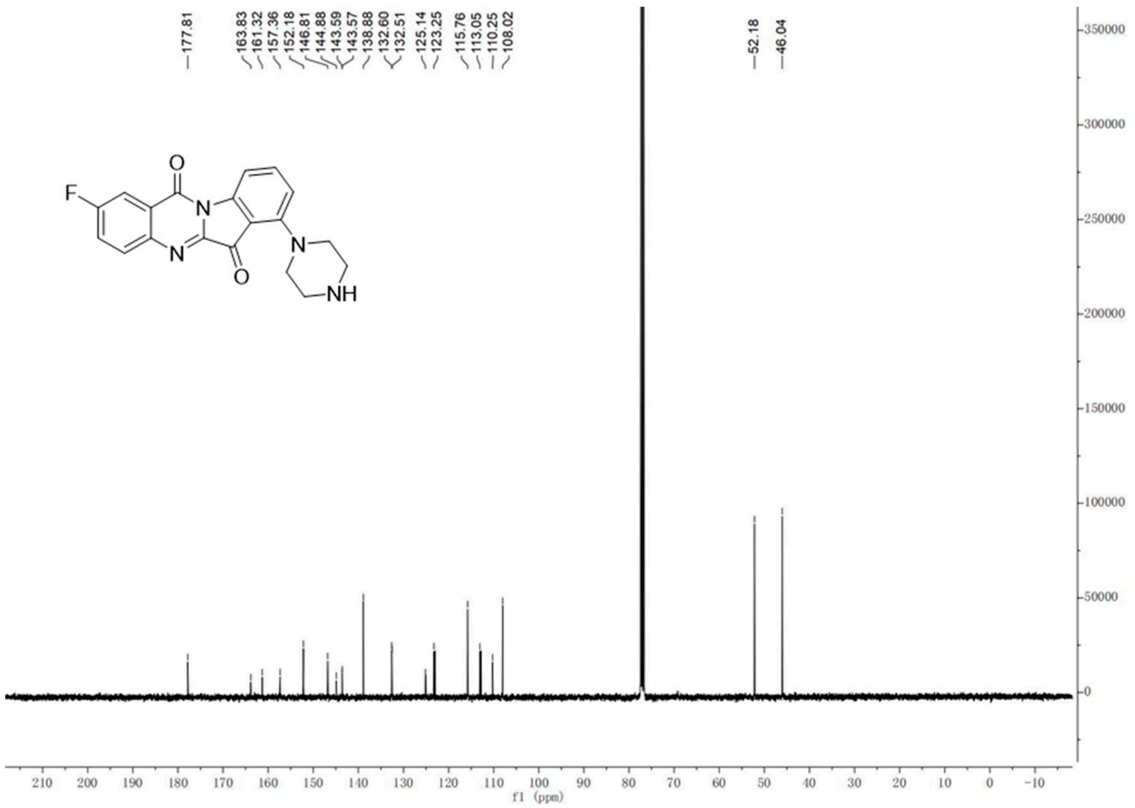

Figure S44. ^{13}C NMR Spectrum (CDCl$_3$, 101 MHz) of 7o.

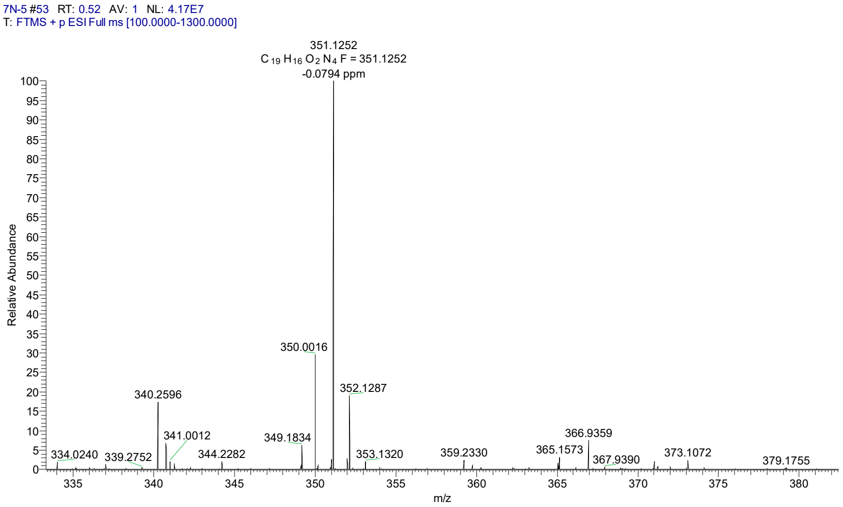

Figure S45. HR-MS Spectrum of 7o.

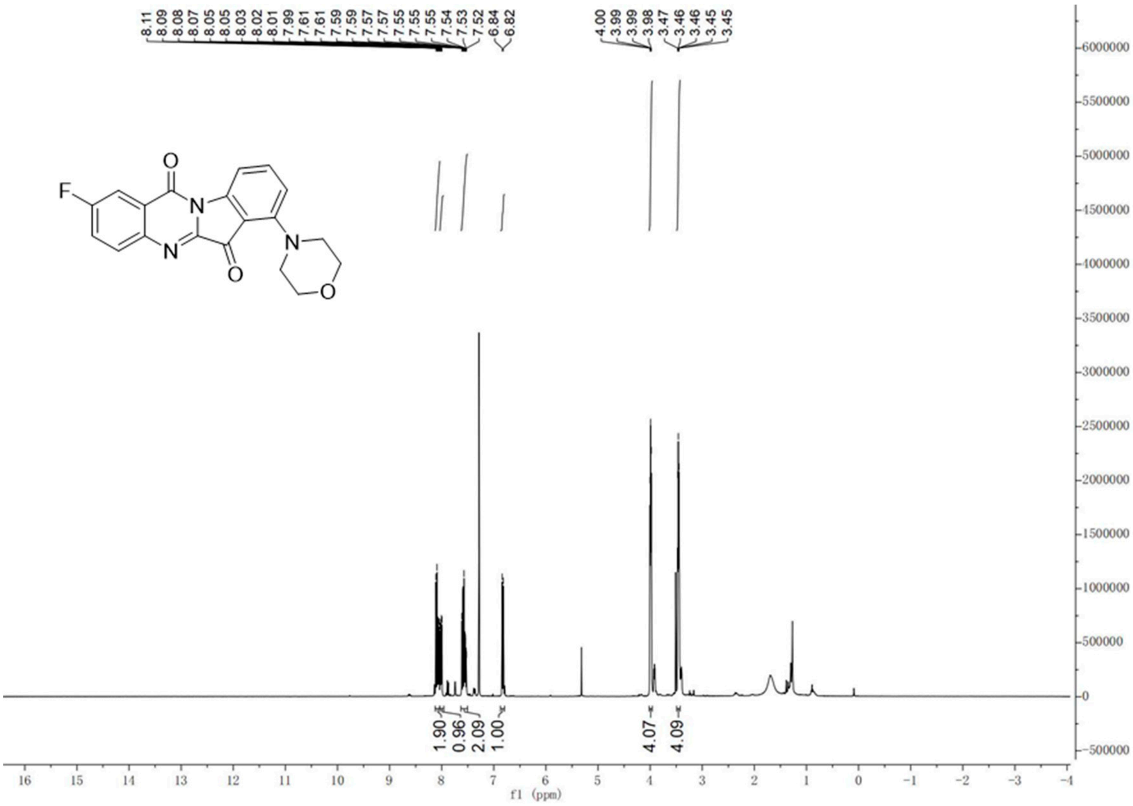

Figure S46. ^{1}H NMR Spectrum ($CDCl_3$, 400 MHz) of 7p.

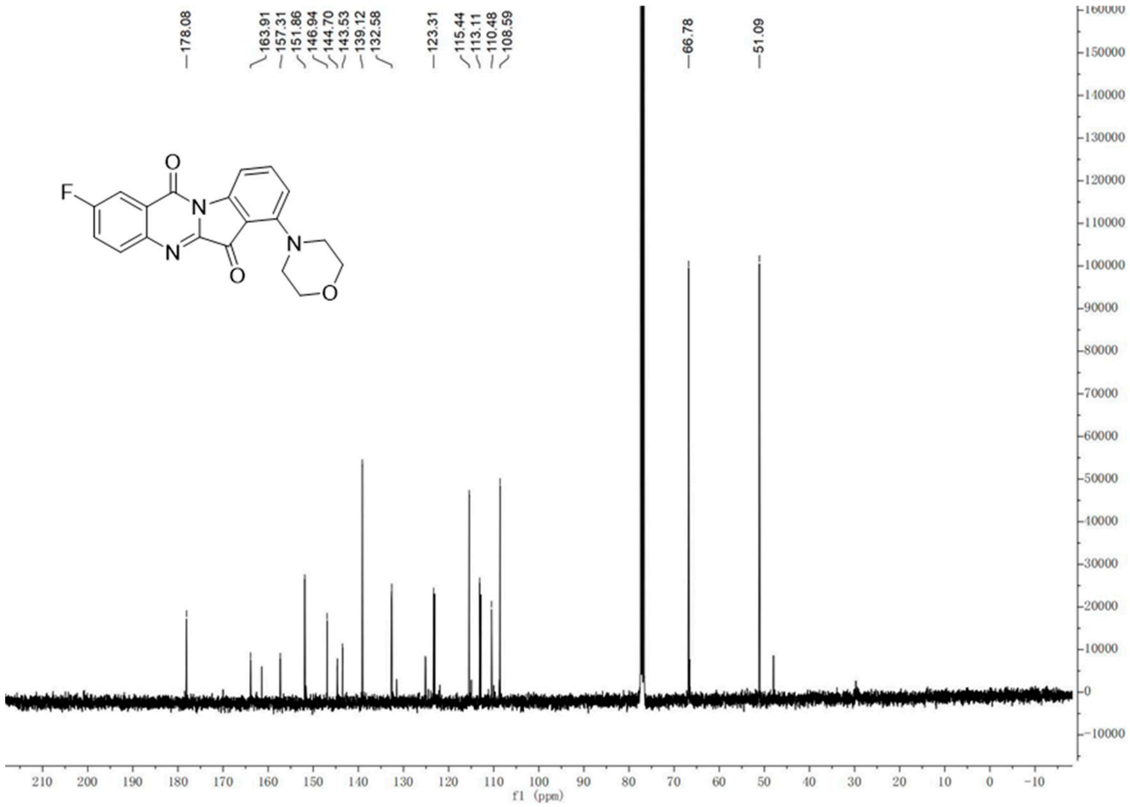

Figure S47. ^{13}C NMR Spectrum ($CDCl_3$, 101 MHz) of 7p.

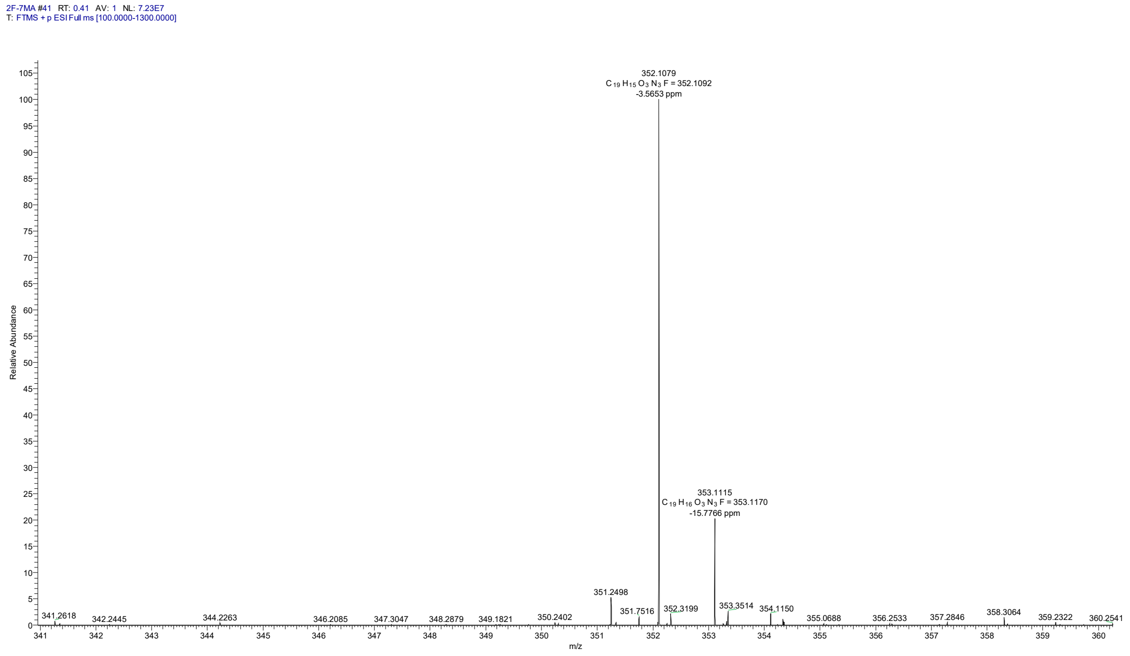

Figure S48. HR-MS Spectrum of 7p.

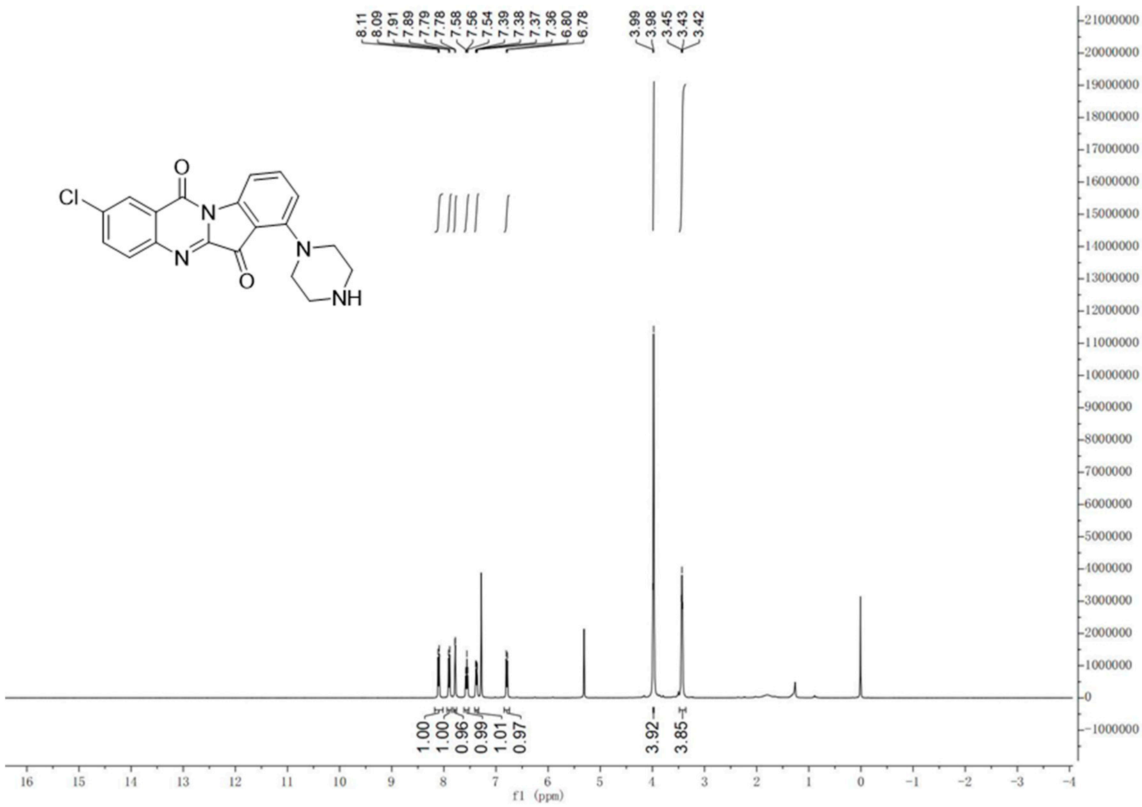

Figure S49. ^{1}H NMR Spectrum ($CDCl_3$, 400 MHz) of 7q.

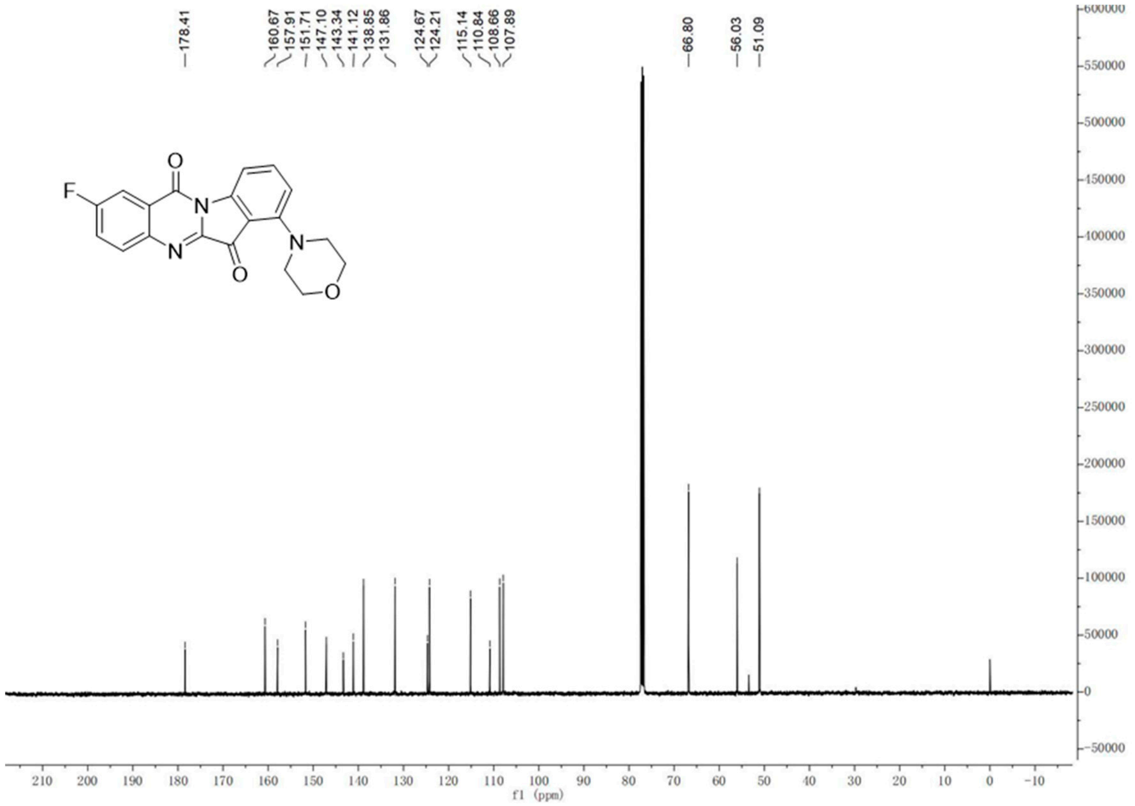

Figure S50. ^{13}C NMR Spectrum ($CDCl_3$, 101 MHz) of 7q.

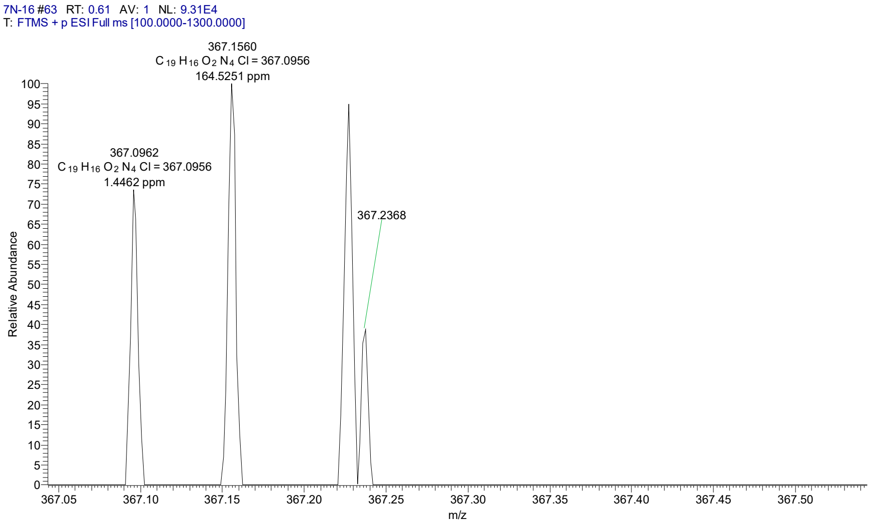

Figure S51. HR-MS Spectrum of 7q.

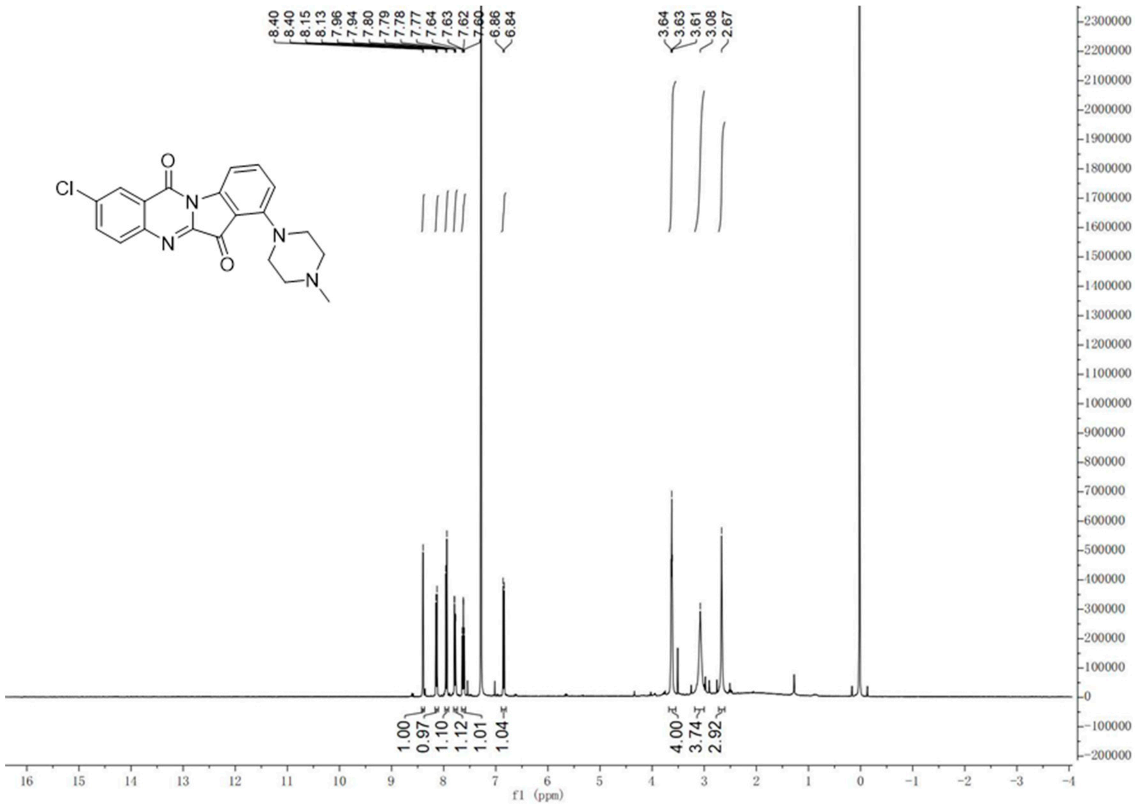

Figure S52. ^{1}H NMR Spectrum ($CDCl_3$, 400 MHz) of 7r.

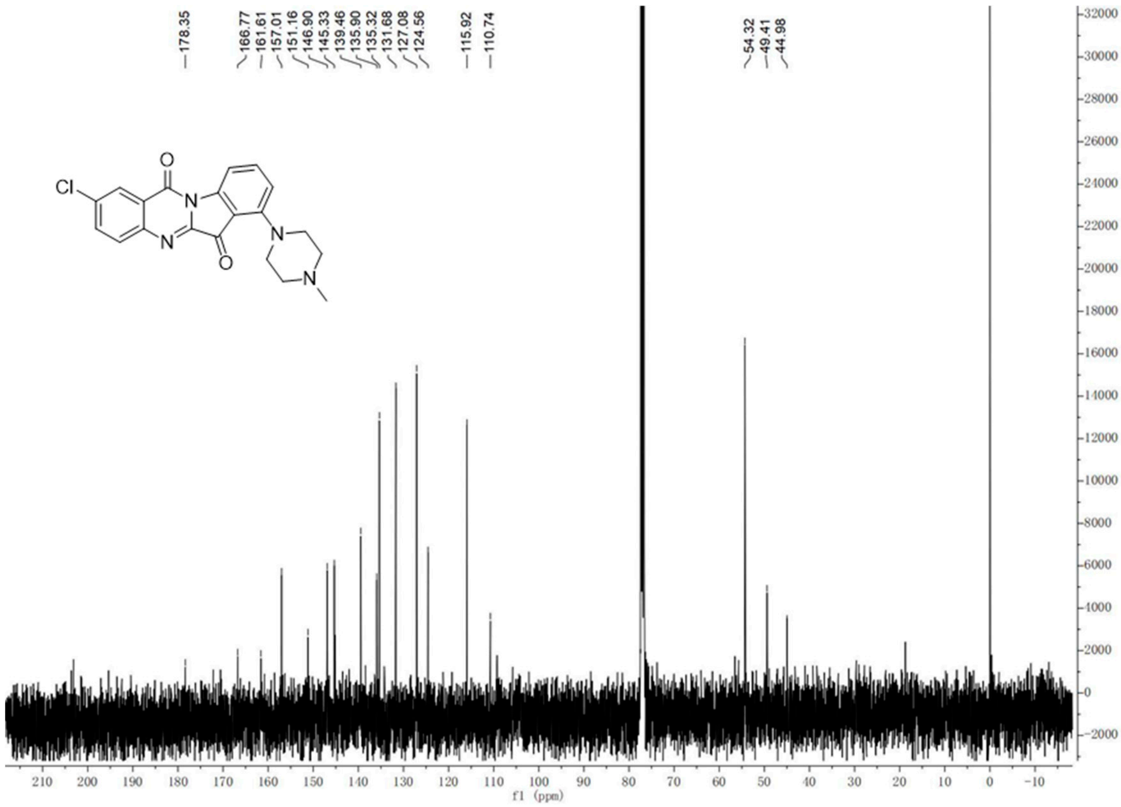

Figure S53. ^{13}C NMR Spectrum ($CDCl_3$, 101 MHz) of 7r.

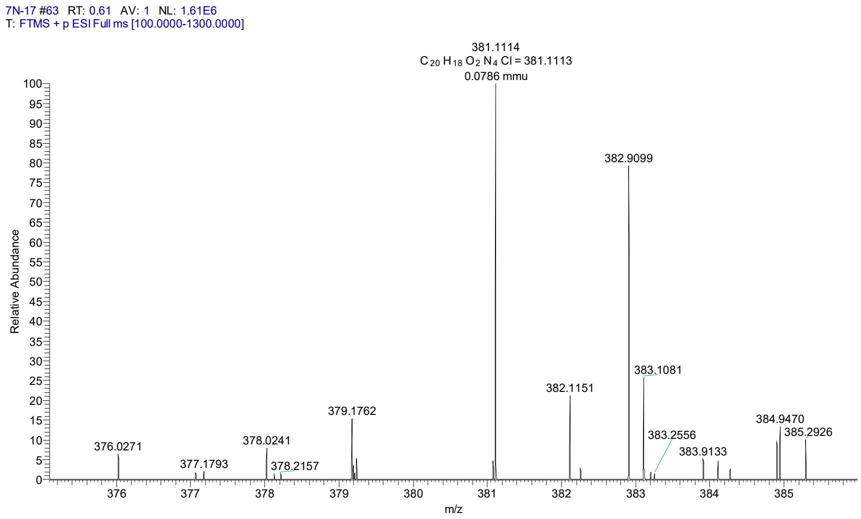

Figure S54. HR-MS Spectrum of 7r.

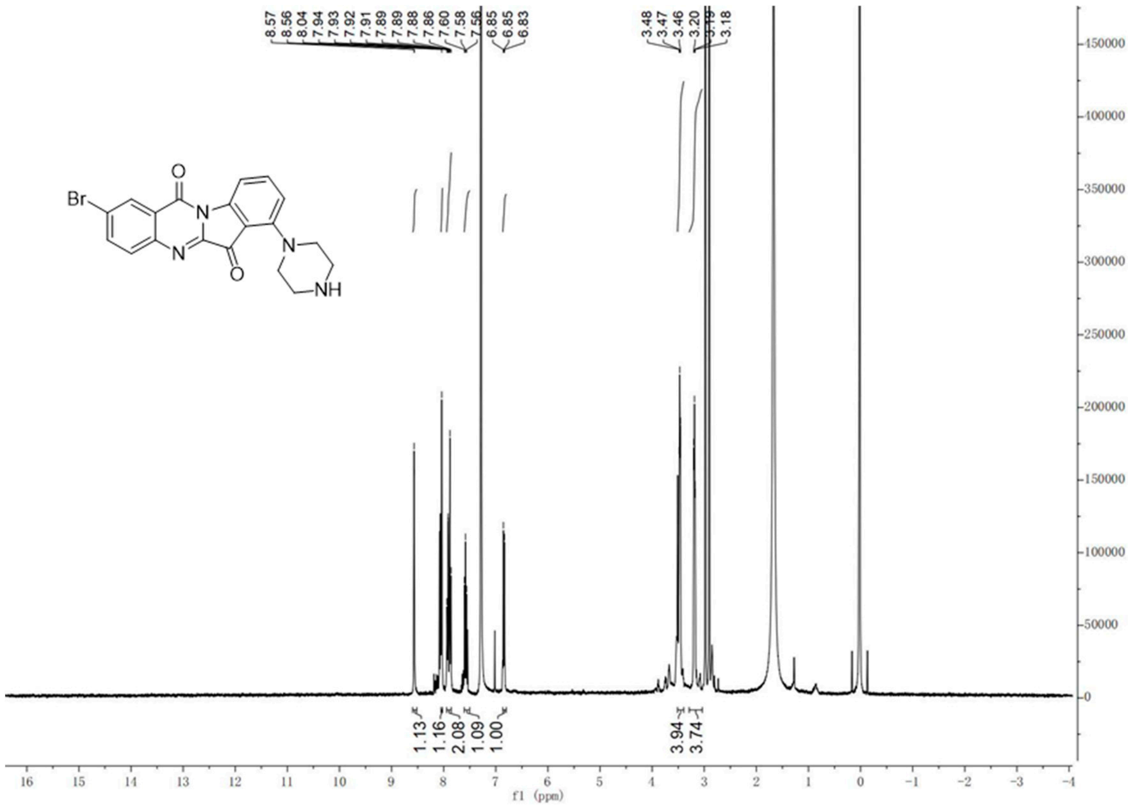

Figure S55. ^{1}H NMR Spectrum ($CDCl_3$, 400 MHz) of 7s.

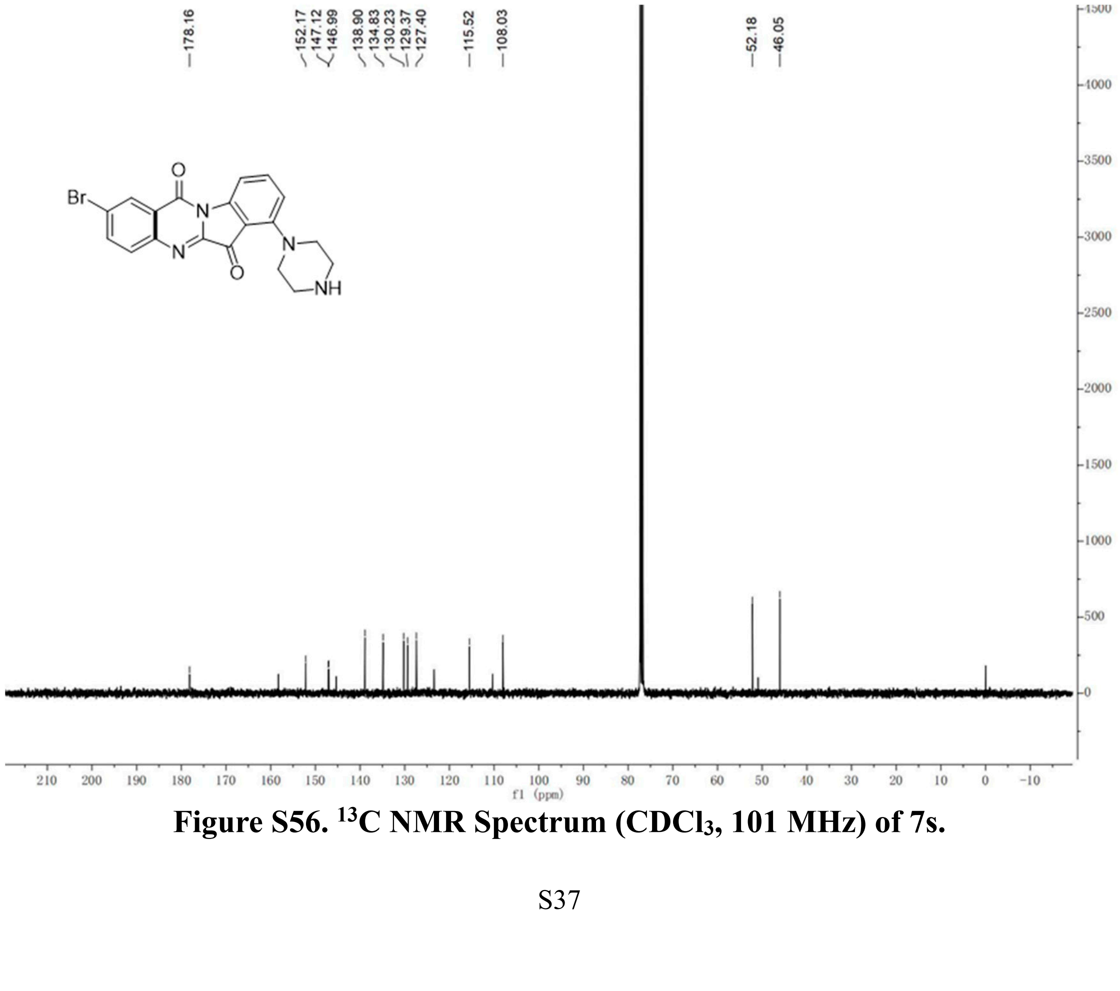

Figure S56. ^{13}C NMR Spectrum ($CDCl_3$, 101 MHz) of 7s.

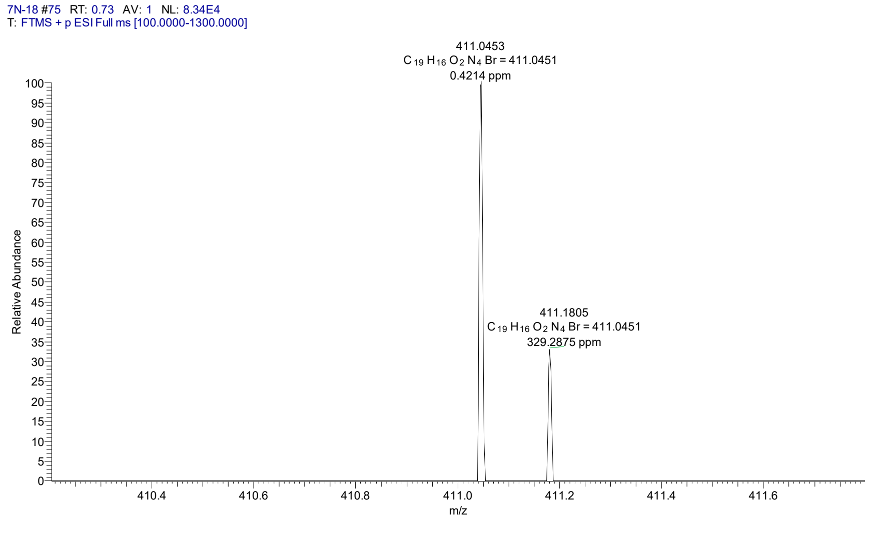

Figure S57. HR-MS Spectrum of 7s.

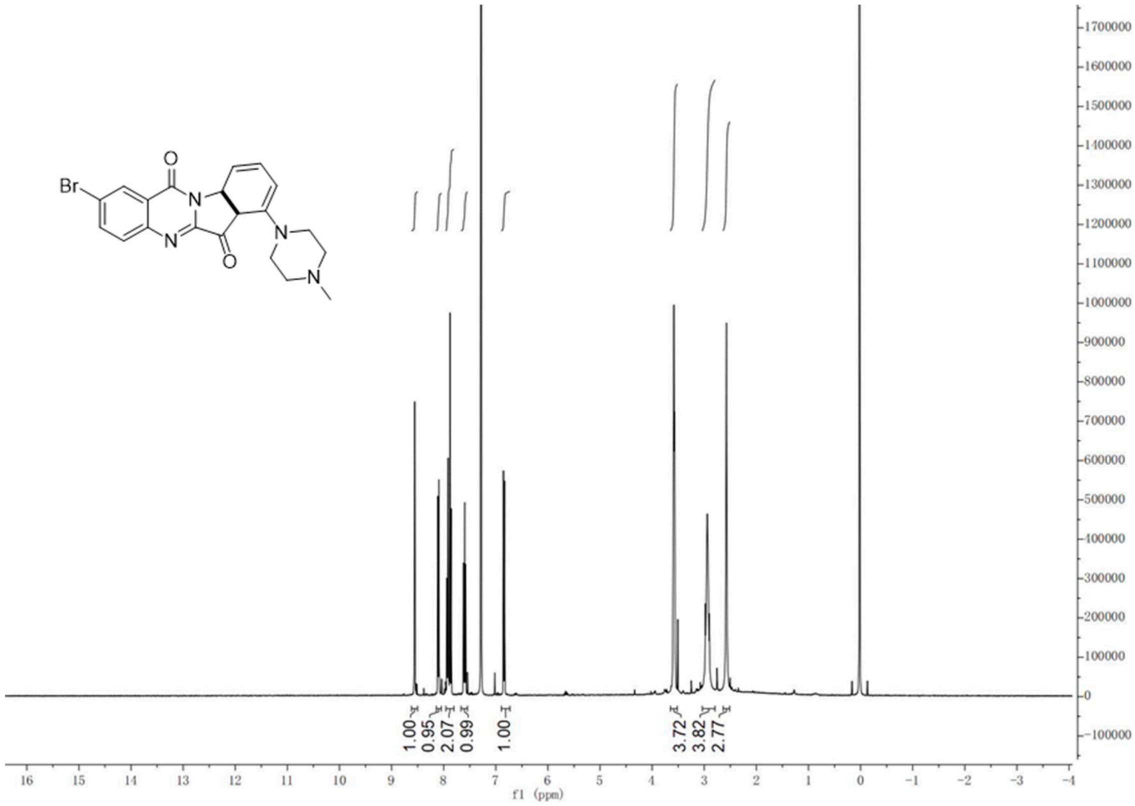

Figure S58. ^{1}H NMR Spectrum ($CDCl_3$, 400 MHz) of 7t.

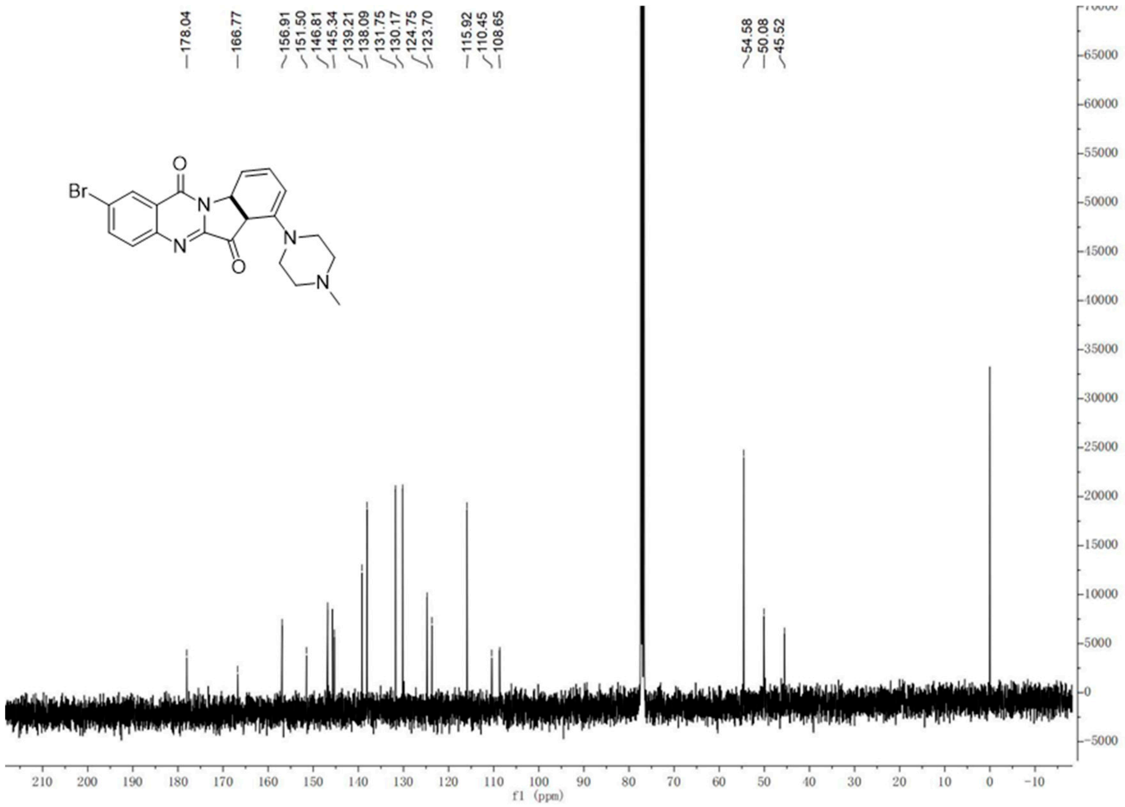

Figure S59. ^{13}**C NMR Spectrum (CDCl$_3$, 101 MHz) of 7t.**

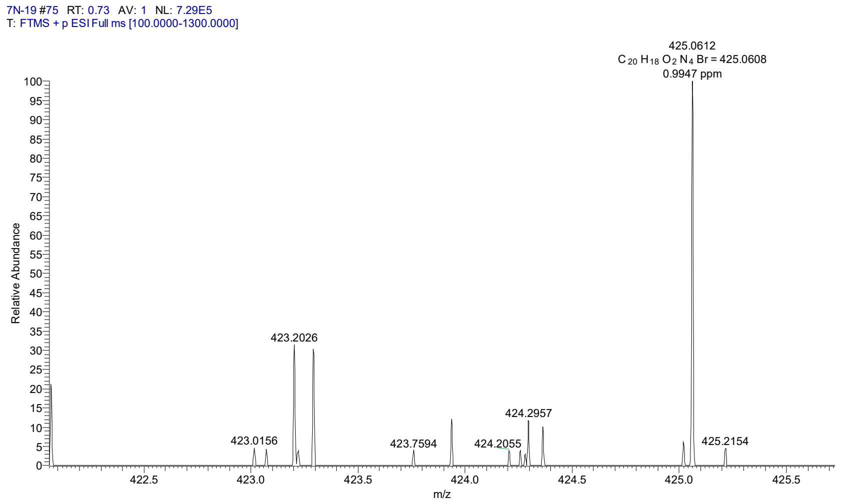

Figure S60. HR-MS Spectrum of 7t.

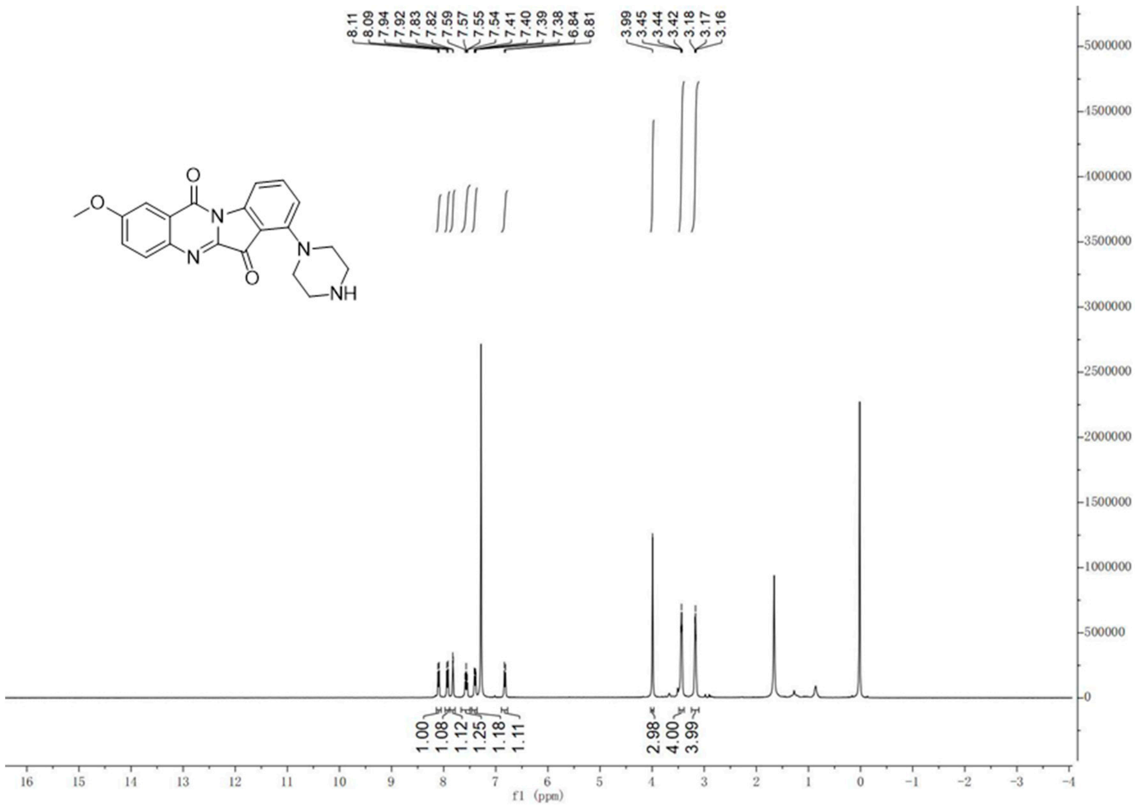

Figure S61. ^{1}H NMR Spectrum ($CDCl_3$, 400 MHz) of 7u.

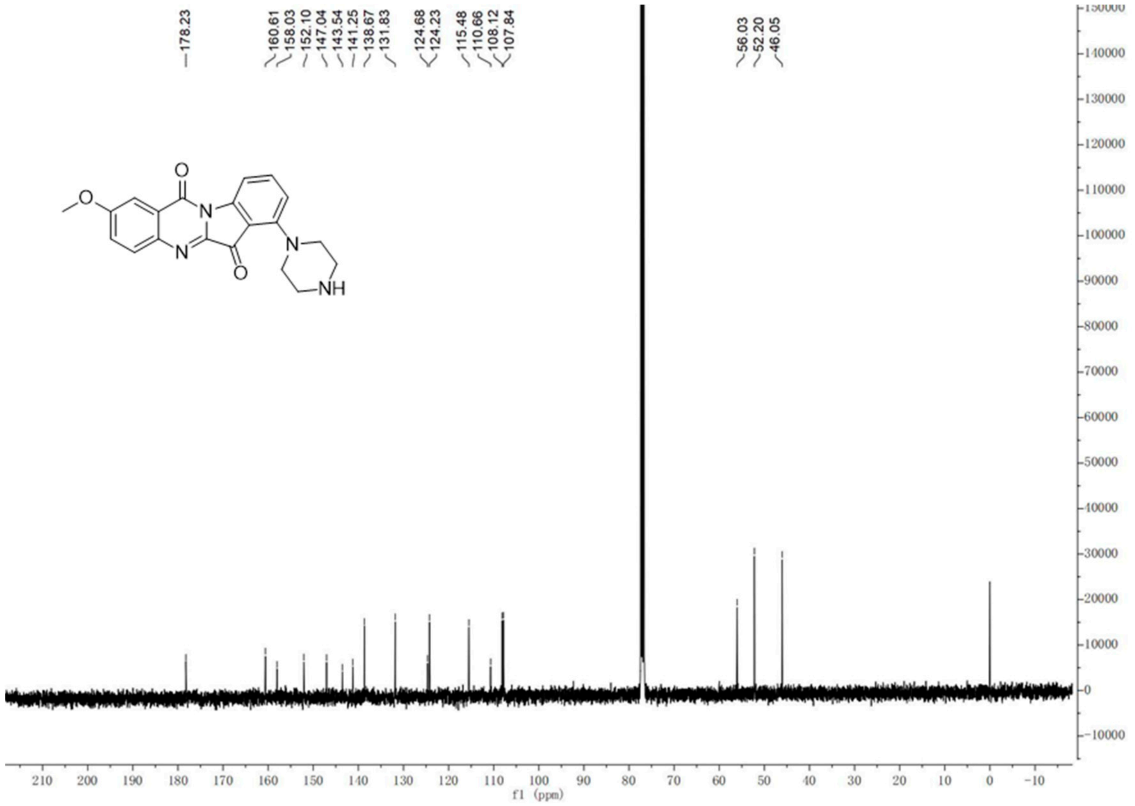

Figure S62. ^{13}C NMR Spectrum ($CDCl_3$, 101 MHz) of 7u.

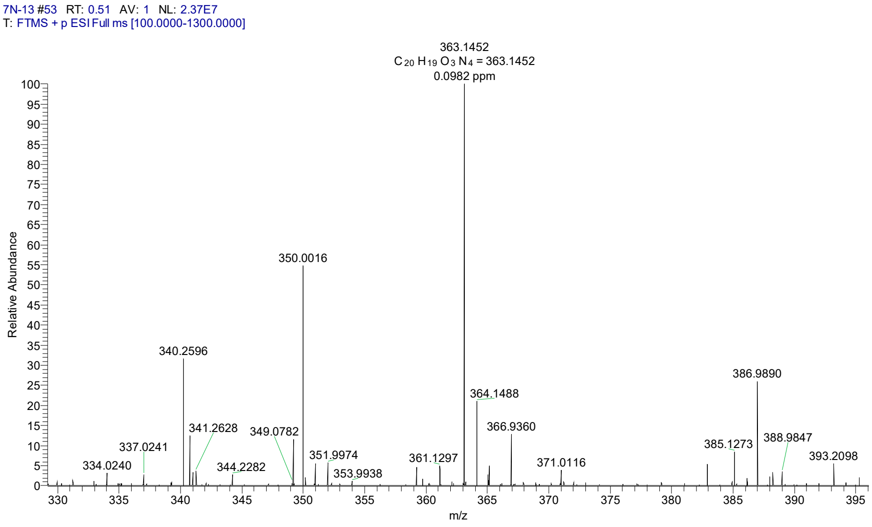

Figure S63. HR-MS Spectrum of 7u.

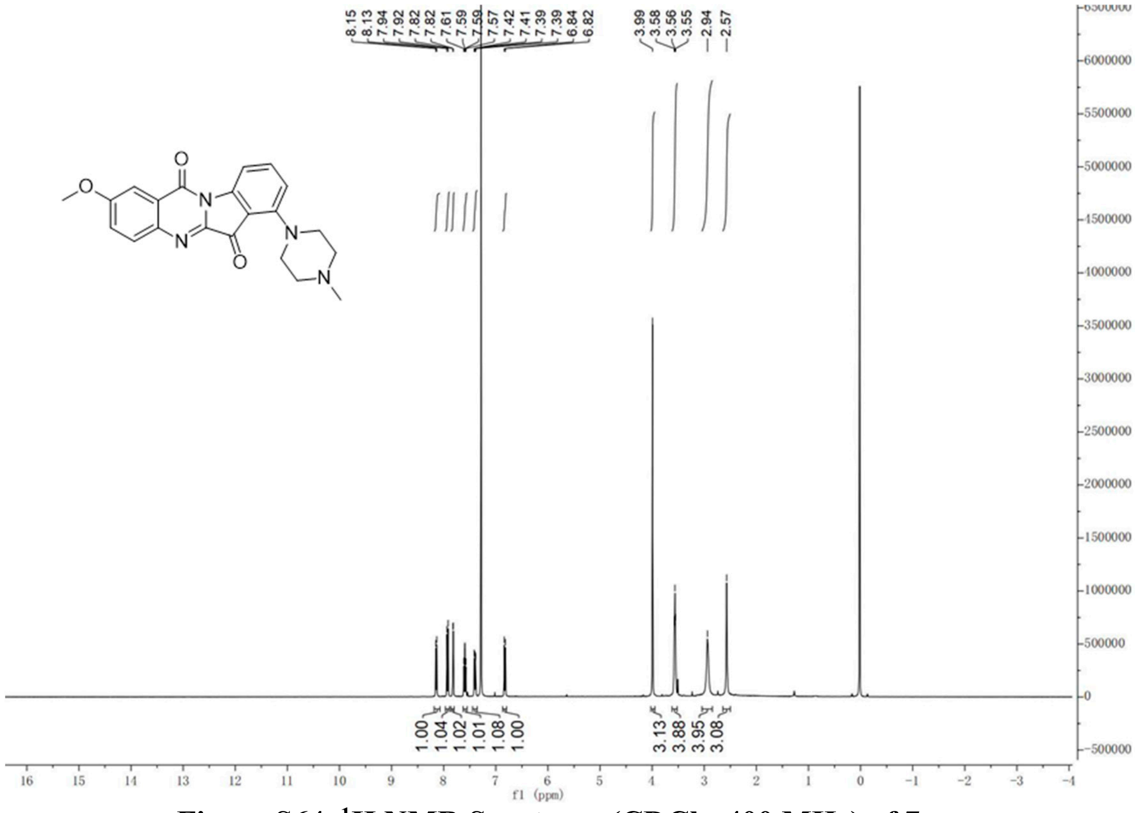

Figure S64. ^{1}H NMR Spectrum ($CDCl_3$, 400 MHz) of 7v.

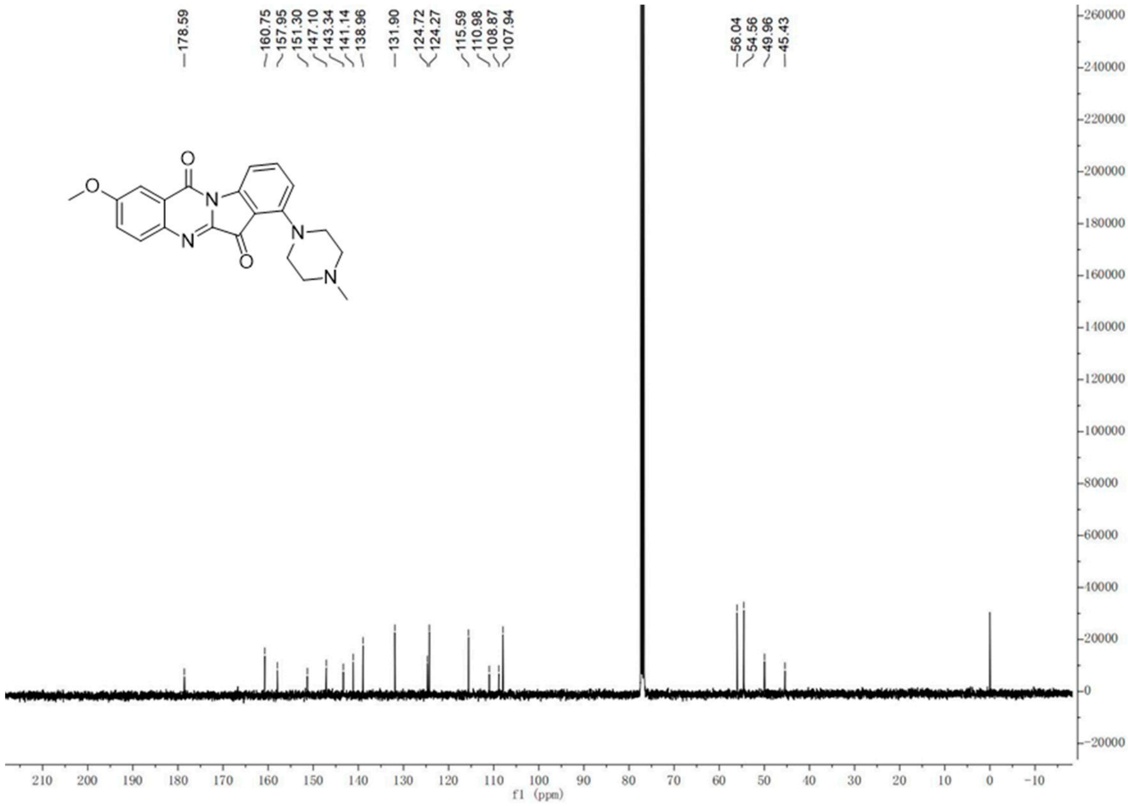

Figure S65. ^{13}C NMR Spectrum ($CDCl_3$, 101 MHz) of 7v.

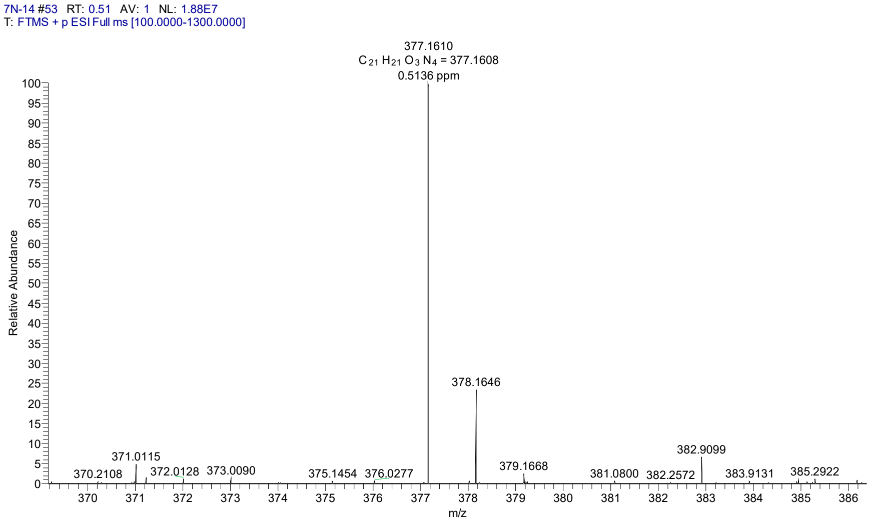

Figure S66. HR-MS Spectrum of 7v.

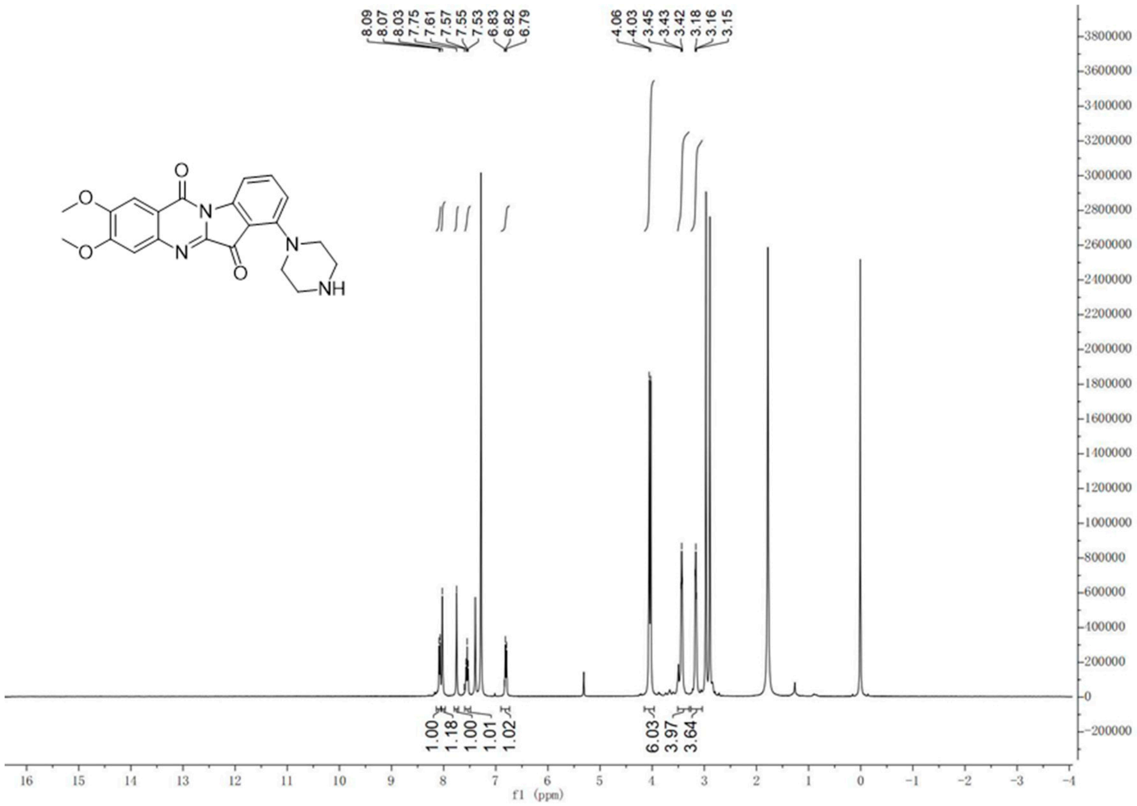

Figure S67. ^{1}H NMR Spectrum ($CDCl_3$, 400 MHz) of 7w.

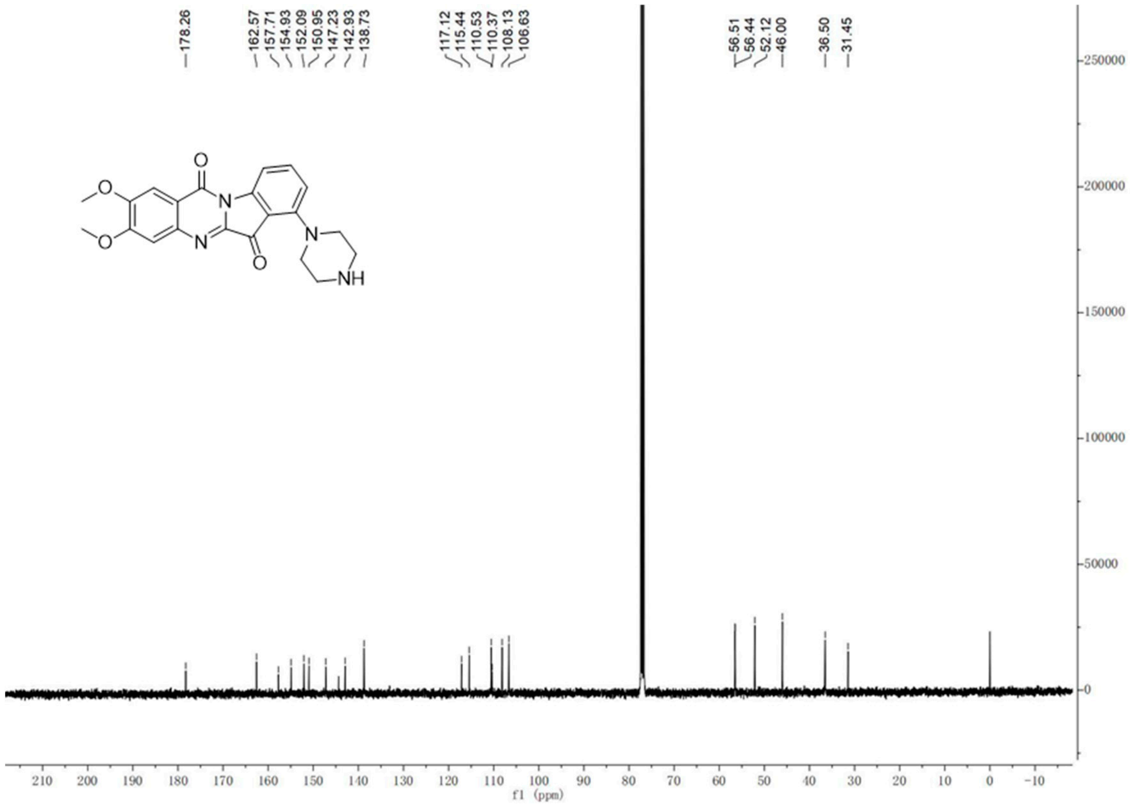

Figure S68. ^{13}C NMR Spectrum ($CDCl_3$, 101 MHz) of 7w.

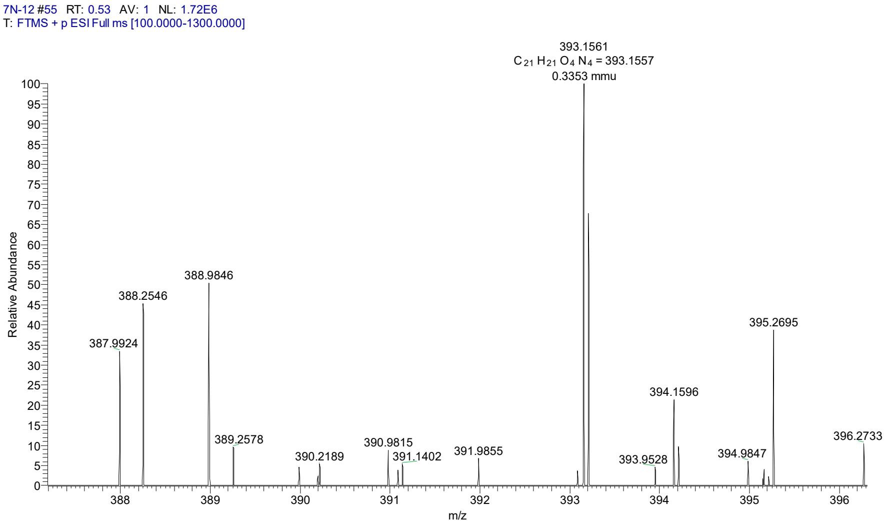

Figure S69. HR-MS Spectrum of 7w.

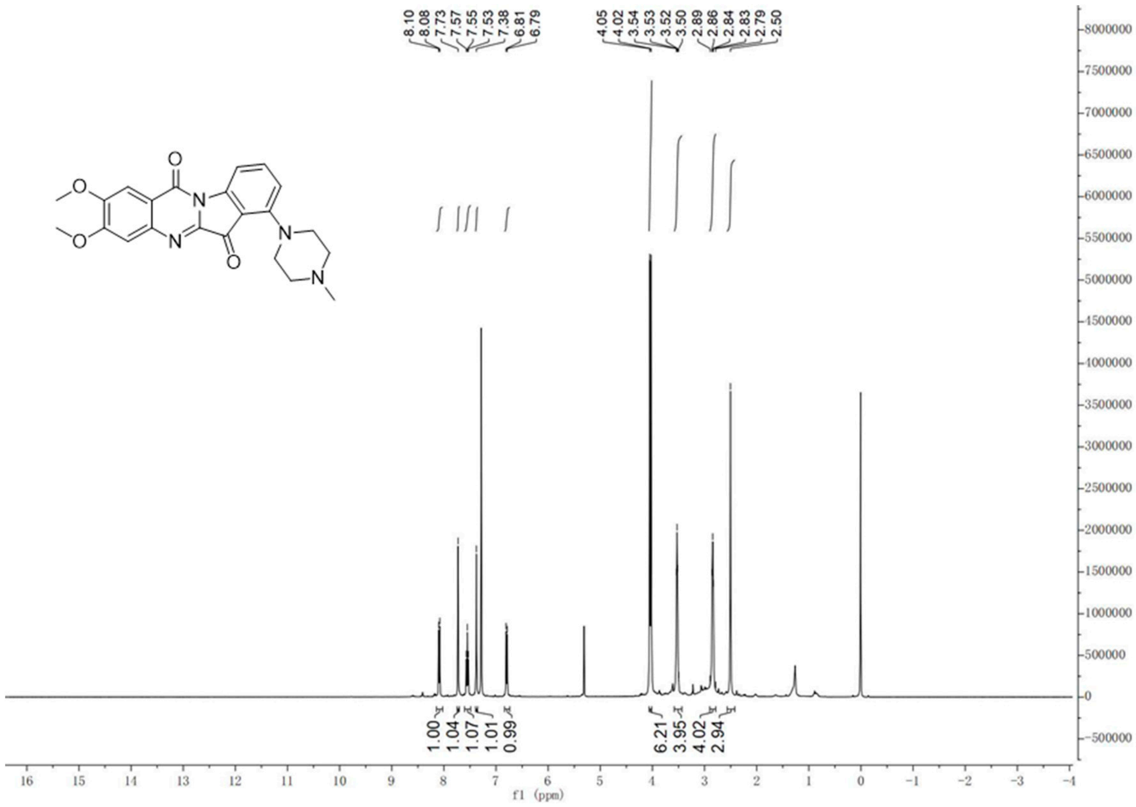

Figure S70. ^1H NMR Spectrum ($CDCl_3$, 400 MHz) of 7x.

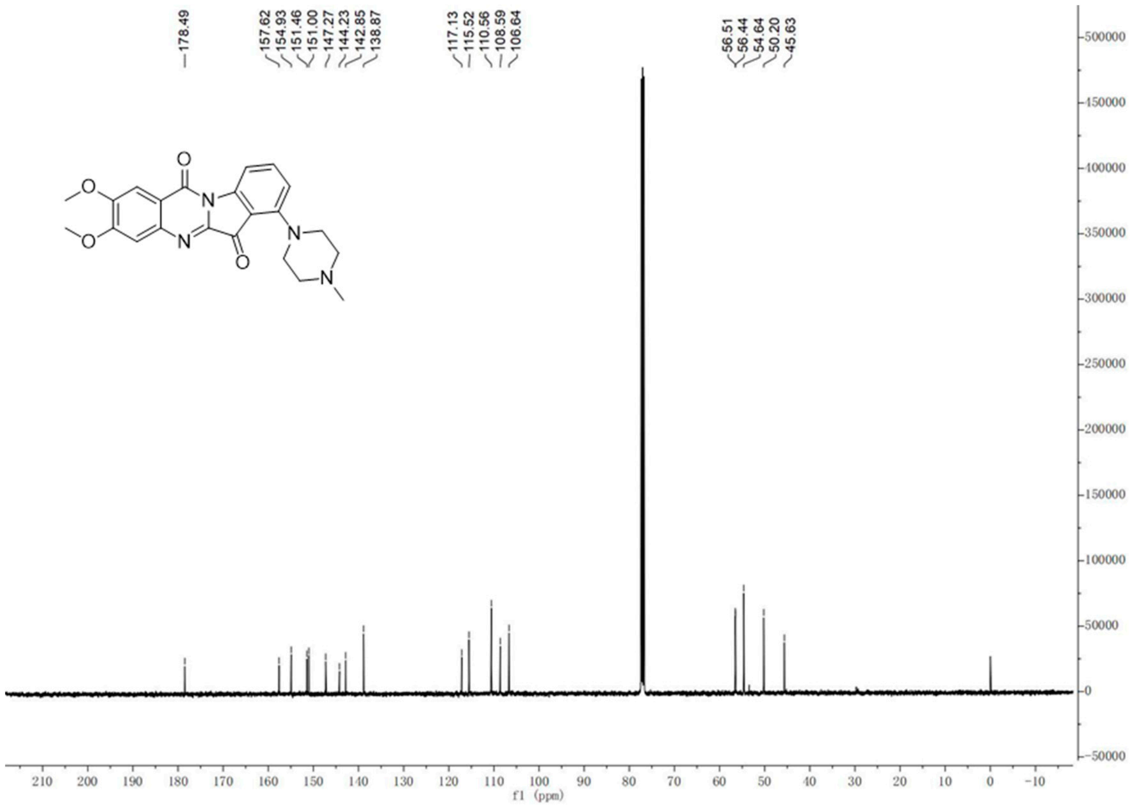

Figure S71. ^{13}C NMR Spectrum ($CDCl_3$, 101 MHz) of 7x.

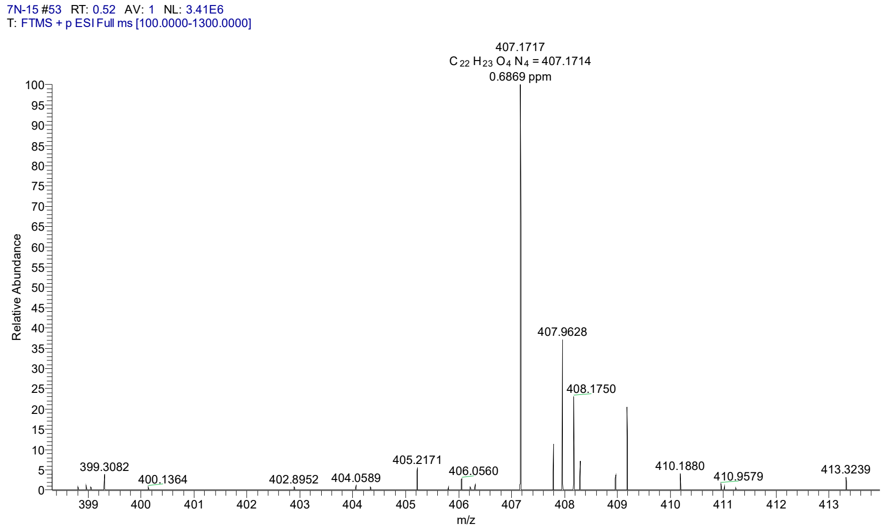

Figure S72. HR-MS Spectrum of 7x.

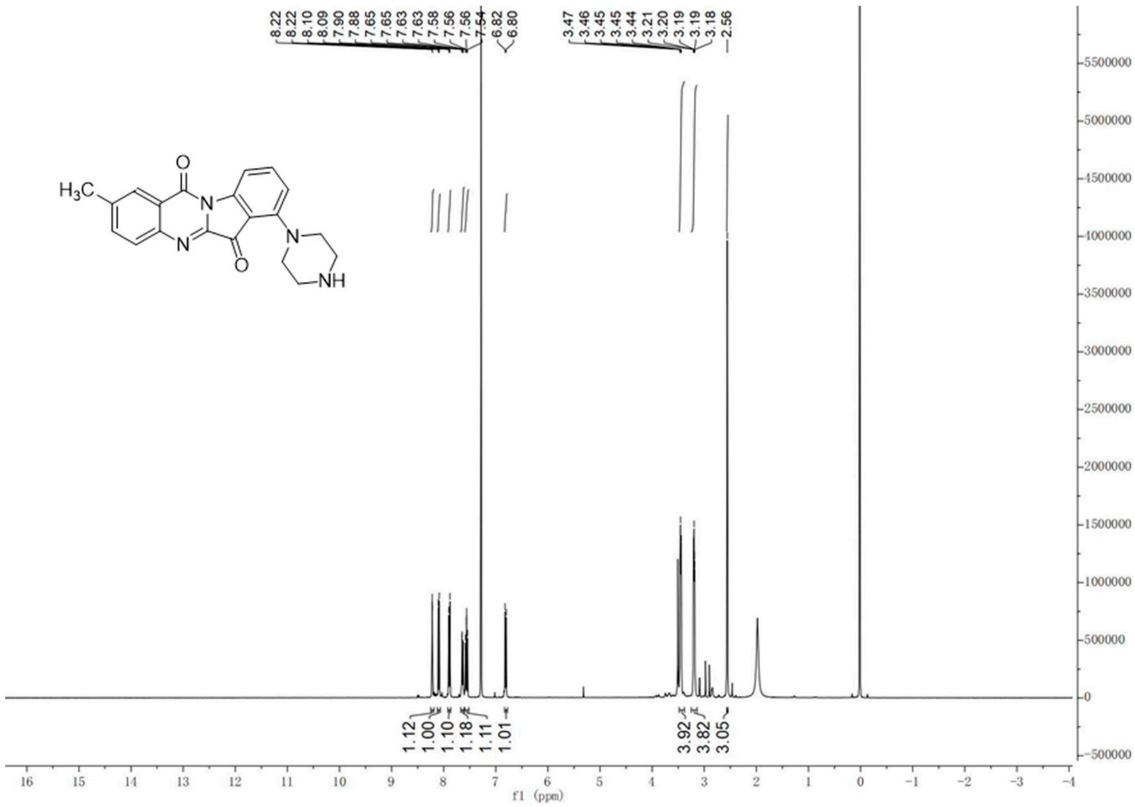

Figure S73. ^{1}H NMR Spectrum ($CDCl_3$, 400 MHz) of 7y.

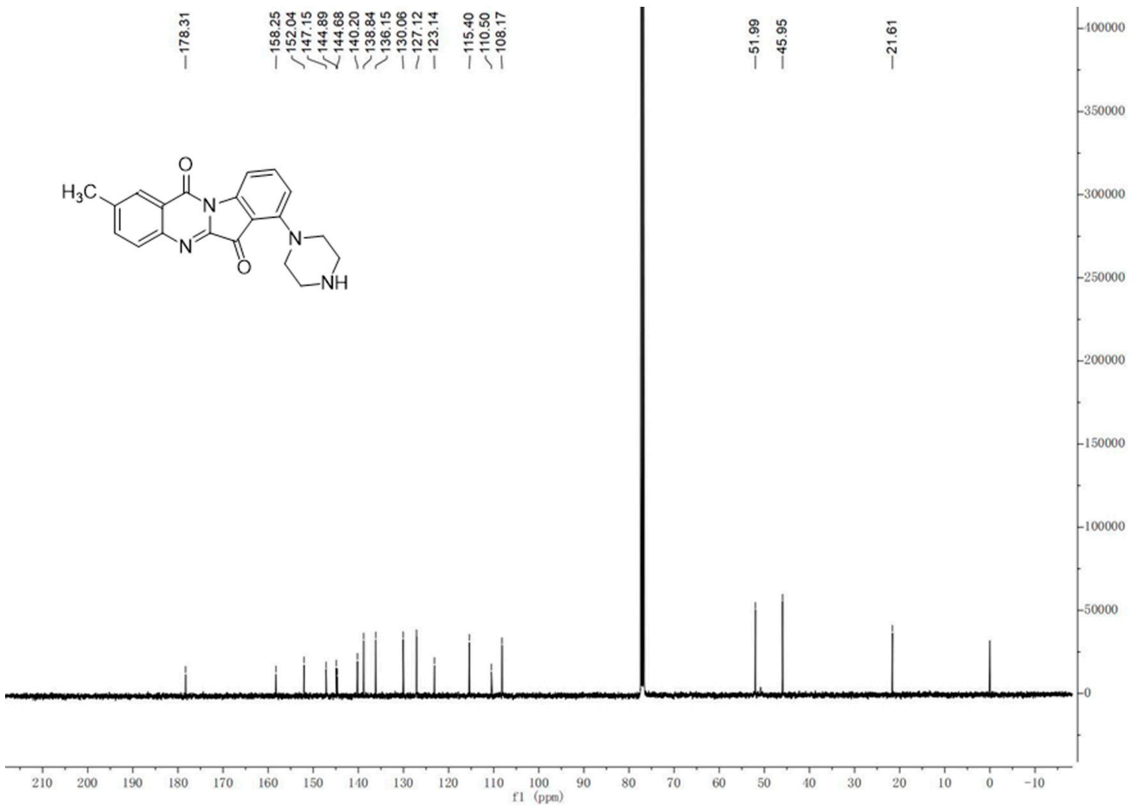

Figure S74. ^{13}C NMR Spectrum ($CDCl_3$, 101 MHz) of 7y.

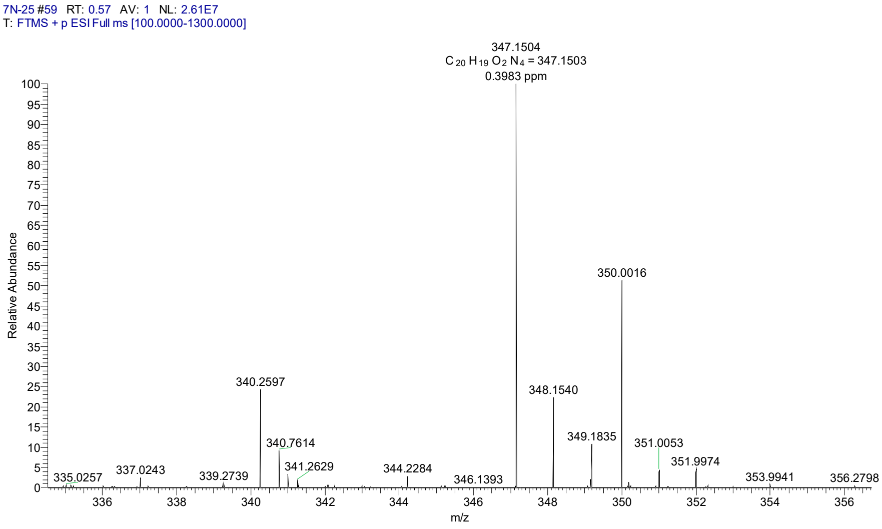

Figure S75. HR-MS Spectrum of 7y.

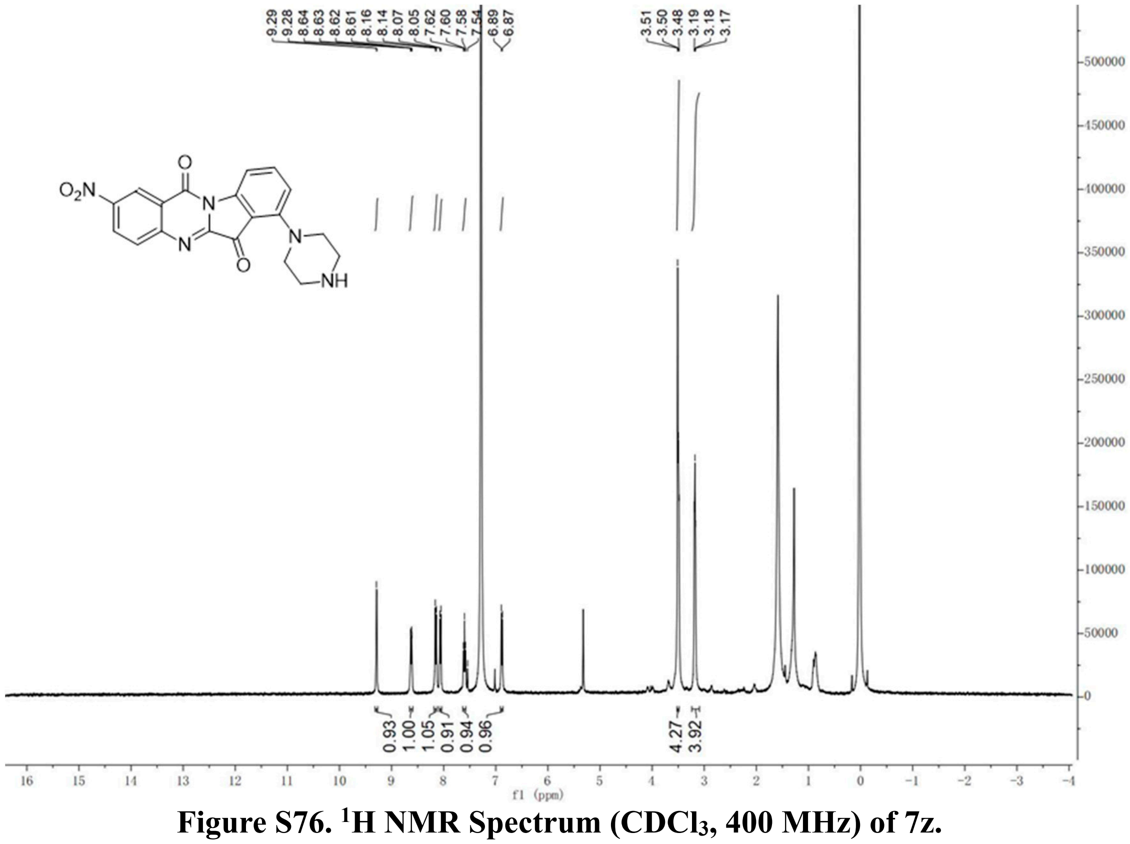

Figure S76. ^{1}H NMR Spectrum ($CDCl_3$, 400 MHz) of 7z.

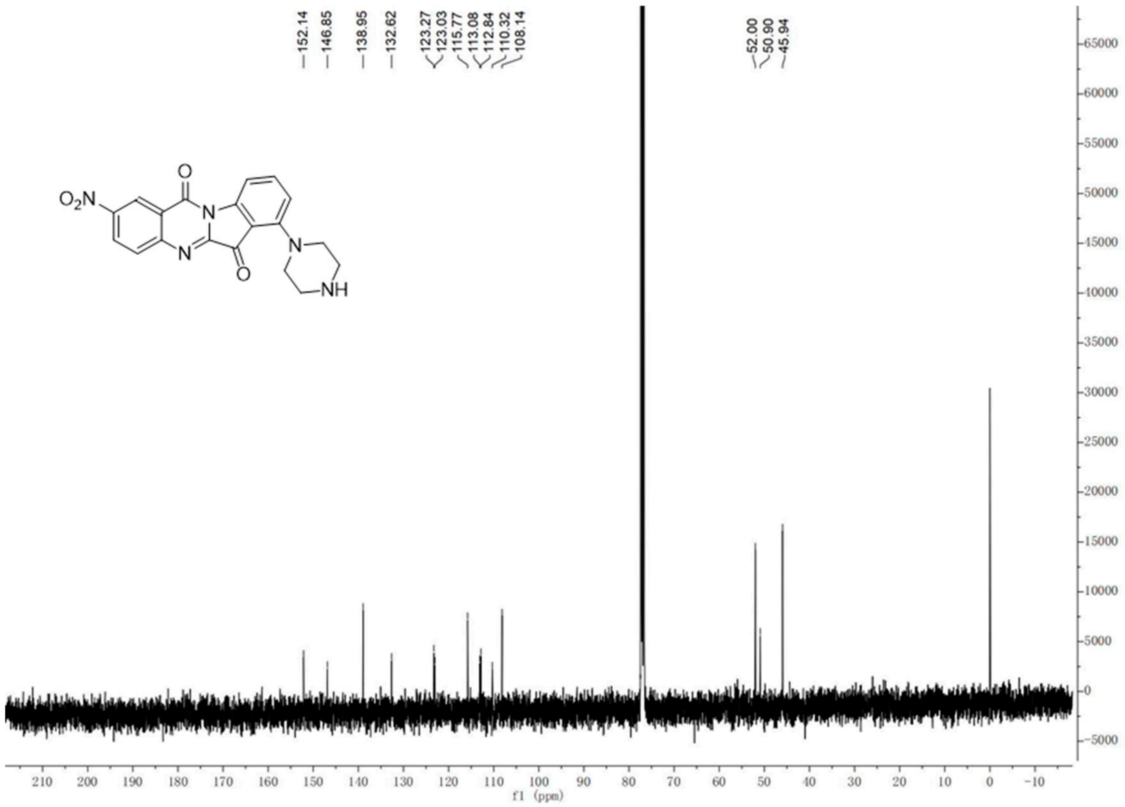

Figure S77. ^{13}C NMR Spectrum (CDCl$_3$, 101 MHz) of 7z.

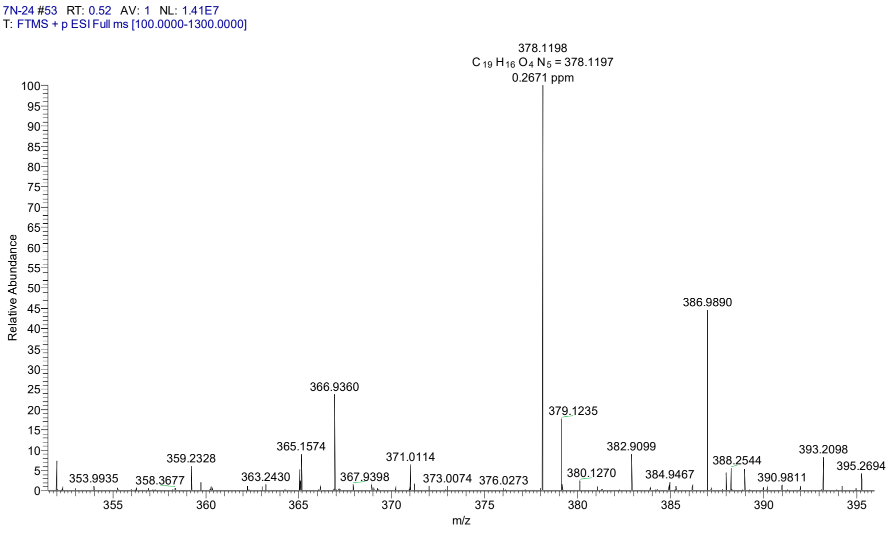

Figure S78. HR-MS Spectrum of 7z.
